# Identification of ultra-rare genetic variants in pediatric acute onset neuropsychiatric syndrome (PANS) by exome and whole genome sequencing

**DOI:** 10.1038/s41598-022-15279-3

**Published:** 2022-06-30

**Authors:** Rosario Trifiletti, Herbert M. Lachman, Olivia Manusama, Deyou Zheng, Alberto Spalice, Pietro Chiurazzi, Allan Schornagel, Andreea M. Serban, Rogier van Wijck, Janet L. Cunningham, Sigrid Swagemakers, Peter J. van der Spek

**Affiliations:** 1 The PANDAS-PANS Institute, Ramsey, NJ USA; 2 Department of Psychiatry and Behavioral Sciences, Albert Einstein College of Medicine, 1300 Morris Park Avenue, Bronx, NY 10461 USA; 3 Department of Medicine, Albert Einstein College of Medicine, Bronx, NY USA; 4 Department of Genetics, Albert Einstein College of Medicine, Bronx, NY USA; 5 Dominick P. Purpura Department of Neuroscience, Albert Einstein College of Medicine, Bronx, NY USA; 6 Department of Immunology, Erasmus MC, Rotterdam, The Netherlands; 7 Department of Neurology, Albert Einstein College of Medicine, Bronx, NY USA; 8 Department of Pediatrics, Pediatric Neurology, Sapienza University of Rome, Rome, Italy; 9 Sezione di Medicina Genomica, Dipartimento Scienze della Vita e Sanità Pubblica, Fondazione Policlinico Universitario A. Gemelli IRCCS, Università Cattolica del Sacro Cuore, Rome, Italy; 10 Dipartimento Scienze di Laboratorio e Infettivologiche, UOC Genetica Medica, Rome, Italy; 11 GGZ-Delfland, Kinderpraktijk Zoetermeer, Zoetermeer, The Netherlands; 12 Department of Pathology and Clinical Bioinformatics, Erasmus MC, Rotterdam, The Netherlands; 13 Department of Neuroscience, Psychiatry, Uppsala University, Uppsala, Sweden

**Keywords:** Genetics, Neuroscience

## Abstract

Abrupt onset of severe neuropsychiatric symptoms including obsessive–compulsive disorder, tics, anxiety, mood swings, irritability, and restricted eating is described in children with Pediatric Acute-Onset Neuropsychiatric Syndrome (PANS). Symptom onset is often temporally associated with infections, suggesting an underlying autoimmune/autoinflammatory etiology, although direct evidence is often lacking. The pathological mechanisms are likely heterogeneous, but we hypothesize convergence on one or more biological pathways. Consequently, we conducted whole exome sequencing (WES) on a U.S. cohort of 386 cases, and whole genome sequencing (WGS) on ten cases from the European Union who were selected because of severe PANS. We focused on identifying potentially deleterious genetic variants that were de novo or ultra-rare (MAF) < 0.001. Candidate mutations were found in 11 genes (*PPM1D, SGCE, PLCG2, NLRC4, CACNA1B, SHANK3, CHK2, GRIN2A, RAG1, GABRG2*, and *SYNGAP1*) in 21 cases, which included two or more unrelated subjects with ultra-rare variants in four genes. These genes converge into two broad functional categories. One regulates peripheral immune responses and microglia (*PPM1D, CHK2, NLRC4, RAG1, PLCG2*). The other is expressed primarily at neuronal synapses (*SHANK3, SYNGAP1, GRIN2A, GABRG2, CACNA1B, SGCE*). Mutations in these neuronal genes are also described in autism spectrum disorder and myoclonus-dystonia. In fact, 12/21 cases developed PANS superimposed on a preexisting neurodevelopmental disorder. Genes in both categories are also highly expressed in the enteric nervous system and the choroid plexus. Thus, genetic variation in PANS candidate genes may function by disrupting peripheral and central immune functions, neurotransmission, and/or the blood-CSF/brain barriers following stressors such as infection.

## Introduction

The acute onset of severe neuropsychiatric symptoms and abrupt loss of function in a child is an unusual and dramatic event. A clinical syndrome, Pediatric Acute Onset Neuropsychiatric Syndrome (PANS), includes a multitude of symptoms (Table 1)^1^. These fluctuate over time, and the onset, as well as “flares” are frequently in association with infections, which has led to speculations of autoimmune or autoinflammatory mechanisms^2,3^. Standard psychiatric care is often unable to sufficiently control the severe neuropsychiatric symptoms and, according to the Stanford PANS Clinic, the median Caregiver Burden Inventory score during a 1st PANS flare is higher than for those caring for someone with Alzheimer disease^4^. While there is a general recognition of the condition, a great deal of controversy remains concerning the validity of the PANS grouping with regards to common pathogenetic mechanisms and optimal treatment.

**Table 1:** Diagnostic criteria for PANS adapted from Cheng et al.^1^.

I. Abrupt onset of obsessive–compulsive disorder or severely restricted food intake
**II. Concurrent presence of additional neuropsychiatric symptoms with severe and acute onset from at least two of the following**
Anxiety
Emotional lability and/or depression
Irritability, aggression, and/or severely oppositional behaviors
Behavioral (developmental) regression
Deterioration in school performance (related to attention deficit/hyperactivity disorder [ADHD]-like symptoms, memory deficits, cognitive changes
Sensory or motor abnormalities
Somatic signs and symptoms, including sleep disturbances, enuresis, or urinary frequency
III. Symptoms are not better explained by a known neurologic or medical disorder

Complicating the matter, many patients with PANS have comorbid or a family history of neurodevelopmental and/or neuropsychiatric disorders. While some data suggests autoantibodies to neuronal antigens and elevated levels of inflammatory cytokines in PANS, there are inconsistencies^5–7^. Imaging studies in some PANS cases show local inflammation in the thalamus, basal ganglia, and amygdala, supporting a neuroinflammatory or autoimmune etiology^8^. Several reports indicate that patients may respond to immunomodulators, such as non-steroidal anti-inflammatory drugs (NSAIDs), intravenous immunoglobulin (IVIg), corticosteroids, and more recently, the B-cell inhibitor Rituximab^5,9,10^. However, there are few large, well-controlled clinical trials, with variable results^9,10^. Reluctance to administer immunomodulators remains due to these issues and the lack of consistent, objective biomarkers. Although most children with PANS show significant improvement at follow-up, according to a recently published longitudinal study, full remission was rare, and more than one-third were classified as having a chronic course^11^.

Heterogeneity on several fronts is a plausible explanation for the inconsistent findings regarding autoantibodies, inflammatory markers, and the relationship to infectious pathogens. *Streptococcus pyogenes, Borrelia burgdorferi*, and influenza virus are examples of potential autoimmune or proinflammatory triggers^4–7^. Non-infectious environmental factors that activate innate immune pathways (i.e., sterile or non-infectious inflammation), such as oxidative stress, toxin exposure, or emotional stress, could initiate abnormal inflammatory responses in genetically susceptible children. Finally, heterogeneity in genetic risk factors that cause dysregulation of peripheral immunity, and/or central neuronal/innate immune pathways, could be at play, all leading to a common clinical phenotype. In this model, some genetic subgroups will express classic markers of inflammation or autoantibodies, but others won’t. However, gene discovery in PANS that can test this model is still in its infancy.

Whole exome sequencing (WES) and whole genome sequencing (WGS) are gene discovery tools used by researchers, and increasingly by clinicians, to identify ultra-rare, biologically powerful genetic factors underlying disease states. However, such studies have not yet been reported in PANS. We now describe the discovery of ultra-rare variants in 11 genes in 21 PANS cases using these sequencing strategies.

## Results

**Figure 1:**
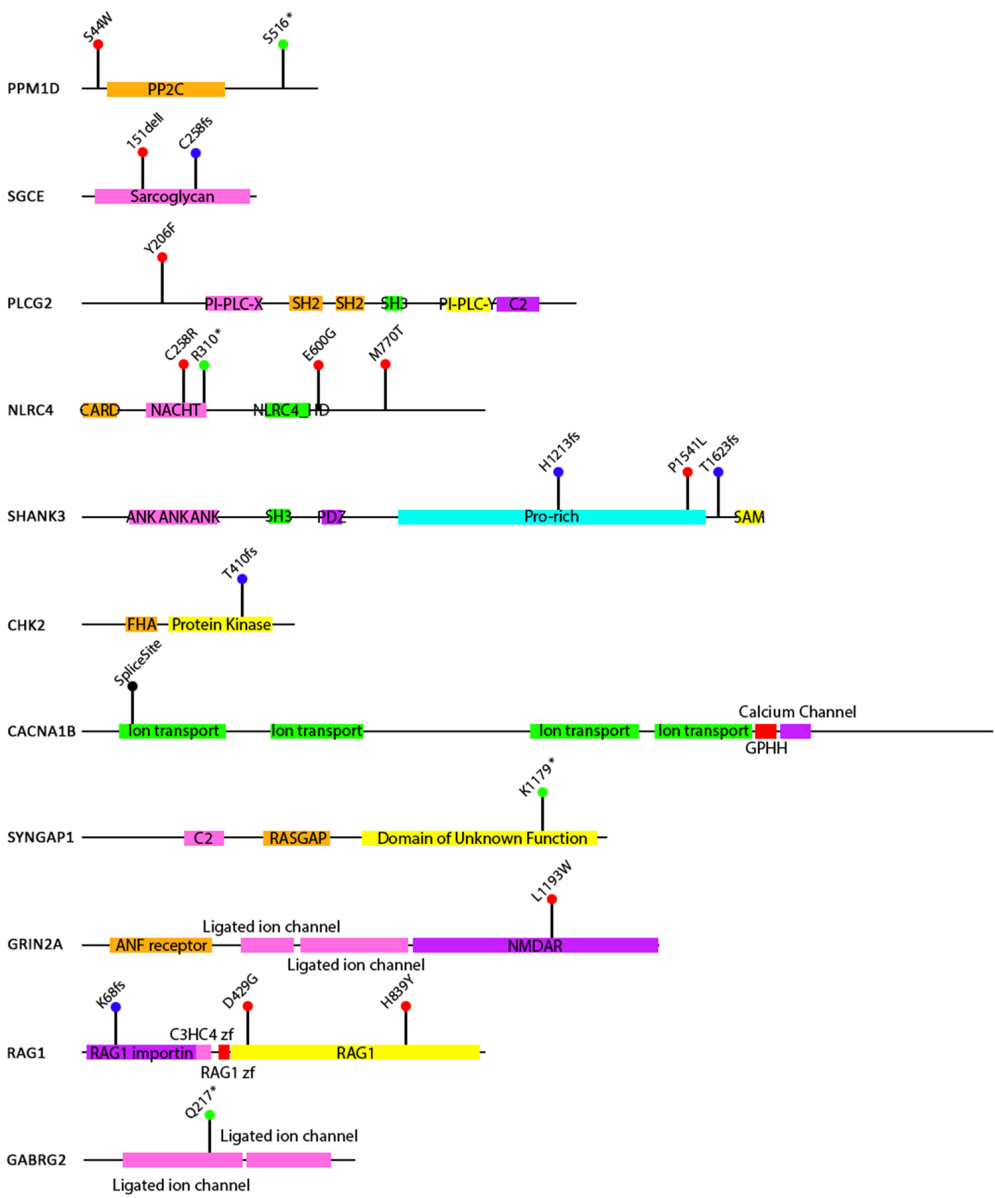
Position of PANS ultra-rare variants within each candidate gene.

Ultra-rare variants were found in 11 genes: *PPM1D* (3 cases), *NLRC4* (4 cases), *RAG1* (3 cases), *SGCE* (2 cases), *CACNA1B* (2 cases), *SHANK3* (3 cases), *PLCG2*, *CHK2* (also known as CHEK2), *GRIN2A, GABRG2,* and *SYNGAP1* in 21 patients with PANS. Among the *NLRC4* and *SHANK3* cases is one individual, case 19, in whom an ultra-rare variant was found in both genes (*SHANK3*, c.4622C>T, P1541L; *NLRC4*, c.928C>T, p.310X). The ultra-rare variants and brief descriptions of the cases are shown on Fig. 1 and Table 2, respectively. All of the cases were heterozygous for the variants. In the European cohort, two affected siblings (Cases 1 and 2) were found with an ultra-rare missense mutation in *PPM1D* (c.131C>G; p.S44W). Case 1 was treated with and responded to IVIg treatment. They inherited the variant from an asymptomatic parent who has a family history of autoimmune disorders, a common scenario in PANS families^12,13^. S44W is a known single nucleotide variant (SNV), rs373862041, with a minor allele frequency (MAF) of 0.00026, based on the TOPMed database of more than 125,000 samples^14^. The ultra-rare variant in *CACNA1B,* found in two affected siblings (Cases 4 and 5), is a 48 bp insert at the exon 2 splice donor site (c.390 + 1) that is predicted to disrupt splicing. It is part of a set of multiallelic insertion variants, rs370237172, that has an overall MAF of 0.017. However, the 48 bp insert is ultra-rare and has not been observed in control data sets. We have been unable to genotype the parents. Interestingly, the same 48 bp insert was also identified in a young woman who has been incapacitated with chronic fatigue syndrome (myalgic encephalomyelitis/chronic fatigue syndrome) and anorexia (unpublished observations). Known transmission from a parent in the European cohort was also found in Cases 10 and 11 (*NLRC4*: c.772 T>C; p.C258R; and *SGCE*: del150 Iso; c.450_452, respectively. Case 10 inherited the mutation from a mother who has a history of psoriasis and arthritis. She was diagnosed with focal epilepsy as an adult but has no history of PANS. A younger sibling with the same variant has a history suggestive of PANS but this has not yet been clinically ascertained. Two siblings of Case 11 (*SGCE* (del150 Iso; c.450_452) also inherited the variant. One has asthma, allergies, and contact dermatitis, but no neuropsychiatric problems. The other has similar atopic problems and debilitating cluster-type headaches.

**Table 2:**
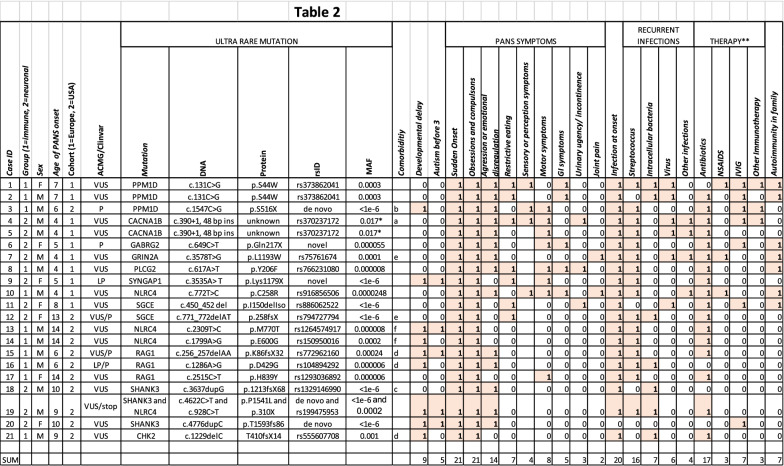
Summary of cases.

Patient demographics, genetic variants, and brief clinical description.

*ACMG* American College of Medical Genetics, *VUS* variant of unknown or uncertain significance, *LP* likely pathological, *P* pathological, *MAF* minor allele frequency, *OCD* obsessive compulsive disorder, *GI* gastrointestinal.

**Denotes treatment with clinical response for PANS symptoms. Comorbidities: a: psoriasis and hematological malignancy, b: Jansen de Vries Syndrome, c: Phelan-McDermid syndrome, d: Mannose-binding lectin deficiency, e: dyslexia, f: atrial septal defect, marfanoid body habitus. Infections include Streptococcus (*Streptococcus pneumoniae*, and group A, beta-hemolytic streptococcus), intracellular bacteria (Mycoplasma, Borrelia), and viruses (Herpes, Influenza).

For the other ultra-rare variants, parental genotypes were not available. Remarkably, in the U.S. cohort, different ultra-rare variants were found in two of the same genes identified in the European cohort (*NLRC4* and *SGC2*), and another, *CHK2*, codes for a well-established PPM1D substrate^15^.

In summary, all 21 cases (14 boys and 7 girls) described sudden onset of symptoms and in 20 cases these could be temporally associated to one or several infections (streptococcus (n = 16), intracellular bacteria (n = 7), or virus (n = 6) (Table 2). Mean age of PANS symptom onset was 7.15 years old (SD = 3.73). Their symptoms included OCD (n = 21), aggression or emotional dysregulation (n = 14), restrictive eating (n = 7), sensory or perception symptoms (n = 7), motor symptoms (n = 8), GI symptoms (n = 5), urinary urgency/incontinence (n = 3) and joint pain (n = 2). All seven cases who had received IVIg therapy reported partial or complete symptom reduction.

Cases 3 (*PPM1D*), 9 (*SYNGAP1*), 13 and 14 (*NLRC4*), 15 and 16 (*RAG1*), 19 and 20 (*SHANK3*), and 21 (*CHK2*) also had preexisting ASD or developmental delay prior to developing PANS. Case 3 had PANS characterized by severe OCD triggered by Streptococcus infections which improved with IVIg and plasmapheresis and was subsequently diagnosed with Jansen de Vries Syndrome (JdVS) based on a typical truncating mutation in *PPM1D* exon 6 (c.1547 C>G; p.S516X)^16^. Case 9 has a nonsense mutation in *SYNGAP1* codon 1179 and was diagnosed with ASD as a child. Case 9 developed acute onset OCD and food restrictions following a culture positive Streptococcus pharyngitis. Case 18 (*SHANK3*: c.3637dupG, p.1213fsX68), who was diagnosed with Phelan-McDermid syndrome, which is usually caused by *SHANK3* deletions, developed the sudden onset of severe OCD following bacterial infections. Case 20 (*SHANK3*: c.4776dupC, p.T1593fsX86) also had a history ASD with minimal verbal ability since early childhood. Case 20 developed the sudden onset of OCD and aggression as a child that improved dramatically with IVIg, although the baseline ASD and poor verbal ability did not improve.

### Connectivity network

**Figure 2:**
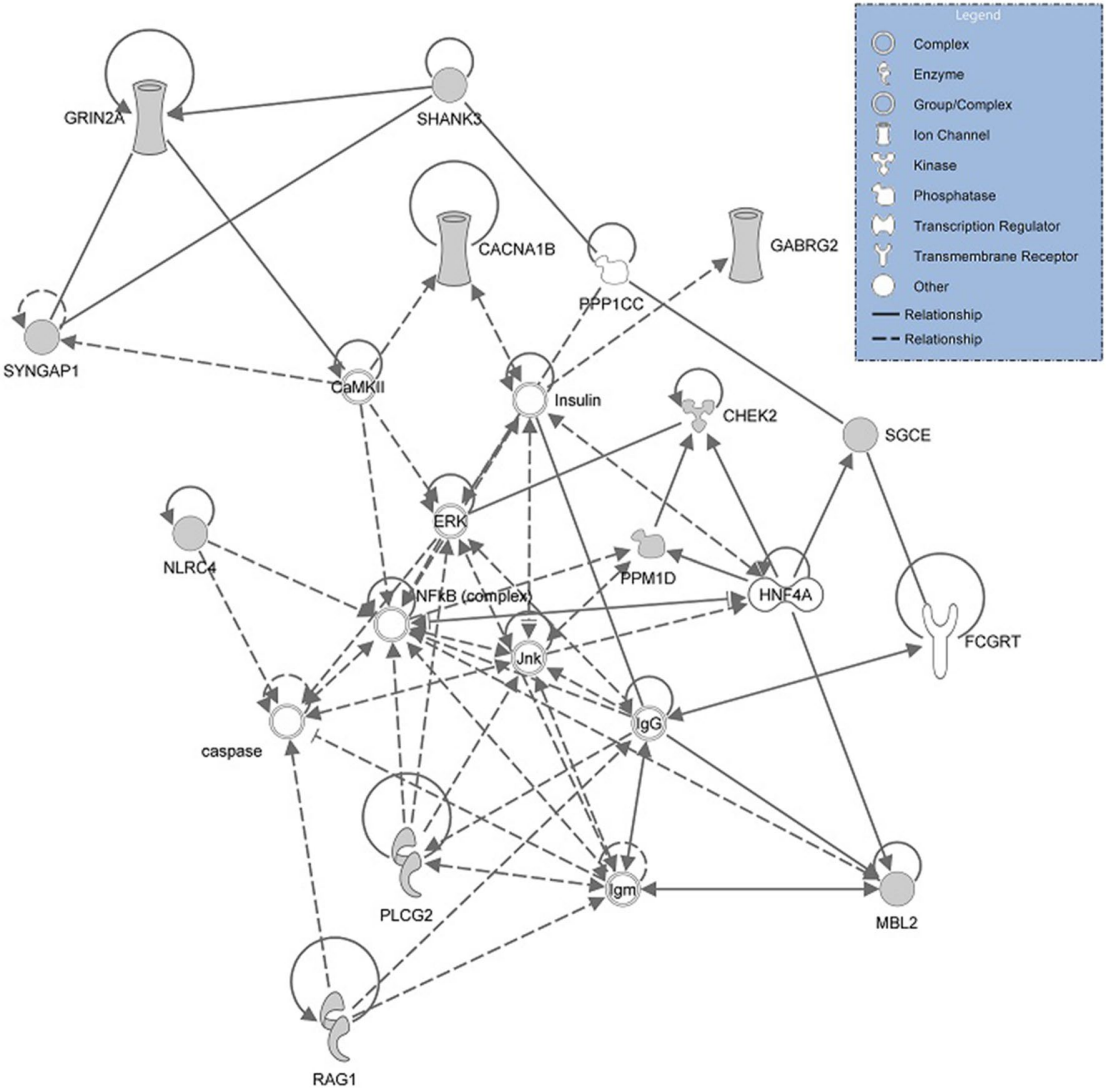
Connectivity network. A connectivity network was generated for each candidate gene using IPA software. Central to the network is the NF-κB complex transcriptional regulator hub, which connects to PPM1D, PLCG2, NLRC4, RAG1, BID, and CHK2. The PANS candidate genes not directly connected to NF-κB expression are those that are primarily expressed in the brain and cause neurodevelopmental disorders (CACNA1B, SYNGAP1, GRIN2A, SGCE, GABRG2, and SHANK3).

To assess potential functional connections between the PANS candidate genes, we generated a connectivity network to show the direct (solid lines) and indirect interactions (dotted lines) (Fig. 2). Central to the network is the NF-κB complex transcriptional regulator, which is activated by a variety of immune, infectious, and non-immune (e.g., oxidative stress; toxins) stressors^17,18^. Inappropriate activation of NF-κB has been associated with inflammatory diseases^19^. Directly connected to the NF-κB hub are the PANS candidate genes; *PPM1D*, *PLCG2, NLRC4, RAG1,* and *CHK2*. This hub represents the set of genes that likely function through a disruption of peripheral and central innate immunity. The PANS candidate genes not directly connected to NF-κB expression are those that are primarily expressed in the brain and cause neurodevelopmental disorders (*CACNA1B, SYNGAP1, GRIN2A, SGCE, GABRG2,* and *SHANK3*).

### Identification of PANS candidate genes with differential regulation in microglia

**Figure 3:**
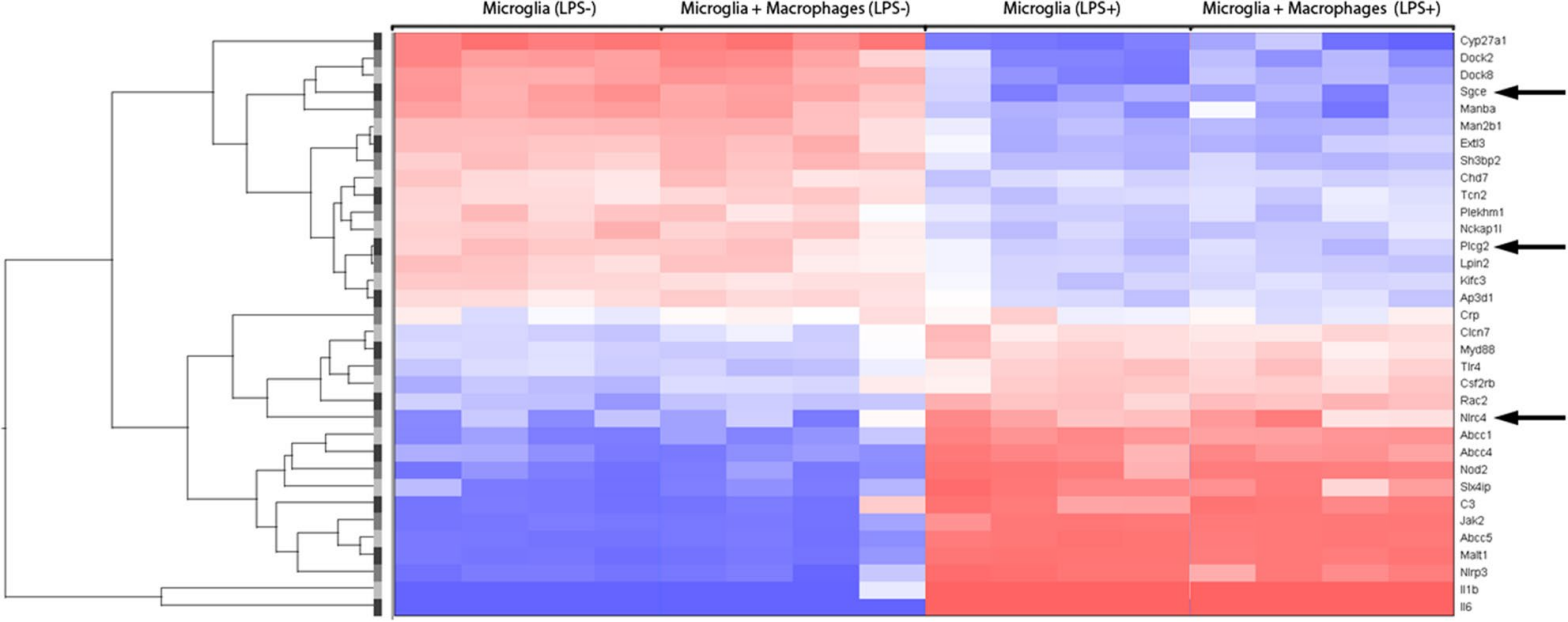
Heat map of LPS-mediated gene expression in microglia. Microarray gene expression data (GSE102482) from Greenhalgh et al. were analyzed to determine the expression pattern of a subset of 148 autism and pediatric immune disorder genes. The heat map shows the data obtained from RNA extracted from microglia grown on their own or co-cultured with peripheral macrophages. Arrows point to differentially expressed genes that are PANS candidates described in this report.

PPM1D, PLCG2, and NLRC4 have well-established effects on microglia function^20–22^. To further assess the expression of these genes and other PANS candidates in microglia, we analyzed a gene expression dataset derived from LPS-treated mice (GSE102482)^23^. We found that *Sgce* and *Plcg2* were the 2nd and 8th most downregulated transcripts, and *Nlrc4* the 6^th^ most upregulated transcript in microglia derived from control and LPS-treated mice upon an analysis of a subset of 148 autism and pediatric immune disorder genes (Fig. 3). Similarly, the expression of *Sgce* is induced in microglia following viral-mediated neuroinflammation in mice^24^, and we previously found that *Plcg2* expression increased significantly in microglia derived from a mouse model of Rett syndrome, in which innate immune pathways were the most enriched differentially expressed genes^25^.

### Expression of PANS candidate genes in PBMCs

To gain a more comprehensive understanding of the gene expression patterns in specific cell types and develop hypotheses for how PANS candidate gene variants lead to neuroinflammation, we examined several RNA expression resources, one of which was a single cell RNA sequence (scRNA-seq) database of peripheral blood mononuclear cells (PBMCs)^26^. This database contains scRNA-seq data from control samples (N = 6) and hospitalized COVID-19 patients (N = 7). The PANS candidate genes that have established effects on peripheral immune function, *PPM1D, CHK2,* and *RAG1,* were expressed in multiple PBMC types, while *PLCG2* was primarily expressed in B-cells, and *NLRC4* in monocytes and neutrophils (Supplementary Fig. S1). Interestingly, the expression of several of these genes was markedly altered in the COVID-19 samples. Most notably, a 170-fold increase in *NLRC4* expression and a 100-fold increase in *PLCG2* expression was found in developing neutrophils. In addition, 13 to 16-fold increases in *PPM1D* expression were found in IgG and IgM plasmablasts, interferon stimulated genes in T4 cells, and developing neutrophils in the COVID-19 samples, while a 12-fold decrease in expression in T.gd cells (γδ T cells) was observed. Finally, a 17-fold decrease in *CHK2* expression was found in interferon-stimulated genes in T4 cells derived from the COVID-19 patients.

Although most of the PANS candidate genes that are highly expressed in neurons were expressed at very low levels in PBMCs, we identified three notable exceptions. Most striking is *GABRG2,* which showed only marginal expression in all PBMCs, but a conspicuous, 24-fold increase in γδ T cells derived from the COVID-19 samples. *SYNGAP1* was expressed in many PBMCs, especially T cells. Like GABRG2, expression in γδ T cells increases approximately threefold in the COVID-19 samples. Finally, *SHANK3* expression, while negligible in most PBMCs, was abundantly expressed in a cluster consisting of two groups of cells labeled as stem cells and eosinophils. In addition, a 64% increase was detected in the COVID-19 samples.

### Expression of PANS candidate genes in the brain and other tissue

We next analyzed the cell and tissue expression pattern of the PANS candidate genes in normal human tissues using the GTEx dataset (Supplementary Fig. S2)^27^. Expression levels generally conformed to the functional groups described above and the connectivity network. The genes affecting peripheral immunity that are connected to the NF-κB hub were expressed primarily in blood and EB-transformed lymphoblasts, and less so in the brain, while the genes associated with neurodevelopmental disorders showed the opposite pattern. *PPM1D, RAG1,* and *SGCE* were highly expressed in both.

### Expression of PANS candidate genes in adolescent and fetal mouse brain

**Figure 4:**
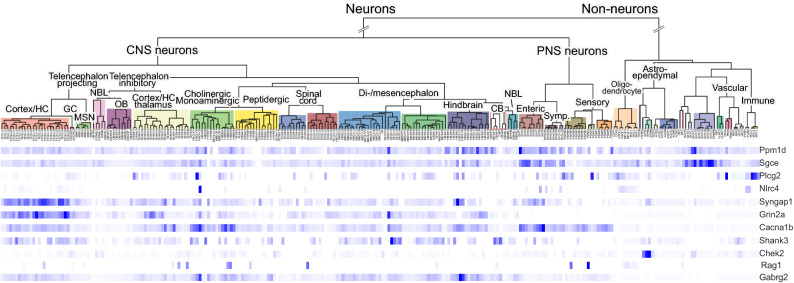
scRNA-seq expression pattern of each PANS candidate gene in mouse adolescent brains (open access article; CC BY license http://creativecommons.org/licenses/by/4.0/).

The GTEx dataset was developed from bulk RNA-seq. To further characterize the expression of PANS candidate genes in individual neuronal brain cell types, we used published scRNA-seq data from the mouse (adolescent and developing brains)^28,29^. One of the more striking observations in the adolescent brain is the relatively high level of *Chk2* expression in ependymal cells compared with all other brain cell types (Fig. 4; see Supplementary Table S1 for abbreviation key). Ependymal cells line the ventricles and spinal canal and play an important role in the production of CSF, and as part of the choroid plexus, regulation of the blood-CSF barrier^30^. The choroid plexus plays a key role in neuroinflammatory and neurodegenerative disorders^31–34^.

*Ppm1d, Sgce, Plcg2, Syngap1,* and *Shank3* were also expressed in these cells, suggesting that their mutated versions could disturb the blood-CSF barrier, allowing cytokines, chemokines, autoantibodies, and immune cells to enter the brain.

Another interesting finding in the mouse adolescent brain database was the relatively high level of *Ppm1d, Syngap1, Cacna1b,* and *Sgce* expression throughout the mouse enteric nervous system. The potential implications of this expression pattern will be described in the discussion section.

Expression In the developing mouse brain is shown in Supplementary Fig. S3. One of the more interesting findings was *Shank3,* which, as expected, was diffusely expressed throughout the brain in both glutamatergic neurons and GABAergic interneurons. However, the highest levels were seen in the choroid plexus and cerebral vasculature. In fact, all of the PANS candidate genes, with the exception of *RAG1* were expressed in fetal choroid plexus, cerebral vasculature, and pericytes, with *Sgce, Chk2,* and *Ppm1d,* along with *Shank3,* being most prominent. This was similar to the single cell expression pattern seen in adolescent brains (Fig. 4), reinforcing the idea that disruption of the blood-CSF barrier and/or the BBB is playing a role in the emergence of PANS.

## Discussion

In the current study, we used next generation sequencing to search for ultra-rare genetic variants in patients who met diagnostic criteria for PANS as established by the PANS Consensus Conference^1^. Mutations were identified in 21 patients in 11 genes that separate into two broad functional categories: those that affect peripheral innate and adaptive immune pathways, and those that are expressed primarily in cortical neurons, where they function as synaptic regulators and have all been implicated in other neurological and neurodevelopmental disorders. The findings additionally indicated that when mutated, the majority of genes could have unanticipated effects on immune cells, especially in response to infectious diseases. In addition, several genes are expressed in the choroid plexus and brain vasculature, suggesting they might contribute to a breach in the blood-CSF barrier and blood–brain barrier (BBB) that accompanies inflammation and infection. Although there is a clear ascertainment bias for the participants in the European sample, the finding of ultra-rare variants in the same genes and in an unselected modestly sized population in our United States cohort suggest that approximately 5% of PANS cases may be caused by biologically powerful ultra-rare genetic variants that affect immune and/or brain function.

Genetic contributions to disease can be divided into common variants that have relatively small biological effects, which are identified by large-scale GWAS, and high impact, biologically significant ultra-rare variants. So far, no reports have been published in which common variants associated with PANS have been identified, although, there is a small, unpublished study derived from a direct-to-consumer DNA testing service that reported several SNPs associated with PANS (https://ejournal2.undip.ac.id/index.php/jbtr/article/view/12082?utm_source=DSMN8&utm_medium=LinkedIn). However, the sample size is too small to assess their validity.

Several mutations were discovered in genes with well-established effects on peripheral innate and adaptive immunity: *NLRC4, PPM1D, RAG1, CHK2, and PLCG2. NLRC4* codes for a component of the inflammasome, a cytosolic multiprotein complex that assembles in response to exogenous or endogenous stressors that plays a major role in autoinflammatory diseases, macrophage activation syndrome, and panoptosis^35,36^. A total of four patients with ultra-rare variants in *NLRC4* were found in our two cohorts (cases 10, 13, 14, and 19; case 19 who also has an ultra-rare *SHANK3* variant). Two variants, c.772T>C (p.C258R) and c.928C>T (p.R310X) map to the NACHT domain, which has intrinsic ATPase activity and facilitates self-oligomerization^37^. Gain-of-function mutations in this region lead to severe hyperinflammatory disorders^38^. Such mutations abrogate autoinhibition by the C-terminal leucine rich region^39^. p.R310X would eliminate this region from the protein. The p.R310X variant has been observed in six patients with multiple sclerosis from two independent families following exome sequence screening, although the segregation pattern was incomplete, indicating that other factors may contribute to MS development in these families^40^.

The other two ultra-rare variants, c.2309 T>C (p.M770T) and c.1799A>G (p.E600G), map to the leucine-rich repeat region (LRR). Mutations in the LRR affecting the oligomerization interface were recently described in two patients with early-onset macrophage activation syndrome^41^.

Mutations in *PPM1D* were identified in 3 cases. *PPM1D* codes for a serine/threonine phosphatase that negatively regulates p53 and other members of the DNA repair pathway, and somatic gain-of-function mutations act as tumor suppressor genes^42^. The most common are frameshift and nonsense mutations in exons 5 and 6 that lead to the production of a truncated protein that has a stabilizing effect on the retained catalytic portion. Germline exons 5 and 6 truncating mutations are found in children with JdVS, which is characterized by intellectual disabilities (ID), restricted eating, high pain threshold, autism spectrum disorder (ASD), and psychiatric symptoms (primarily severe anxiety), symptoms that overlap with PANS^16^. Case 1 had severe, chronic PANS who was found, along with an affected sibling (Case 2), to have an ultra-rare PPM1D mutation. This mutation is upstream of the phosphatase domain and its effect on PPM1D catalytic function is not immediately clear. However, the nearby serine amino acids S40 and S46 are substrates for the serine kinases DYRK1A and HIPK2, which cause ID, ASD, and abnormal feeding behavior when mutated^43,44^. DYRK1A also has important effects on innate immunity by regulating the balance between T regulatory (Treg) cells and Th17 cells, the latter of which mediates the effects of *Streptococcus* on neuroinflammation in a mouse model of PANS^45–48^. HIPK2 regulates PPM1D protein levels and knockdown attenuates expression of inflammatory cytokines in LPS-stimulated macrophages^49,50^. The effect of S44W on immune function and its effect on DYRK1A and HIPK2 awaits experimental validation. The finding of PANS patient with a JdVS mutation (Case 3) strongly supports the idea that some *PPM1D* genetic variants increase the risk of PANS. It is interesting to note that most children with JdVS have a history of recurrent infections^16^. Although the underlying molecular and cellular basis for this is not known, infections and PANS could be due to the effect of PPM1D on T- and B-lymphocyte differentiation, and cytokine production, as described in humans and in mouse knockout models^51–54^.

The rare variant we identified in *CHK2*, is a truncating mutation in the distal end of the kinase domain and is probably a loss-of-function mutation. Like *PPM1D*, *CHK2* also codes for a regulator of the DNA repair response by phosphorylating p53^15,55^. It also regulates IL-2 expression in T-cells^56^, and Chk2 inactivation in mouse B cells leads to decreased Ig hypermutation, Ig class switching and immune dysregulation^57^.

*RAG1* codes for recombination activating gene 1, which is involved in antibody and T-cell receptor V(D)J recombination and loss of expression leads to abnormalities in T- and B-cell tolerance and immune dysregulation, leading to both an increased risk of infection and autoimmune problems^58,59^. Ultra-rare mutations were found in three patients (cases 15, 16, and 17), one of which causes a frameshift in exon 2 (c.256_257delAA; p.K86VfsX33). This variant has previously been reported in a heterozygous patient with adult-onset lymphopenia, and in a homozygous patient with severe combined immunodeficiency (SCID)^60^.

*PLCG2* is primarily expressed in B-cells. Gain-of-function mutations have previously been reported in patients with severe sterile inflammation, recurrent bacterial infections, autoimmune disorders, and humoral immunodeficiency^61^, and in autoinflammatory disease through an increase in calcium influx upon B-cell activation^62^. The *PLCG2* Y206F variant in Case 8 maps to the EF hand domain that binds calcium. However, the effect of Y206F on calcium homeostasis awaits experimental validation. Interestingly, this same ultra-rare variant was found in a patient diagnosed with Familial Cold Autoinflammatory Syndrome-3 (ncbi.nlm.nih.gov/clinvar/, accession number VCV000574390.1).

Six PANS candidate genes identified in this study, *SHANK3, GRIN2A, SYNGAP1, CACNA1B, GABRG2,* and *SGCE*, are primarily expressed in neurons, especially at excitatory synapses, and variants in these genes are associated with ASD and other neurodevelopmental disorders. *SHANK3,* for example, codes for a scaffold protein that regulates the assembly of the postsynaptic density (PSD) at glutamatergic excitatory synapses and is among the most commonly mutated genes in ASD and other neurodevelopmental disorders^63–65^. Of the 1003 genes in the Simons Foundation Autism Research Initiative (SFARI) database (gene.sfari.org/database/human-gene), *SHANK3* has the second highest number of reports. Ultra-rare variants were found in three PANS patients. Although *SHANK3* loss-of-function mutations in ASD are scattered throughout the gene, the majority, including our cases, are found in exon 21, which codes for the proline-rich region that binds to and attracts other synaptic proteins to the PSD.

Our finding of comorbid ASD and PANS in the three SHANK3 cases is similar to observations made by Bey et al., who identified de novo* SHANK3* variants in four girls with lifelong, stable developmental delay (DD) who developed subacute, severe psychiatric symptoms resembling PANS, that responded, with varying degrees of success, to immunotherapy^66^. Similarly, in an analysis of 38 individuals with Phelan-McDermid Syndrome, acute regressions triggered by infections, and other life events were common, and several patients improved with IVIg^67^. These findings show that a subgroup of patients with *SHANK3*-associated ASD and developmental disorders have an underlying susceptibility to develop acute onset neuropsychiatric problems that have a neuroinflammatory component.

In four of the other PANS-associated neuronal candidate genes we identified, *SYNGAP1, GRIN2A, GABRG2*, and *CACNA1B*, variants have been found in ASD, epilepsy, and ID^68–71^. According to the SFARI database, *SYNGAP1* has the ninth highest number of reports.

*GRIN2A* codes for the glutamate ionotropic receptor NMDA type subunit 2A. The ultra-rare variant found in Case 7 maps to a conserved region of the CTD domain, which interacts with the MAGUK (membrane-associated guanylate kinase) family of proteins that bind to PSD and function as important modulators of synaptic plasticity^72^. This subject has a history of dyslexia and ADHD.

*CACNA1B* codes for the voltage-dependent N-type calcium channel subunit alpha-1B, a constituent of the Cav2.2 channel. It too is a regulator of synaptic function that acts at the presynaptic terminal to increase neurotransmitter release, which then influences postsynaptic dendritic spine function^73^. In addition to having PANS, Case 4 was diagnosed with Hodgkin lymphoma that ultimately required an autologous hematological stem cell transplant (HSCT). It is interesting to note that Hodgkin lymphoma is often found in patients with autoimmune problems and is associated with abnormalities in IL-13 signaling, a cytokine produced by mast cells, eosinophils, nuocytes, and Th2 cells^74,75^. This patient also has psoriasis, a Th17 associated autoimmune disorder^76,77^. This suggests the possibility that *CACNA1B* could have unrecognized effects on the immune system by disrupting IL-13 and Th17 signaling leading to PANS and an increased risk of hematological malignancy. It is interesting to note that autologous HSCT is an emerging clinical tool for treating severe autoimmune disorders^78^, However, case 4 was in remission from PANS at the time HSCT was carried out.

GABRG2 codes for the gamma subunit of the GABA-A receptor, a ligand-gated chloride channel. It is one of the most mutated genes in febrile seizures and other forms of epilepsy^79^. We found a nonsense mutation in a child with severe PANS (Case 6) that responded to IVIg who also has a history of febrile seizures. This is the same mutation previously found in sleep-related epilepsy^80^.

Ultra-rare mutations in *SGCE* were found in two unrelated subjects, Cases 11 and 12. *SGCE* is a member of the sarcoglycan family of transmembrane proteins, a component of the dystrophin-associated glycoprotein complex (DGC). Case 11 responded to IVIg treatment. Studies in knockout mice show that SGCE disrupts excitatory synapse formation^81^. The DGC is involved in the formation of the glymphatic system, which is impaired in neuroinflammation and dementia^82,83^.

Remarkably, *SGCE, CACNA1B,* and *GRIN2A* are well-known for causing Myoclonus-Dystonia (M-D), a hyperkinetic movement disorder that resembles tics, which is found in a subgroup of PANS. *SGCE,* in fact, is the most commonly mutated gene in M-D, with more than one-third of cases having truncating mutations in exon 3, which contains the del150Iso in-frame mutation found in Case 11^84–86^. Interestingly, patients with myoclonus caused by *SGCE* mutations frequently have comorbid psychiatric symptoms, including depression, anxiety, bipolar disorder, and OCD^85^, symptoms that overlap with PANS. Similarly, *GRIN2A* and *CACNA1B* mutations have been found in dystonia and other movement disorders^87,88^.

These findings suggest that deleterious variants in these neuronal genes can result in a range of clinical phenotypes; M-D, ASD, developmental disabilities, epilepsy, and PANS, or PANS comorbid with these neurodevelopmental problems. It is important to add that cases 3 (*PPM1D*), 9 (*SYNGAP1*), 13 and 14 (*NLRC4*), 15 and 16 (*RAG1*), 19 and 20 (*SHANK3*), and 21 (*CHK2*) also had preexisting ASD or developmental delay prior to developing PANS.

Evidence that penetrance is not 100% is seen in several families in whom transmission occurred from a carrier parent who does not have PANS (Cases 1 and 2; 4 and 5), although other conditions such as asthma, allergies, and autoimmune disorders were reported. The lack of complete penetrance is consistent with a small monozygotic twin study in PANS that showed a range of different phenotypes, including complete discordance^89^**.** Similarly, the MZ twin concordance rate in autoimmune disorders ranges from approximately 20%-70% depending on the condition^90^. This suggests that environmental factors are playing a role in PANS. In the case of the generation divide between carrier parents and their affected children, changes in the prevalence of certain strains of bacteria and antigenic shifts over the decades in viruses implicated as PANS triggers could account for their different clinical outcomes. In addition, the current generation of youth might be exposed to non-infectious environmental triggers that were not as prevalent in past generations. Although unaffected carrier parents might be exposed to the same infectious agents or non-infectious stressors that are currently triggering PANS flareups in their children, age-related differences in the blood-CSF barrier or BBB could make the adult brain less susceptible^91^. Genetic background and stochastic T cell receptor and IgG gene rearrangement could also explain reduced penetrance in families and discordance in MZ twins.

The question remains if the PANS syndrome should be regarded as one or several clinical entities. The two functional groups into which our candidate genes can basically be divided; those that have established effects on peripheral innate and adaptive immunity, and those that affect synaptic function, in particular the PSD complex, indicates a peripheral versus central dichotomy. However, our results suggest this may be an oversimplification. First, as noted earlier, several PANS candidate genes that have effects on peripheral innate immunity, such as *PPM1D, PLCG2,* and *NLRC4,* also affect microglia function and are differentially expressed in those cells following an immune challenge (Fig. 3). Second, patients with ASD and mutations in genes that function as synaptic regulators, such as *SHANK3*, can respond to IVIg, as shown by Bey et al.^66^ and Case 20 in this report. Third, as presented in Supplementary Fig. S1, neuronal genes like *SHANK3, GABRG2* and *SYNGAP1* show expression patterns that suggest an effect on immune function, in particular γδ T-cells, based on their markedly altered expression during an infectious disease challenge. γδ T cells form a minor population of PBMCs, but they increase in number dramatically during infections and play a key role in autoimmunity and immune surveillance^92^. γδ T cells are also found in meninges where they secrete the proinflammatory cytokine IL-17a and participate in the development of anxiety-like behavior in mice^93^. Fourth, the neuronal PANS candidate genes that have effects on synaptic function are also expressed in the choroid plexus and brain vascular endothelium, in particular *SHANK3,* which could potentially connect peripheral inflammation with neuroinflammation through disruption of the brain/CSF barrier and BBB. SHANK3 is a cytoskeletal protein that regulates glutamatergic synaptogenesis, but it can also function as a scaffolding protein in epithelial cells^94^. In addition, PPM1D has been found to regulate BBB function and neuroinflammation in a co-culture of human brain-microvascular endothelial cells and human astrocytes treated with LPS^95^.

Expression of PANS candidate genes in the choroid plexus and vascular endothelium during fetal development is also interesting when considering the phenomenon known as maternal immune activation (MIA), a proinflammatory state in pregnancy triggered by infection, maternal autoimmune disorders, and non-infectious peripheral inflammation^96^. The fetal brain is vulnerable to changes in the maternal/fetal environment, such as MIA, which has been shown in animal models to adversely affect brain development, leading to behaviors and learning difficulties similar to those seen in ASD and schizophrenia^97,98^. Consequently, it is conceivable that the pathophysiological process that leads to the development of PANS and comorbid neurodevelopmental disorders could begin during fetal life in some patients and genetic subgroups. An effect on the choroid plexus and vasculature endothelium (and γδ T cells) could also explain the response to IVIg in those patients harboring mutations in the “neuronal” subgroup of candidate genes. IVIg can reduce neuroinflammation by affecting T-cell/microglia crosstalk, reducing levels of proinflammatory cytokines, blocking Fcγ receptors, inhibiting complement, and repairing disrupted brain-CSF barriers^99,100^.

It is also interesting to consider the implications of the finding that several PANS candidate genes are also mutated in D-M, ASD, and other neurodevelopmental disorders, as noted above. PANS is occurring as an independent phenotype in some of our cases, and as a comorbid trait in others (Table 2). These comorbid cases are like the *SHANK3-*mutated patients reported by Bey et al.^66^. Similarly, Jones et al., described eight children with ASD and other neurodevelopmental disorders, with a strong family history of maternal autoimmune thyroid disorders, who presented with infection-induced, abrupt onset of neuropsychiatric symptoms, primarily OCD and tics, along with autistic or global regression^101^. Numerous genetic and molecular studies also indicate an inflammatory component in the pathogenesis of subgroups of patients with schizophrenia and ASD^102–105^. In addition, it has been suggested that a subgroup of regressive autism, which is characterized by the sudden loss of previously acquired traits in early childhood, could have an autoimmune or infectious disease etiology^106–108^. Interestingly, deleterious *GRIN2A* mutations were found in ~ 2% of cases in a recent study by Yin et al.^107^. At the very least, an inflammatory component leading to the abrupt onset of PANS superimposed on a chronic neurodevelopmental disorder appears to be a real phenomenon and tools to improve early recognition of these cases may be used to motivate immunological therapies like IVIg that empirical evidence suggests could be very helpful.

Finally, an interesting aspect of our scRNA-seq analysis was the finding that many of the PANS candidate genes are expressed in enteric neurons. Enteric neurons have many connections with immune cells in the intestinal mucosa, and gut-associated lymphoid tissue, which could affect, or be affected by gut bacteria^109^. More specific to the candidate genes we identified, several have established effects on the gut. PPM1D, for example, has been found to have a protective effect on oxidation stress-induced gut permeability^110^. In addition, patients with PANS have been found to have differences in the gut microbiome compared with controls^111^, and have an increase in gut derived LPS^112^. In addition, *Shank3* knockout mice have an altered microbiota composition, an increase in LPS levels in the liver, and altered gut permeability^113^. Finally, mutations in *NLRC4* have been implicated in inflammatory bowel disease^114,115^. Thus, expression of PANS candidate genes in the enteric nervous system fits into the emerging idea that disruption of the gut-brain connection and the gut microbiome are involved in the pathogenesis of ASD, neurodegeneration, neuropsychiatric disorders, autoimmune disorders, and PANS.

In conclusion, we identified ultra-rare genetic variants in PANS patients that appear to function at multiple levels of the neuroinflammatory circuit, including peripheral and central innate immunity, synaptogenesis, the blood-CSF barrier, and perhaps the enteric nervous system. Dissecting the molecular and cellular pathogenesis of the PANS candidate variants will require an analysis in mouse models, as well as patient-specific induced pluripotent stem cells, from which cells such as neurons, microglia, astrocytes, vascular endothelium, and gut organoids can be derived to assess their importance in the development of the devastating symptoms that comprise the PANS syndrome.

## Subjects and methods

### Subjects

The European subjects were identified through a call for patients with severe symptoms from a PANS group called EXPAND, a European advocacy organization for families of children and adolescents with immune-mediated neuropsychiatric disorders, after a teenager with chronic PANS was found to have an ultra-rare variant in the *PPM1D* gene by WGS. Parents, caretakers, or patients signed informed consents approved by the Ethical Committee at Erasmus MC (MEC-2011–253 for control samples and MEC-2021–0359 for PANS patients; IRB/ Human Subject Assurance number/Federal Wide Assurance, FWA00001336) in accordance with the Declaration of Helsinki – ethical principles for medical research involving human subjects. Histories from this cohort were obtained by the participating physicians and collated by one of the co-authors (O.M). Cases from the U.S. were obtained from a large private practice run by one of the authors (R.T) devoted to PANS (PANDAS/PANS Institute, Ramsey, NJ). 383 PANS cases and 263 controls that included 133 triads had WES carried out by Centogene (see below) (Rostock, Germany). The subjects signed an informed consent with Centogene. Each case in the U.S. cohort was personally treated by one physician (R.T.) who obtained a detailed personal and family history, and permission to present genetic and clinical information relevant to the diagnosis of PANS. Each case met diagnostic criteria for PANS as established by the PANS Consensus Conference^1^. For cases and family members diagnosed with autoimmune disorders, no formal assessments were carried out for this study for most of the subjects. We relied on detailed family histories obtained by the personal physicians.

### WGS and WES data analysis

The EU cases (1, 2, 4–11) were sequenced using DAB nanoball sequencing for WGS and Illumina for WES. The variants shown in Table 2 were found in both sequencing efforts. We implemented ANNOVAR, an efficient software tool that utilizes up-to-date information to functionally annotate genetic variants and allele frequencies detected in each patient’s genome. In addition to gene-based annotation, ANNOVAR is used for region-based annotations to identify variants in specific genomic regions, such as ultra-conserved regions across species (Vista database), predicted transcription factor binding sites (Transfac), segmental duplication regions, genome wide association study (GWAS) hits, database of genomic variants, DNAse I hypersensitivity sites, ENCODE H3K4Me1/H3K4Me3/H3K27Ac/CTCF sites, ChIP-Seq peaks, RNA-Seq peaks, and other annotations in genomic intervals. DNA nanoball sequencing was applied for WGS to study non-coding regions and in particular variants within the ultra-conserved regulatory regions. This provided highly reliable variant lists that were compared with our selected variants and filtered based on allele frequency in GNOMAD ClinVar (public NCBI repository derived data). Only variants that occurred in patients and were never detected in 597 healthy elderly (Wellderly) sample genomes were considered^116^. DNA Nanoball sequencing protocol is detailed in the classic paper by Drmanac et al.^117^. The ultra-rare variant assessment was confirmed using additional cohorts that have been sequenced by the Erasmus MC team headed by one of us (P.S.).

The U.S. samples were sequenced by Centogene using double stranded DNA capture baits against approximately 36.5 Mb of the human coding exome (targeting > 98% of the coding elements). RefSeq from the human genome build GRCh37/hg19) are used to enrich target regions from fragmented genomic DNA with the Twist Human Core Exome Plus kit. The generated library was sequenced on an Illumina platform to obtain at least 20 × coverage depth for > 98% of the targeted bases. The investigation for relevant variants focused on coding exons and flanking ± 20 intronic nucleotides of genes with clear gene-phenotype evidence. The resulting variant call file (VCF) file was then subjected to a custom pipeline developed by one of the co-authors (R.T). Variants were filtered as follows: 1. Those with annotated mean allele frequencies of > 0.1% were removed, 2. Variants marked as "benign" or "likely benign" on EITHER ClinVar or ACMG/InterVar database were removed, 3. Variants annotated as “low severity” were removed.

### Network analysis

Functional analysis was performed within Ingenuity Pathway Analysis (IPA) (QIAGEN Inc.). All candidate genes were imported in IPA to assess the pathways involved. Using the network tool within IPA, a connectivity network was constructed based on the IPA/QIAGEN Knowledge Base. Both direct and indirect relationships were used to construct the biological network.

### RNA expression analysis

The gene expression profiles of the candidate genes were analyzed using several datasets. First, we hypothesized that microglia might play a critical role in the PANS phenotype especially after an inflammatory stimulus. Consequently, a subset of 159 autism and pediatric immune disorder genes was analyzed based on a public dataset investigating the effect of lipopolysaccharide (LPS) on murine microglia and macrophages (GSE102482)^23^. After Robust Multichip Average normalization (RMA), a statistical analysis of microarray (SAM) was performed between non-stimulated microglia and LPS-stimulated microglia within OmniViz version 6.1.13.0 (Instem Scientific). The top 15 up-regulated and down-regulated genes were selected for visualization and further analysis.

Next, we investigated the gene expression profiles of our 11 PANS-associated candidates in peripheral blood mononuclear cells (PBMCs) under baseline conditions and after infection as many patients develop PANS symptoms following infections. Given the rising reporting of neuropsychiatric symptoms after COVID-19 infection, we used a recently published sc-RNA-seq COVID-19 dataset to investigate the gene expression profiles^26^. This database contains gene expression patterns in control samples (N = 6) and hospitalized severe COVID-19 patients (N = 7).

To investigate the expression of the candidate genes in normal human tissue we used the Genotype-Tissue Expression (GTEx) dataset (https://www.gtexportal.org/home)^27^. The Multi Gene Query available on the GTEx portal was used to construct the RNA profiles in normal human tissue lineages. Finally, as the GTEx portal uses bulk RNA-seq data, we wanted to further investigate the gene expression profiles in brain tissue using scRNA-seq. Since scRNA-seq data is not yet available for human brains, we analyzed the expression pattern in two datasets that were recently published containing scRNA-seq data for adolescent and fetal mouse brains^28,29^. Each candidate gene was investigated in both datasets on the Mouse Brain Atlas portal (http://mousebrain.org).

## Supplementary Information

Supplementary Information.
