# Supplementary material for: Identification of ultra-rare genetic variants in pediatric acute onset neuropsychiatric syndrome (PANS) by exome and whole genome sequencing
Source: Sci Rep. 2022 Jun 30;12:11106. doi: 10.1038/s41598-022-15279-3 (PMC9246359; doi:10.1038/s41598-022-15279-3)
Supplement: Supplementary file 1 — Supplementary Information. [file 41598_2022_15279_MOESM1_ESM.pdf]

Supplementary Information

Supplementary Figure 1: Single cell RNA-seq (scRNA-seq) peripheral blood cells. The expression levels of each candidate gene was analyzed using a scRNA-seq database of PBMCs in controls and hospitalized Covid-19 patients (reference 26.) (http://rstats.immgen.org/Skyline_COVID-19/skyline.html19/skyline.html). Permission: https://www.nature.com/nature-portfolio/reprints-and-permissions

Supplementary Figure 2: Expression pattern of PANS candidate genes in human tissue from GTEx database. (https://www.gtexportal.org/home)

Supplementary Figure 3: Wheel plots showing single cell expression data from the developing mouse brain (http://mousebrain.org). The expression pattern is displayed as a UMAP clusters of2 different cell types determined by the expression pattern of cell specific markers. Slides 2 and 3 show the different cell types making up the clusters, while slide 4 shows the brain regions covered by the clusters. Slide 5 shows an example of a gene that is expressed in a cell type-specific manner, which was used to create the gene clusters. Slides 6-16 show the expression pattern at a common developmental stage. The box on the left of each wheel plot is the relative expression level within the different cell types.

Supplementary Table: Abbreviation key to Figure 4 (mouse adolescent brain scRNA-seq)

Availability of Data and Materials: The datasets used and/or analyzed during the current study available from the corresponding author on reasonable request. We kindly ask you to reach out by Email to p.vanderspek@erasmusmc.nl (European Union samples), or trifmd@gmail.com (United States Samples). All requests that are in line with GDPR will be granted

Supplementary Figure S1: cell RNA-seq (scRNA-seq) peripheral white blood cells

http://rstats.immgen.org/Skyline_COVID-19/skyline.html

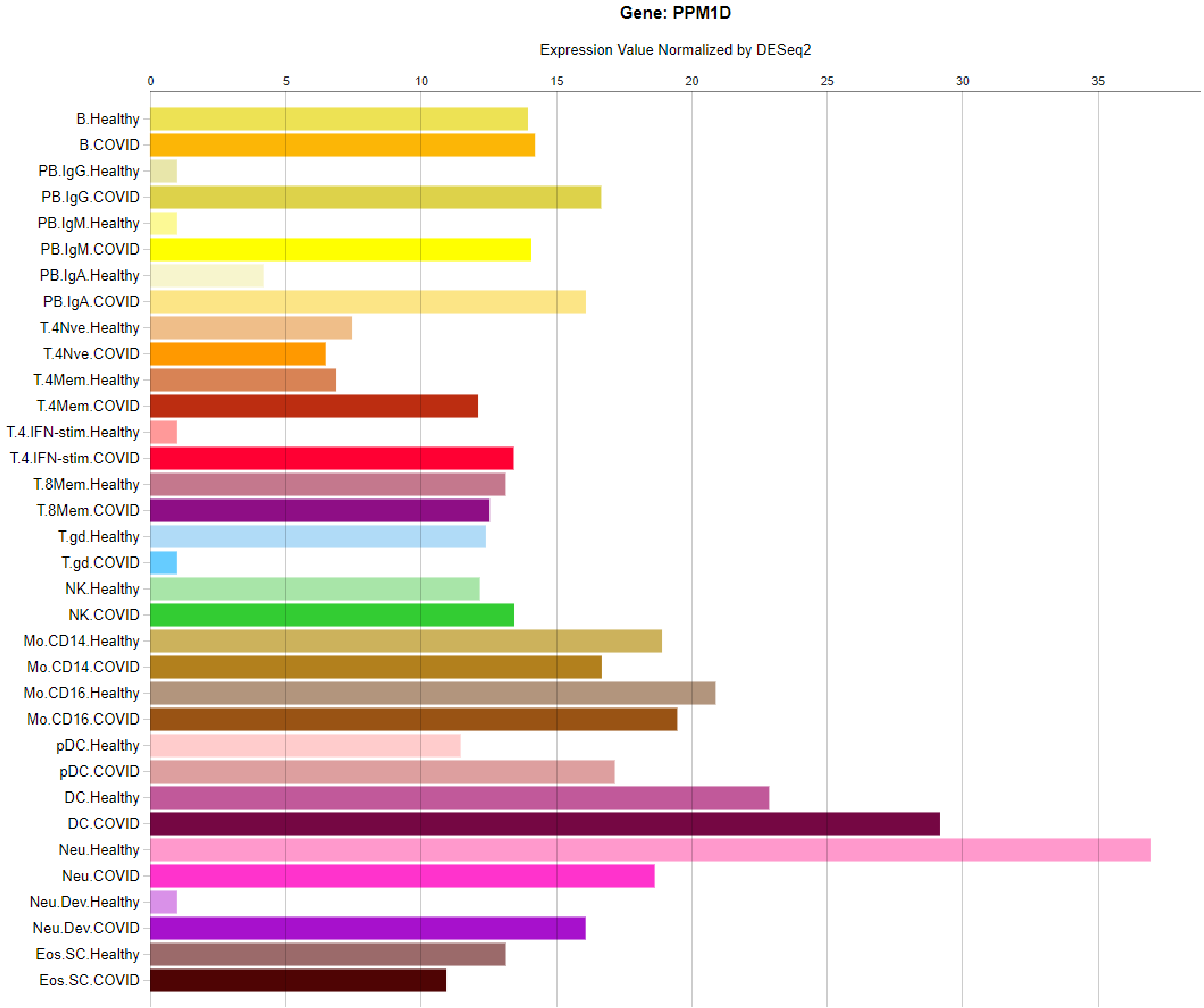

Gene: PPM1D
Expression Value Normalized by DESeq2
0
5
10
15
20
25
30
35
B.Healthy
B.COVID
PB.IgG.Healthy
PB.IgG.COVID
PB.IgM.Healthy
PB.IgM.COVID
PB.IgA.Healthy
PB.IgA.COVID
T.4Nve.Healthy
T.4Nve.COVID
T.4Mem.Healthy
T.4Mem.COVID
T.4.IFN-stim.Healthy
T.4.IFN-stim.COVID
T.8Mem.Healthy
T.8Mem.COVID
T.gd.Healthy
T.gd.COVID
NK.Healthy
NK.COVID
Mo.CD14.Healthy
Mo.CD14.COVID
Mo.CD16.Healthy
Mo.CD16.COVID
pDC.Healthy
pDC.COVID
DC.Healthy
DC.COVID
Neu.Healthy
Neu.COVID
Neu.Dev.Healthy
Neu.Dev.COVID
Eos.SC.Healthy
Eos.SC.COVID

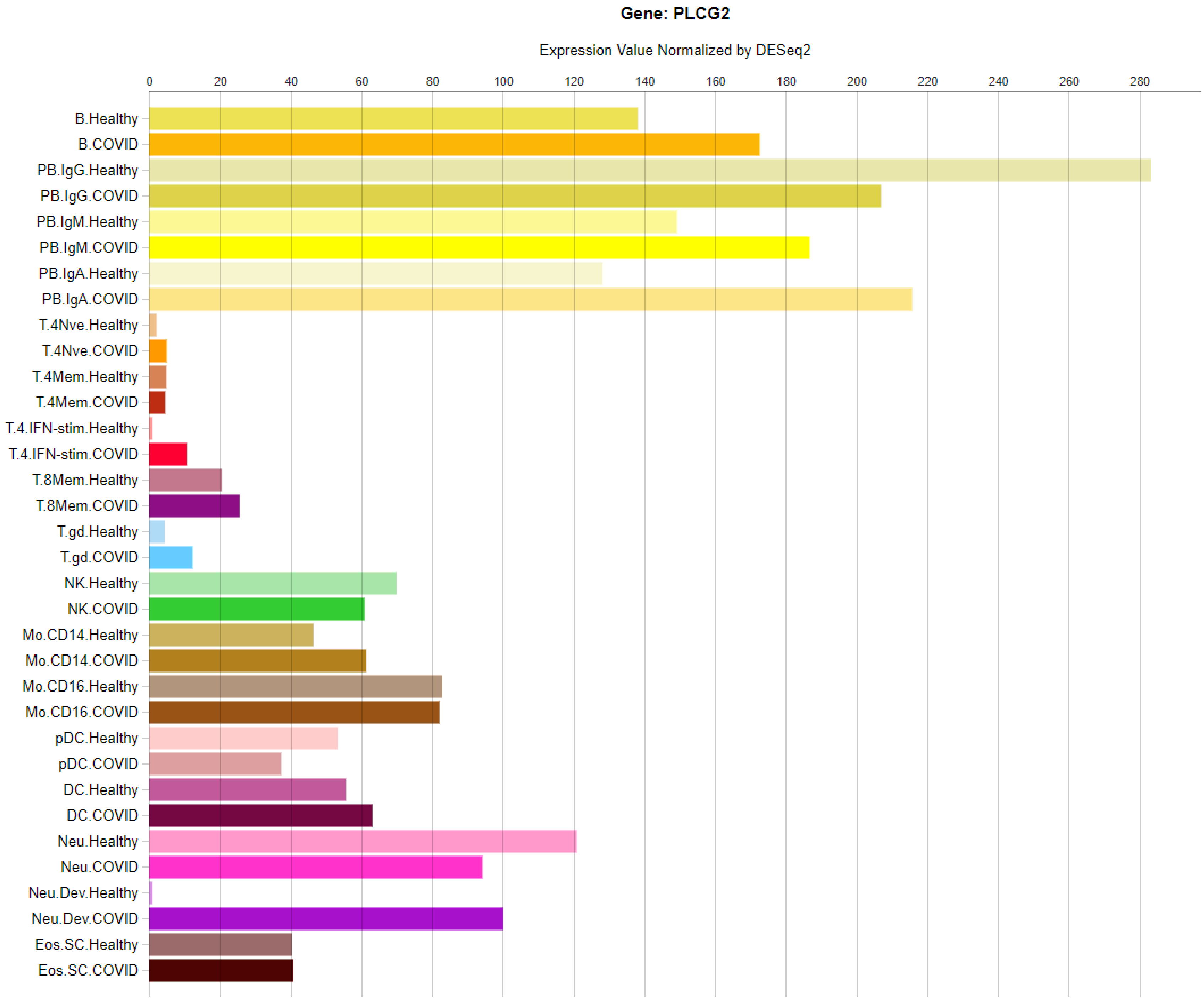
Gene: PLCG2
Expression Value Normalized by DESeq2
0
20
40
60
80
100
120
140
160
180
200
220
240
260
280
B.Healthy
B.COVID
PB.IgG.Healthy
PB.IgG.COVID
PB.IgM.Healthy
PB.IgM.COVID
PB.IgA.Healthy
PB.IgA.COVID
T.4Nve.Healthy
T.4Nve.COVID
T.4Mem.Healthy
T.4Mem.COVID
T.4.IFN-stim.Healthy
T.4.IFN-stim.COVID
T.8Mem.Healthy
T.8Mem.COVID
T.gd.Healthy
T.gd.COVID
NK.Healthy
NK.COVID
Mo.CD14.Healthy
Mo.CD14.COVID
Mo.CD16.Healthy
Mo.CD16.COVID
pDC.Healthy
pDC.COVID
DC.Healthy
DC.COVID
Neu.Healthy
Neu.COVID
Neu.Dev.Healthy
Neu.Dev.COVID
Eos.SC.Healthy
Eos.SC.COVID

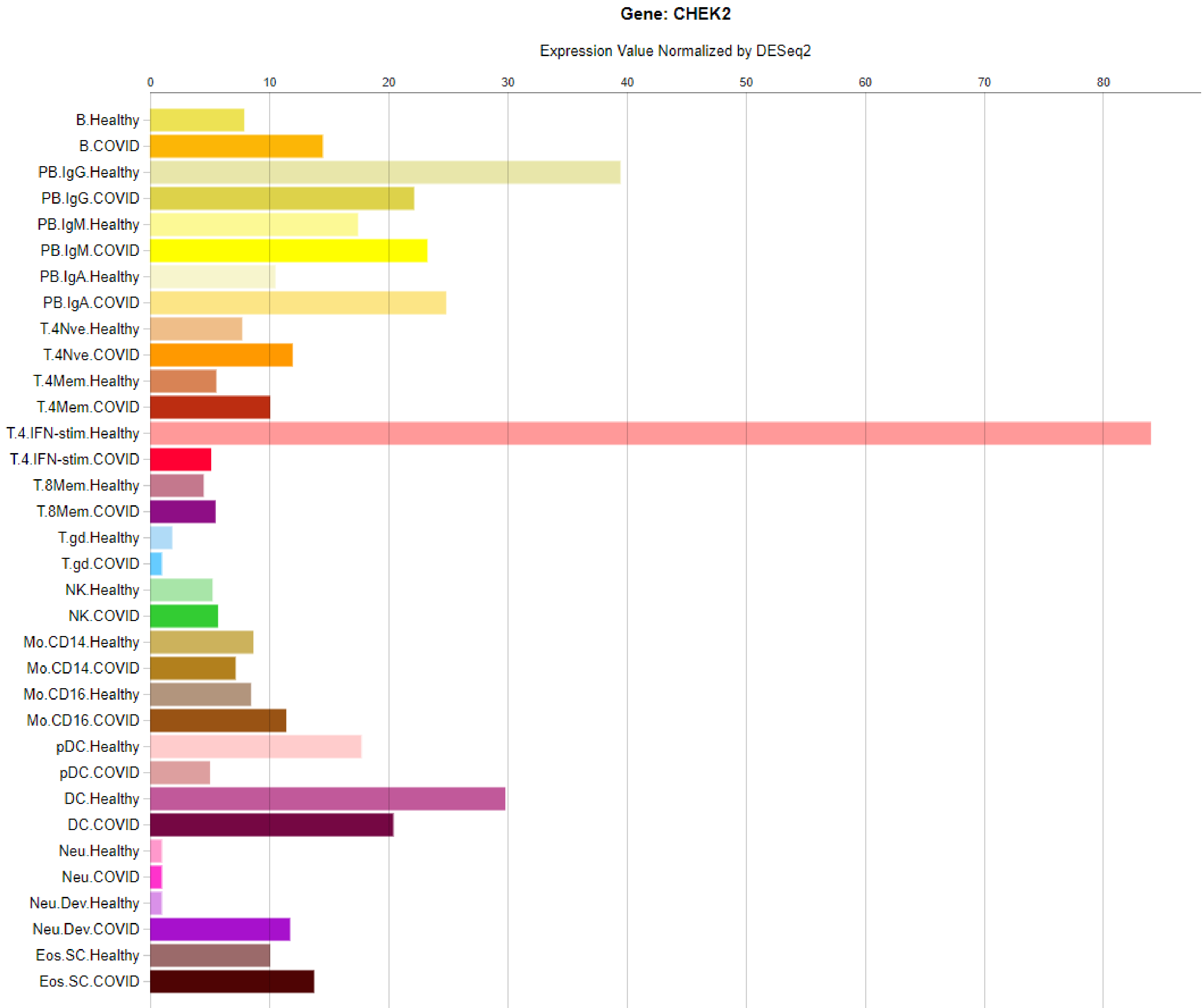

Gene: CHEK2
Expression Value Normalized by DESeq2
0
10
20
30
40
50
60
70
80
B.Healthy
B.COVID
PB.IgG.Healthy
PB.IgG.COVID
PB.IgM.Healthy
PB.IgM.COVID
PB.IgA.Healthy
PB.IgA.COVID
T.4Nve.Healthy
T.4Nve.COVID
T.4Mem.Healthy
T.4Mem.COVID
T.4.IFN-stim.Healthy
T.4.IFN-stim.COVID
T.8Mem.Healthy
T.8Mem.COVID
T.gd.Healthy
T.gd.COVID
NK.Healthy
NK.COVID
Mo.CD14.Healthy
Mo.CD14.COVID
Mo.CD16.Healthy
Mo.CD16.COVID
pDC.Healthy
pDC.COVID
DC.Healthy
DC.COVID
Neu.Healthy
Neu.COVID
Neu.Dev.Healthy
Neu.Dev.COVID
Eos.SC.Healthy
Eos.SC.COVID

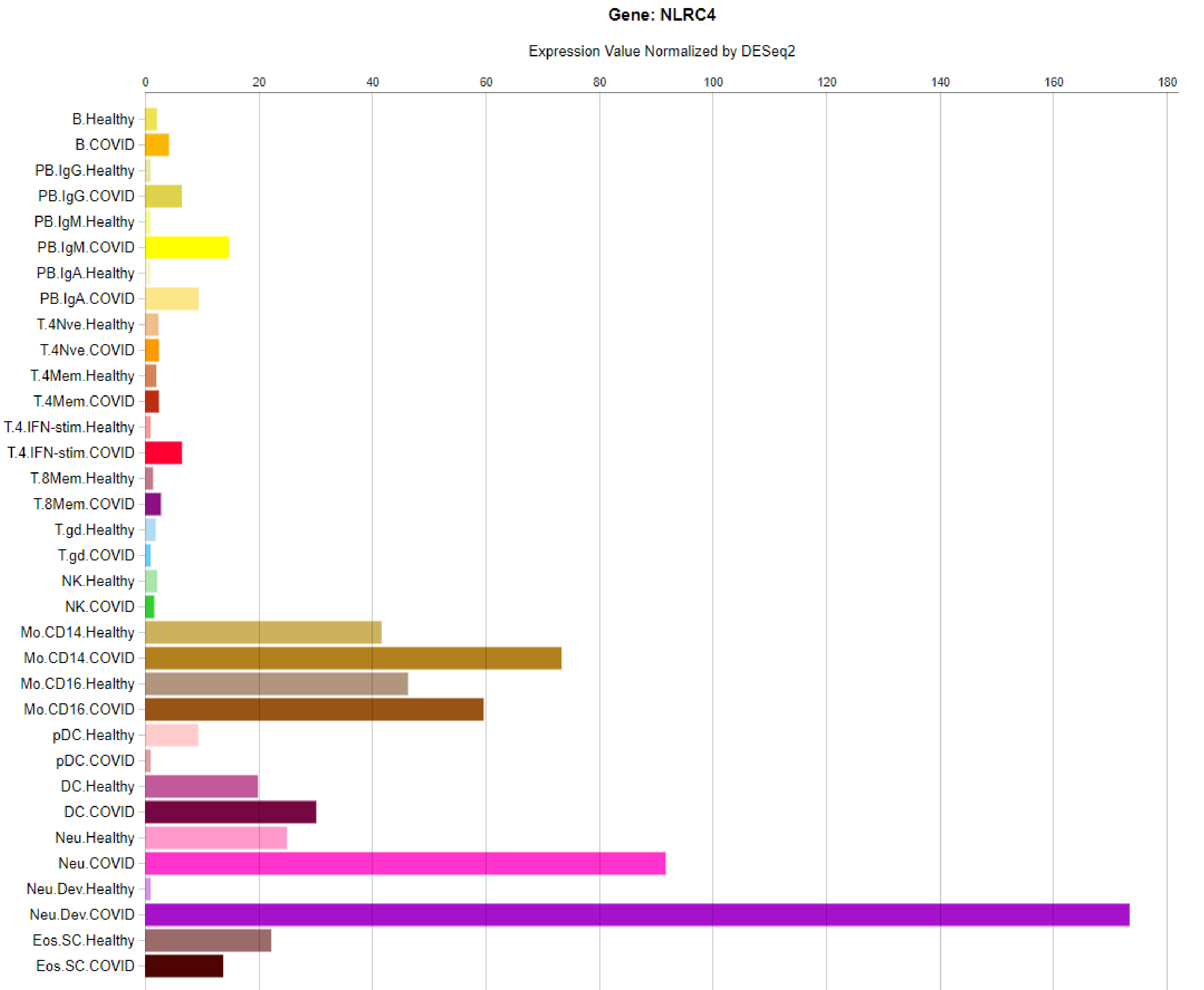
Gene: NLRC4
Expression Value Normalized by DESeq2
0
20
40
60
80
100
120
140
160
180
B.Healthy
B.COVID
PB.IgG.Healthy
PB.IgG.COVID
PB.IgM.Healthy
PB.IgM.COVID
PB.IgA.Healthy
PB.IgA.COVID
T.4Nve.Healthy
T.4Nve.COVID
T.4Mem.Healthy
T.4Mem.COVID
T.4.IFN-stim.Healthy
T.4.IFN-stim.COVID
T.8Mem.Healthy
T.8Mem.COVID
T.gd.Healthy
T.gd.COVID
NK.Healthy
NK.COVID
Mo.CD14.Healthy
Mo.CD14.COVID
Mo.CD16.Healthy
Mo.CD16.COVID
pDC.Healthy
pDC.COVID
DC.Healthy
DC.COVID
Neu.Healthy
Neu.COVID
Neu.Dev.Healthy
Neu.Dev.COVID
Eos.SC.Healthy
Eos.SC.COVID

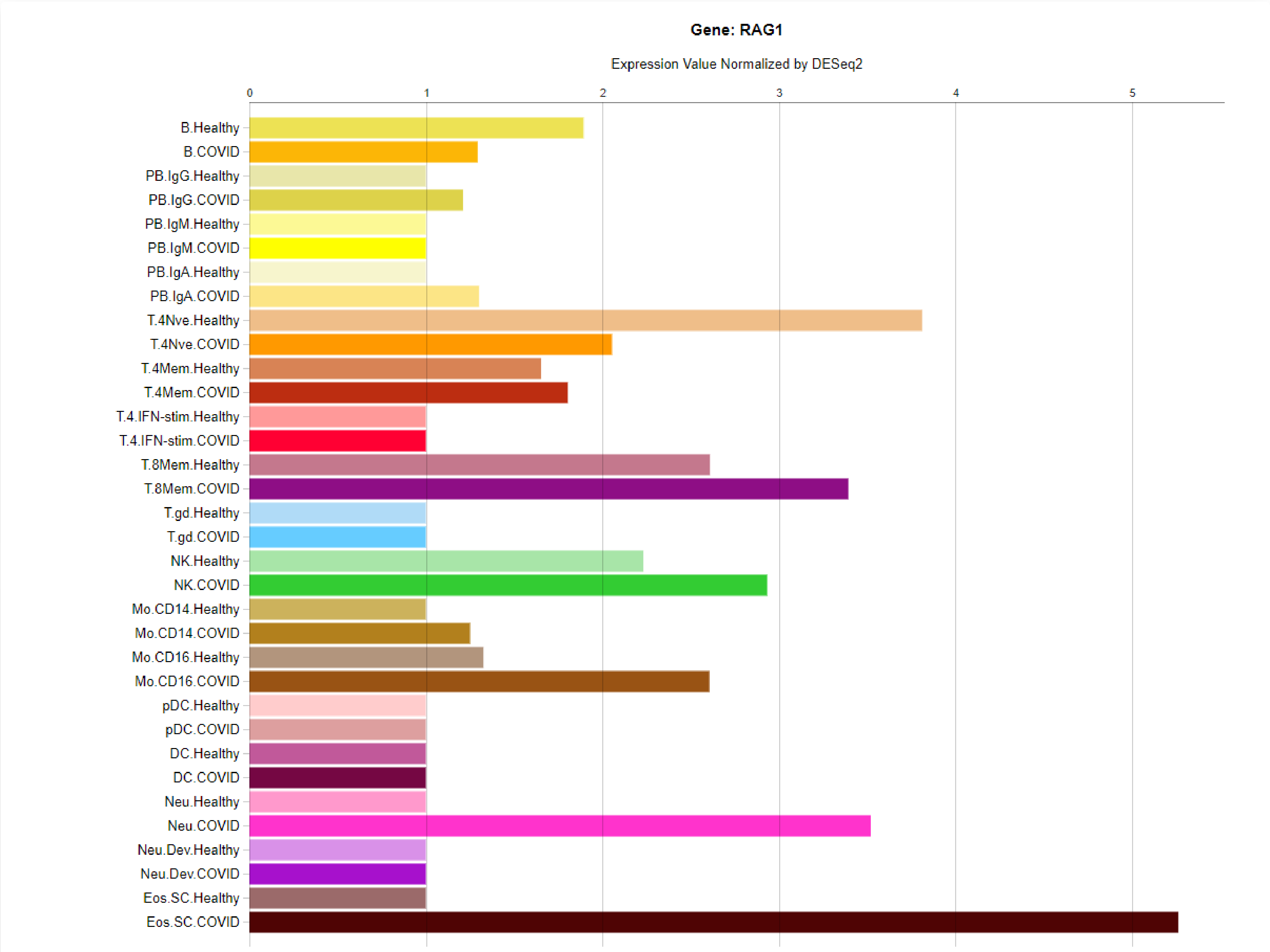
Gene: RAG1
Expression Value Normalized by DESeq2
0
1
2
3
4
5
B.Healthy
B.COVID
PB.IgG.Healthy
PB.IgG.COVID
PB.IgM.Healthy
PB.IgM.COVID
PB.IgA.Healthy
PB.IgA.COVID
T.4Nve.Healthy
T.4Nve.COVID
T.4Mem.Healthy
T.4Mem.COVID
T.4.IFN-stim.Healthy
T.4.IFN-stim.COVID
T.8Mem.Healthy
T.8Mem.COVID
T.gd.Healthy
T.gd.COVID
NK.Healthy
NK.COVID
Mo.CD14.Healthy
Mo.CD14.COVID
Mo.CD16.Healthy
Mo.CD16.COVID
pDC.Healthy
pDC.COVID
DC.Healthy
DC.COVID
Neu.Healthy
Neu.COVID
Neu.Dev.Healthy
Neu.Dev.COVID
Eos.SC.Healthy
Eos.SC.COVID

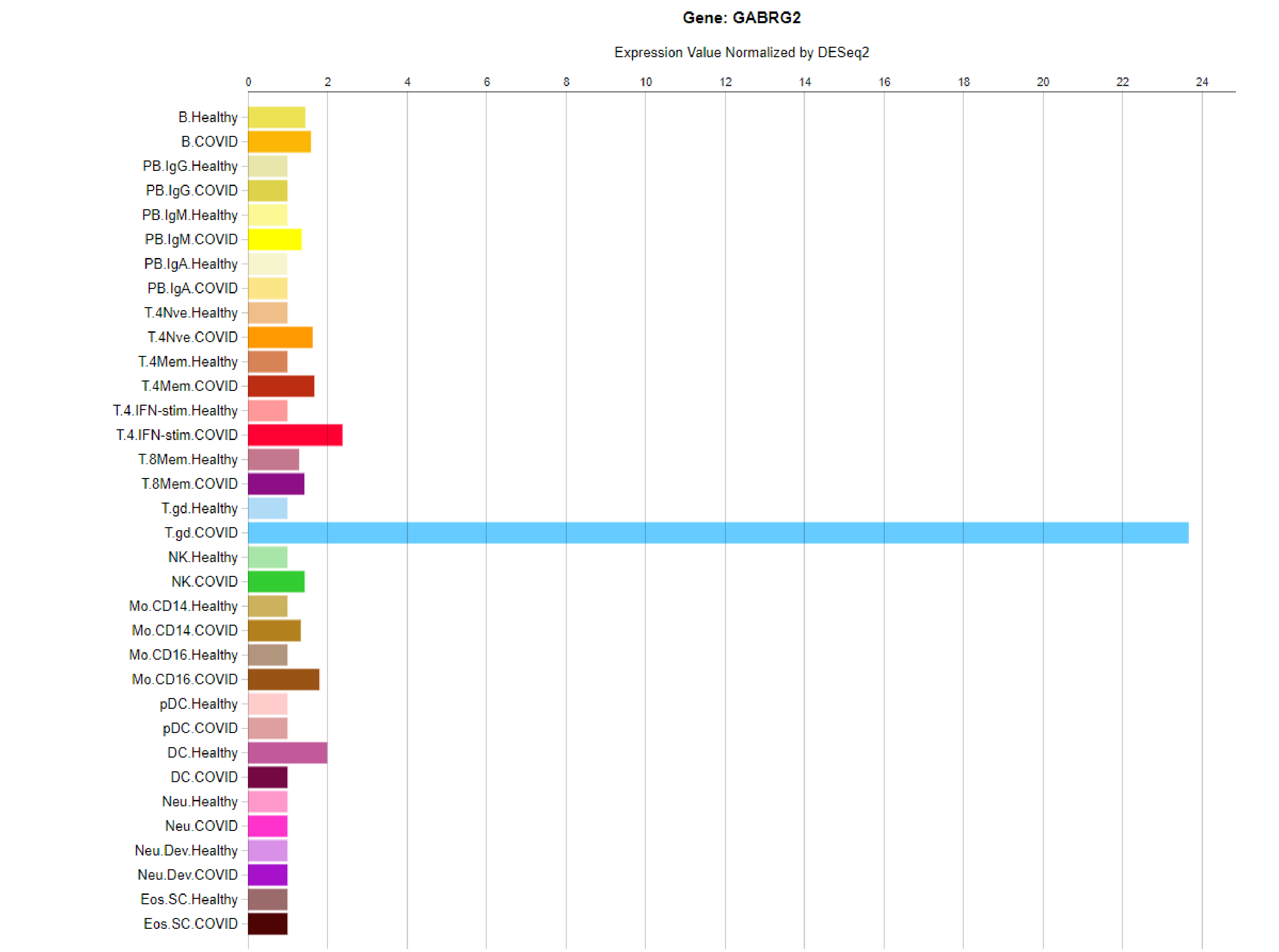
Gene: GABRG2
Expression Value Normalized by DESeq2
0
2
4
6
8
10
12
14
16
18
20
22
24
B.Healthy
B.COVID
PB.IgG.Healthy
PB.IgG.COVID
PB.IgM.Healthy
PB.IgM.COVID
PB.IgA.Healthy
PB.IgA.COVID
T.4Nve.Healthy
T.4Nve.COVID
T.4Mem.Healthy
T.4Mem.COVID
T.4.IFN-stim.Healthy
T.4.IFN-stim.COVID
T.8Mem.Healthy
T.8Mem.COVID
T.gd.Healthy
T.gd.COVID
NK.Healthy
NK.COVID
Mo.CD14.Healthy
Mo.CD14.COVID
Mo.CD16.Healthy
Mo.CD16.COVID
pDC.Healthy
pDC.COVID
DC.Healthy
DC.COVID
Neu.Healthy
Neu.COVID
Neu.Dev.Healthy
Neu.Dev.COVID
Eos.SC.Healthy
Eos.SC.COVID

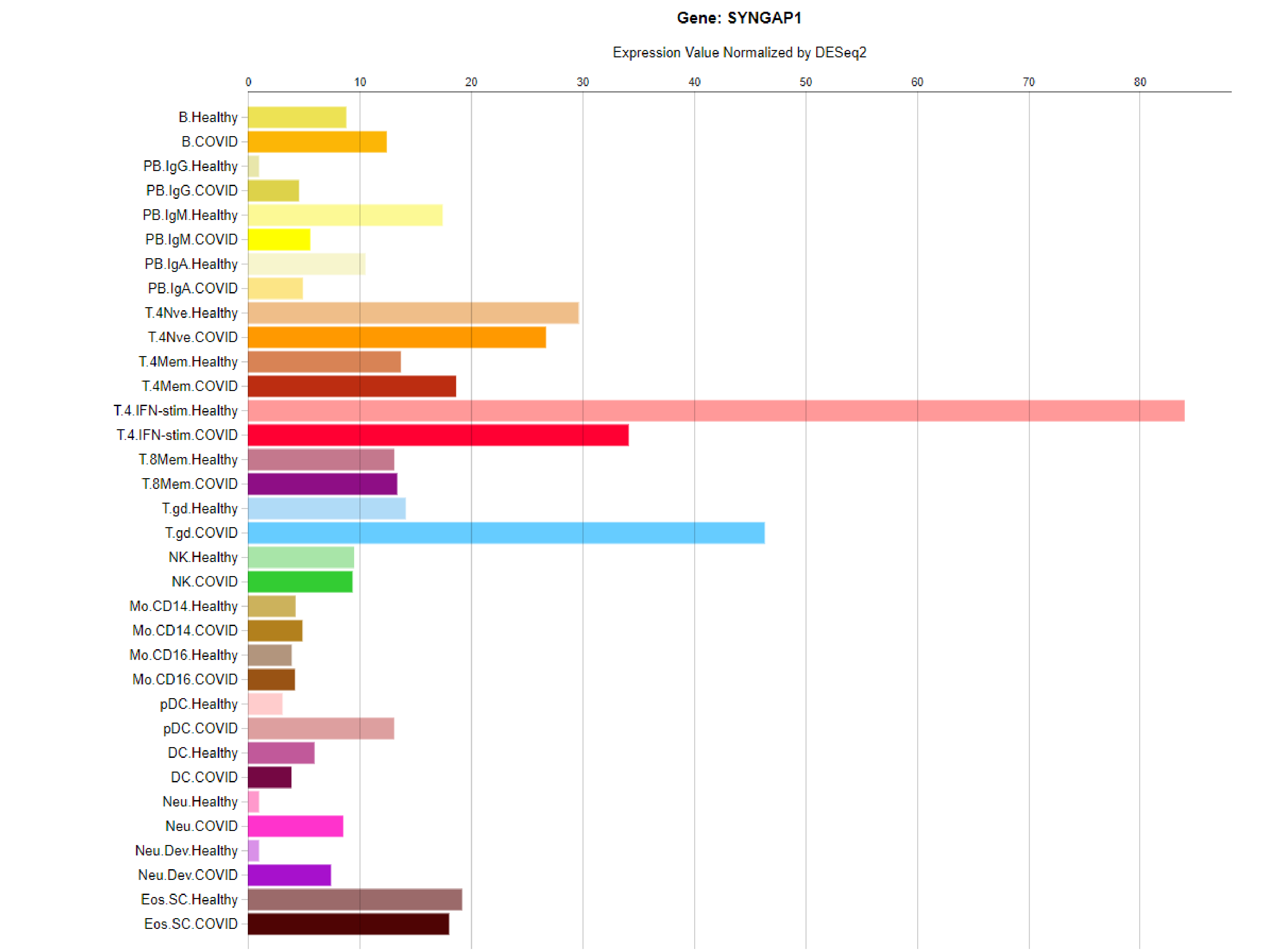

Gene: SYNGAP1
Expression Value Normalized by DESeq2
0
10
20
30
40
50
60
70
80
B.Healthy
B.COVID
PB.IgG.Healthy
PB.IgG.COVID
PB.IgM.Healthy
PB.IgM.COVID
PB.IgA.Healthy
PB.IgA.COVID
T.4Nve.Healthy
T.4Nve.COVID
T.4Mem.Healthy
T.4Mem.COVID
T.4.IFN-stim.Healthy
T.4.IFN-stim.COVID
T.8Mem.Healthy
T.8Mem.COVID
T.gd.Healthy
T.gd.COVID
NK.Healthy
NK.COVID
Mo.CD14.Healthy
Mo.CD14.COVID
Mo.CD16.Healthy
Mo.CD16.COVID
pDC.Healthy
pDC.COVID
DC.Healthy
DC.COVID
Neu.Healthy
Neu.COVID
Neu.Dev.Healthy
Neu.Dev.COVID
Eos.SC.Healthy
Eos.SC.COVID

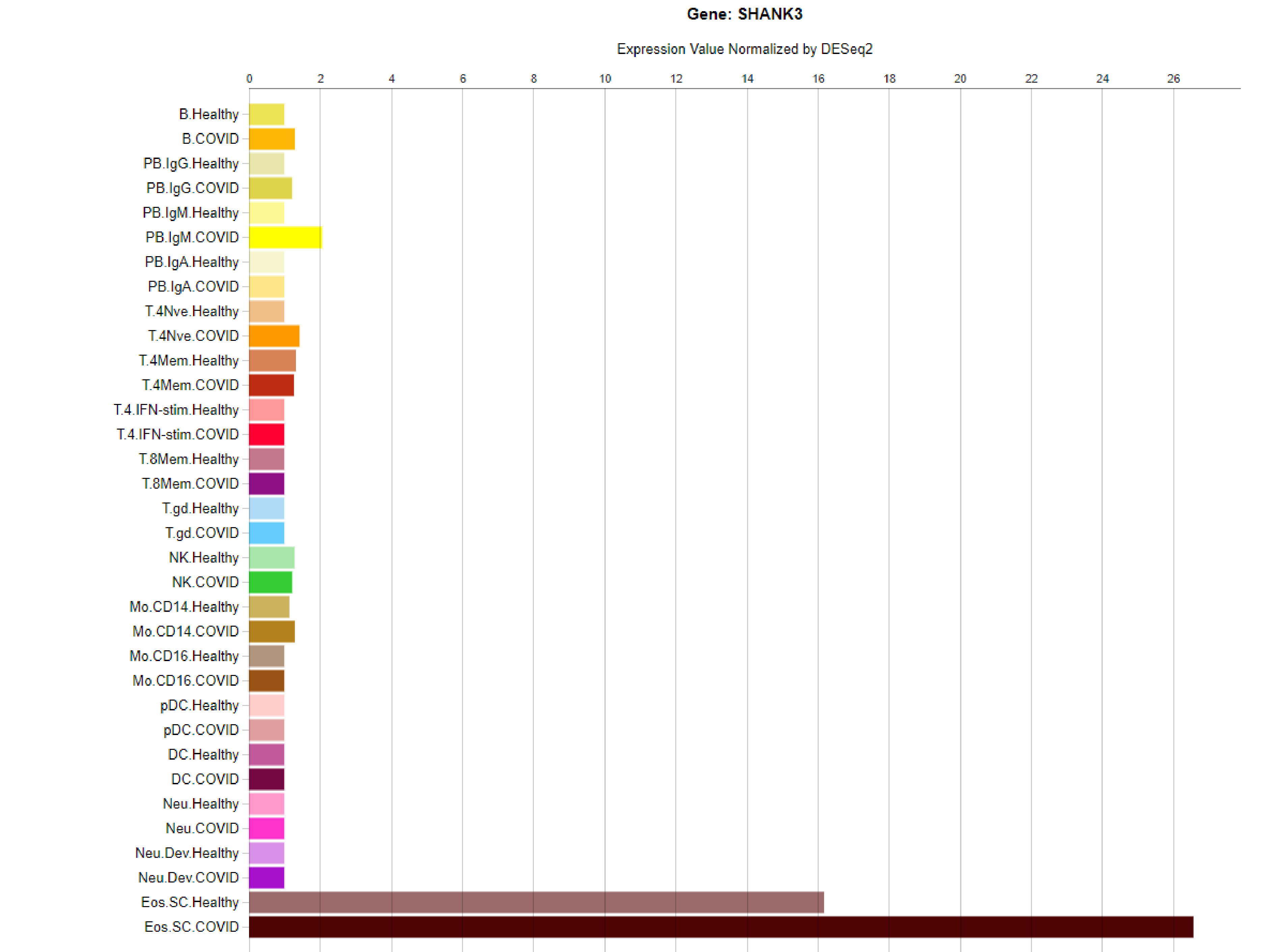

Gene: SHANK3
Expression Value Normalized by DESeq2
0
2
4
6
8
10
12
14
16
18
20
22
24
26
B.Healthy
B.COVID
PB.IgG.Healthy
PB.IgG.COVID
PB.IgM.Healthy
PB.IgM.COVID
PB.IgA.Healthy
PB.IgA.COVID
T.4Nve.Healthy
T.4Nve.COVID
T.4Mem.Healthy
T.4Mem.COVID
T.4.IFN-stim.Healthy
T.4.IFN-stim.COVID
T.8Mem.Healthy
T.8Mem.COVID
T.gd.Healthy
T.gd.COVID
NK.Healthy
NK.COVID
Mo.CD14.Healthy
Mo.CD14.COVID
Mo.CD16.Healthy
Mo.CD16.COVID
pDC.Healthy
pDC.COVID
DC.Healthy
DC.COVID
Neu.Healthy
Neu.COVID
Neu.Dev.Healthy
Neu.Dev.COVID
Eos.SC.Healthy
Eos.SC.COVID

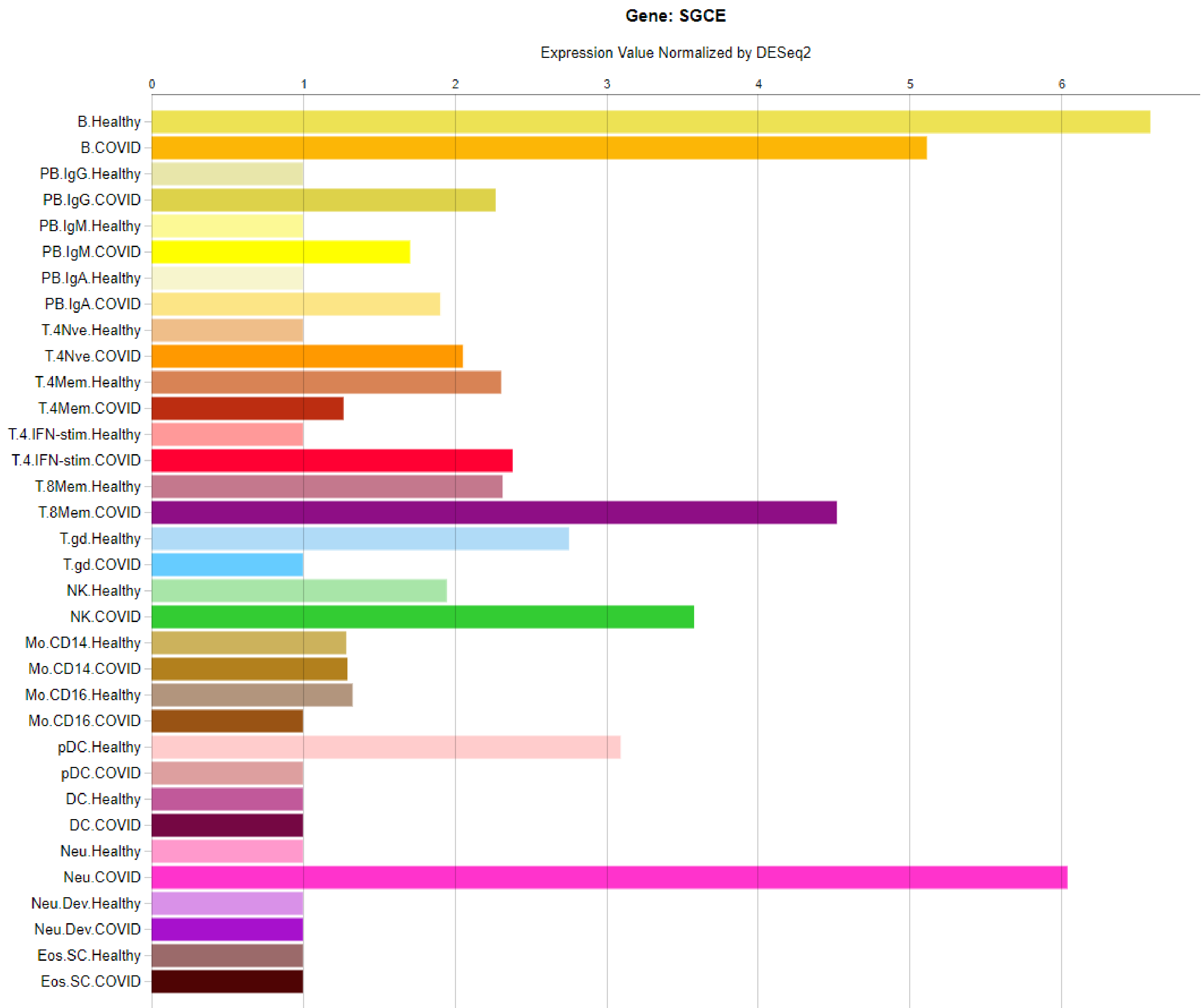

Gene: SGCE
Expression Value Normalized by DESeq2
0
1
2
3
4
5
6
B.Healthy
B.COVID
PB.IgG.Healthy
PB.IgG.COVID
PB.IgM.Healthy
PB.IgM.COVID
PB.IgA.Healthy
PB.IgA.COVID
T.4Nve.Healthy
T.4Nve.COVID
T.4Mem.Healthy
T.4Mem.COVID
T.4.IFN-stim.Healthy
T.4.IFN-stim.COVID
T.8Mem.Healthy
T.8Mem.COVID
T.gd.Healthy
T.gd.COVID
NK.Healthy
NK.COVID
Mo.CD14.Healthy
Mo.CD14.COVID
Mo.CD16.Healthy
Mo.CD16.COVID
pDC.Healthy
pDC.COVID
DC.Healthy
DC.COVID
Neu.Healthy
Neu.COVID
Neu.Dev.Healthy
Neu.Dev.COVID
Eos.SC.Healthy
Eos.SC.COVID

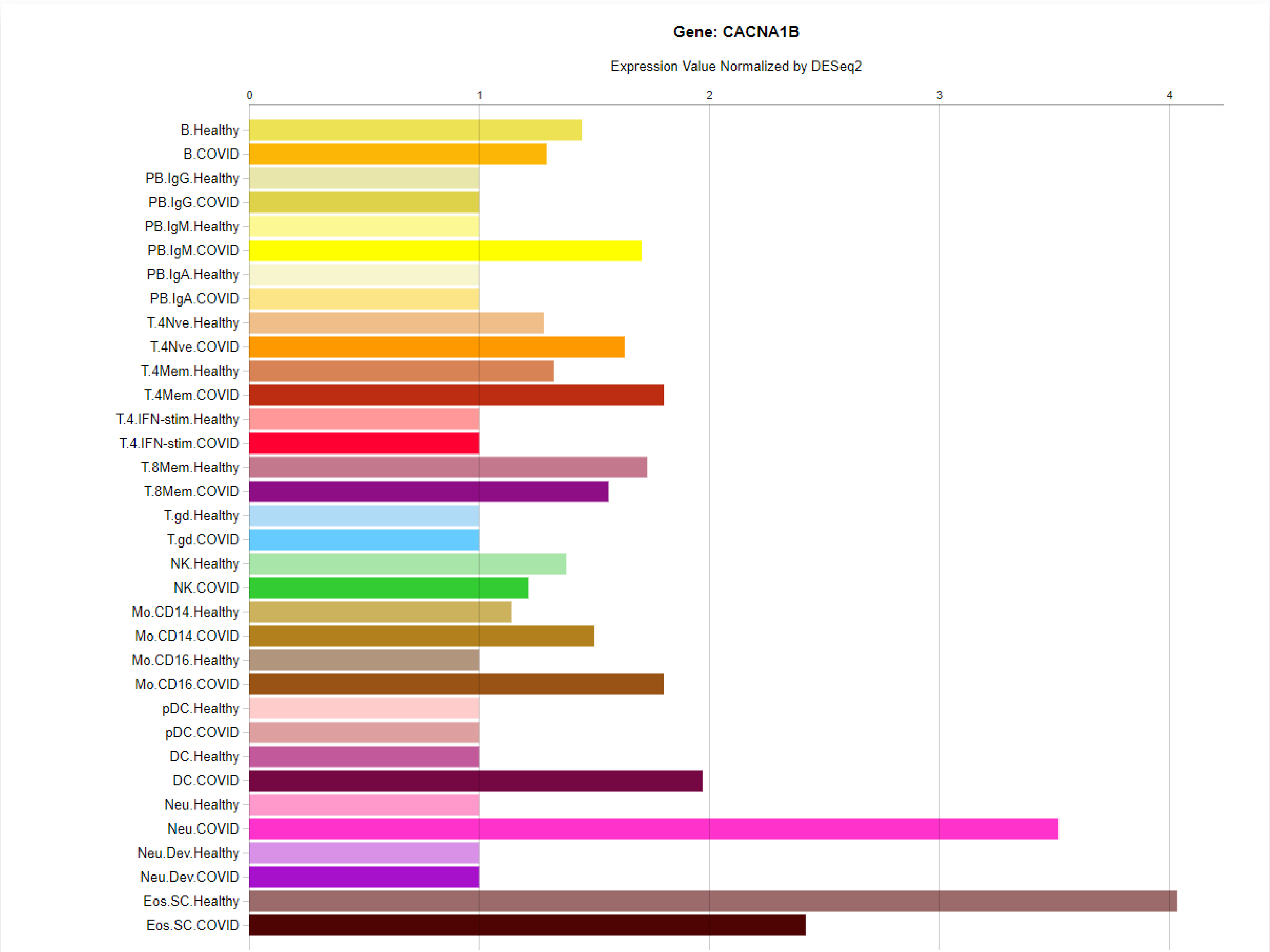
Gene: CACNA1B
Expression Value Normalized by DESeq2
0
1
2
3
4
B.Healthy
B.COVID
PB.IgG.Healthy
PB.IgG.COVID
PB.IgM.Healthy
PB.IgM.COVID
PB.IgA.Healthy
PB.IgA.COVID
T.4Nve.Healthy
T.4Nve.COVID
T.4Mem.Healthy
T.4Mem.COVID
T.4.IFN-stim.Healthy
T.4.IFN-stim.COVID
T.8Mem.Healthy
T.8Mem.COVID
T.gd.Healthy
T.gd.COVID
NK.Healthy
NK.COVID
Mo.CD14.Healthy
Mo.CD14.COVID
Mo.CD16.Healthy
Mo.CD16.COVID
pDC.Healthy
pDC.COVID
DC.Healthy
DC.COVID
Neu.Healthy
Neu.COVID
Neu.Dev.Healthy
Neu.Dev.COVID
Eos.SC.Healthy
Eos.SC.COVID

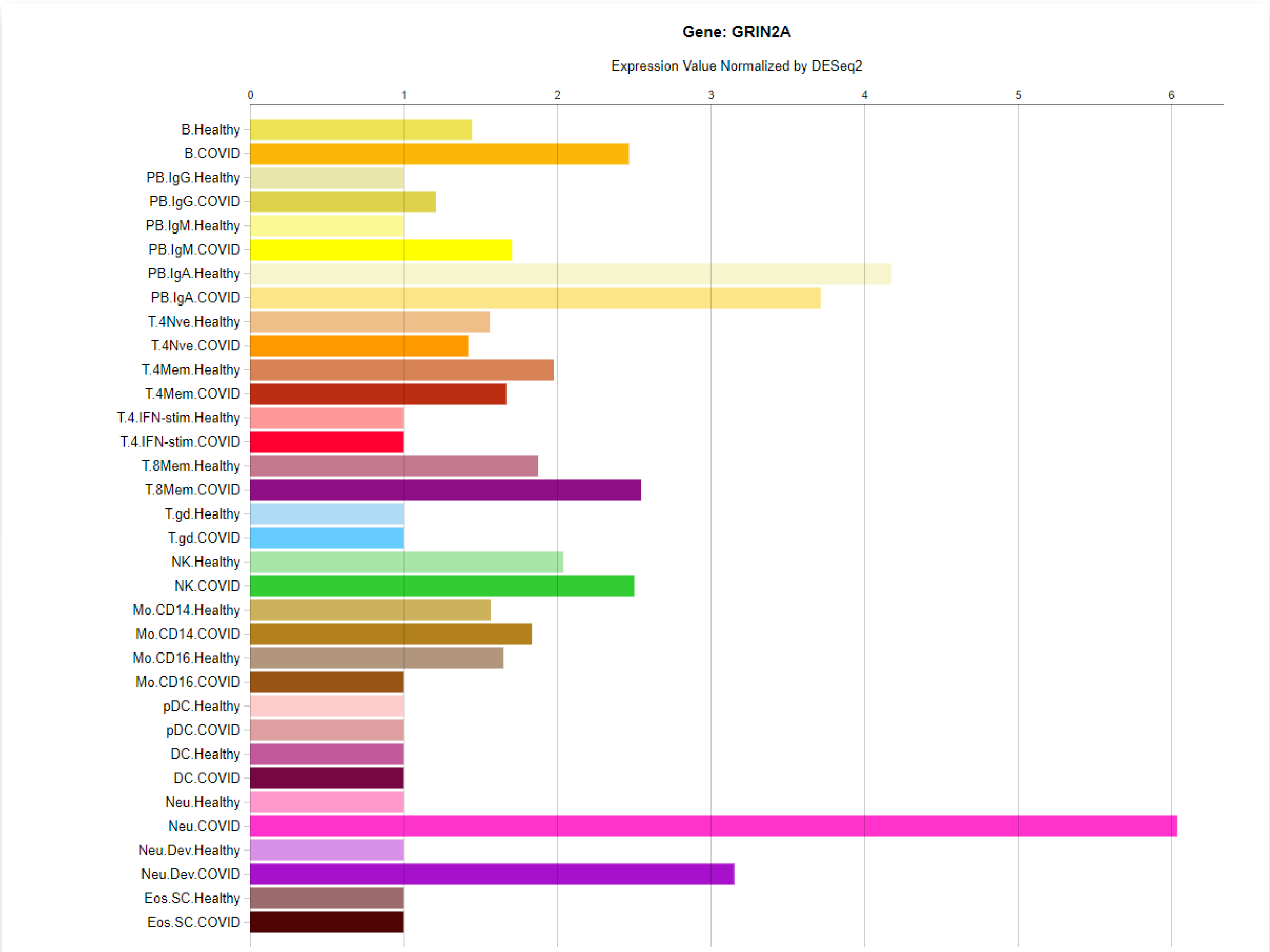
Gene: GRIN2A
Expression Value Normalized by DESeq2
0
1
2
3
4
5
6
B.Healthy
B.COVID
PB.IgG.Healthy
PB.IgG.COVID
PB.IgM.Healthy
PB.IgM.COVID
PB.IgA.Healthy
PB.IgA.COVID
T.4Nve.Healthy
T.4Nve.COVID
T.4Mem.Healthy
T.4Mem.COVID
T.4.IFN-stim.Healthy
T.4.IFN-stim.COVID
T.8Mem.Healthy
T.8Mem.COVID
T.gd.Healthy
T.gd.COVID
NK.Healthy
NK.COVID
Mo.CD14.Healthy
Mo.CD14.COVID
Mo.CD16.Healthy
Mo.CD16.COVID
pDC.Healthy
pDC.COVID
DC.Healthy
DC.COVID
Neu.Healthy
Neu.COVID
Neu.Dev.Healthy
Neu.Dev.COVID
Eos.SC.Healthy
Eos.SC.COVID

Supplementary Figure S2: expression pattern of PANS candidate genes in human tissue from GTEx database

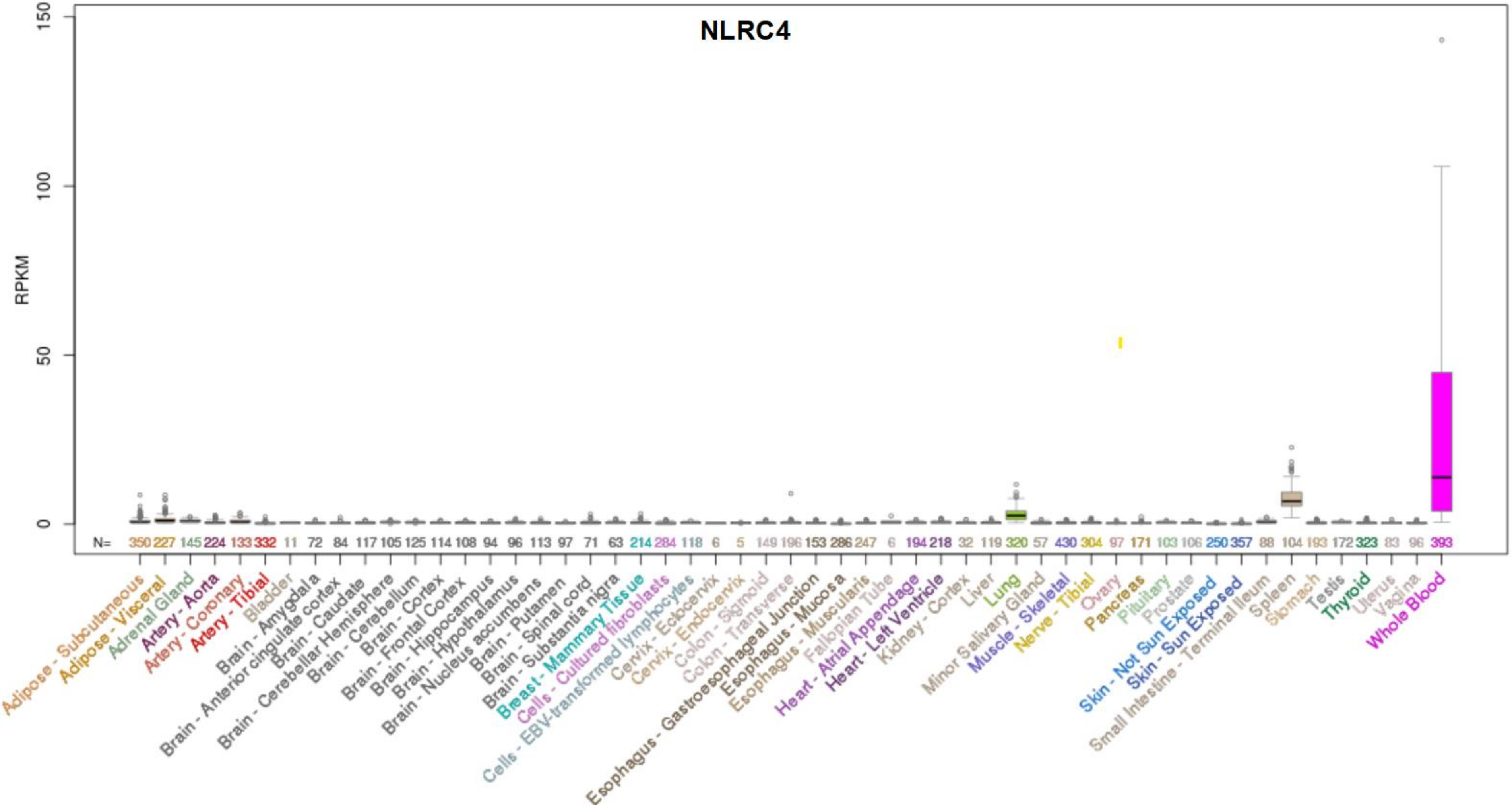
NLRC4
RPKM
150
100
50
0
N=
350 227 145 224 133 332 11 72 84 117 105 125 114 108 94 96 113 97 71 63 214 284 118 6 5 149 196 153 286 247 6 194 218 32 119 320 57 430 304 97 171 103 106 250 357 88 104 193 172 323 83 96 393
Adipose - Subcutaneous
Adipose - Visceral
Adrenal Gland
Artery - Aorta
Artery - Coronary
Artery - Tibial
Bladder
Brain - Amygdala
Brain - Anterior cingulate cortex
Brain - Caudate
Brain - Cerebellar Hemisphere
Brain - Cerebellum
Brain - Cortex
Brain - Frontal Cortex
Brain - Hippocampus
Brain - Hypothalamus
Brain - Nucleus accumbens
Brain - Putamen
Brain - Spinal cord
Brain - Substantia nigra
Breast - Mammary Tissue
Cells - Cultured fibroblasts
Cells - EBV-transformed lymphocytes
Cervix - Ectocervix
Cervix - Endocervix
Colon - Sigmoid
Colon - Transverse
Esophagus - Gastroesophageal Junction
Esophagus - Mucosa
Esophagus - Muscularis
Fallopian Tube
Heart - Atrial Appendage
Heart - Left Ventricle
Kidney - Cortex
Liver
Lung
Minor Salivary Gland
Muscle - Skeletal
Nerve - Tibial
Ovary
Pancreas
Pituitary
Prostate
Skin - Not Sun Exposed
Skin - Sun Exposed
Small Intestine - Terminal Ileum
Spleen
Stomach
Testis
Thyroid
Uterus
Vagina
Whole Blood

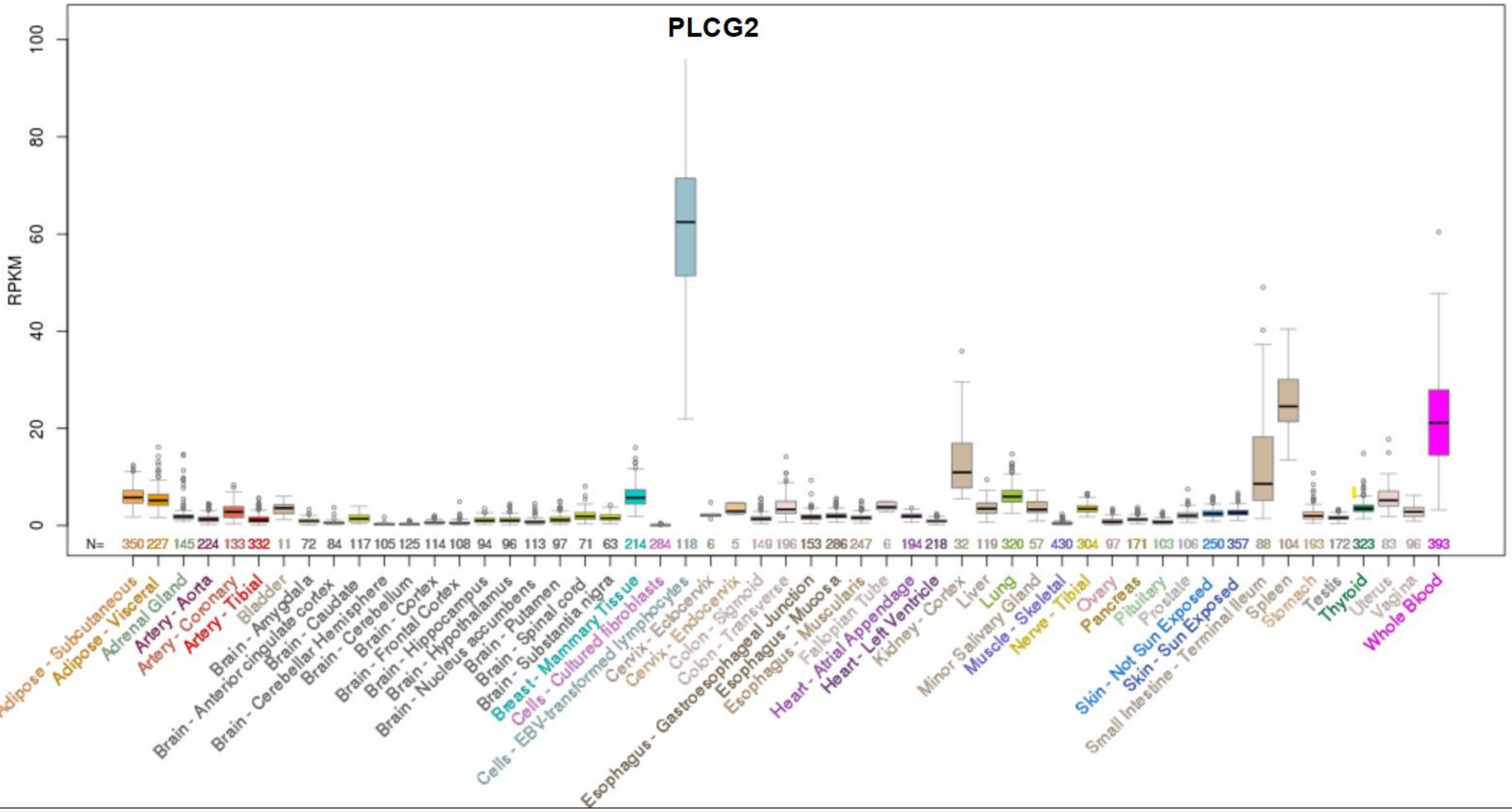
PLCG2
RPKM
0
20
40
60
80
100
N=
350 227 145 224 133 332 11 72 84 117 105 125 114 108 94 96 113 97 71 63 214 284 118 6 5 149 196 153 286 247 6 194 218 32 119 320 57 430 304 97 171 103 106 250 357 88 104 193 172 323 83 96 393
Adipose - Subcutaneous
Adipose - Visceral
Adrenal Gland
Artery - Aorta
Artery - Coronary
Artery - Tibial
Bladder
Brain - Amygdala
Brain - Anterior cingulate cortex
Brain - Caudate
Brain - Cerebellar Hemisphere
Brain - Cerebellum
Brain - Cortex
Brain - Frontal Cortex
Brain - Hippocampus
Brain - Hypothalamus
Brain - Nucleus accumbens
Brain - Putamen
Brain - Spinal cord
Brain - Substantia nigra
Breast - Mammary Tissue
Cells - Cultured fibroblasts
Cells - EBV-transformed lymphocytes
Cervix - Ectocervix
Cervix - Endocervix
Colon - Sigmoid
Colon - Transverse
Esophagus - Gastroesophageal Junction
Esophagus - Mucosa
Esophagus - Muscularis
Fallopian Tube
Heart - Atrial Appendage
Heart - Left Ventricle
Kidney - Cortex
Liver
Lung
Minor Salivary Gland
Muscle - Skeletal
Nerve - Tibial
Ovary
Pancreas
Pituitary
Prostate
Skin - Not Sun Exposed
Skin - Sun Exposed
Small Intestine - Terminal Ileum
Spleen
Stomach
Testis
Thyroid
Uterus
Vagina
Whole Blood

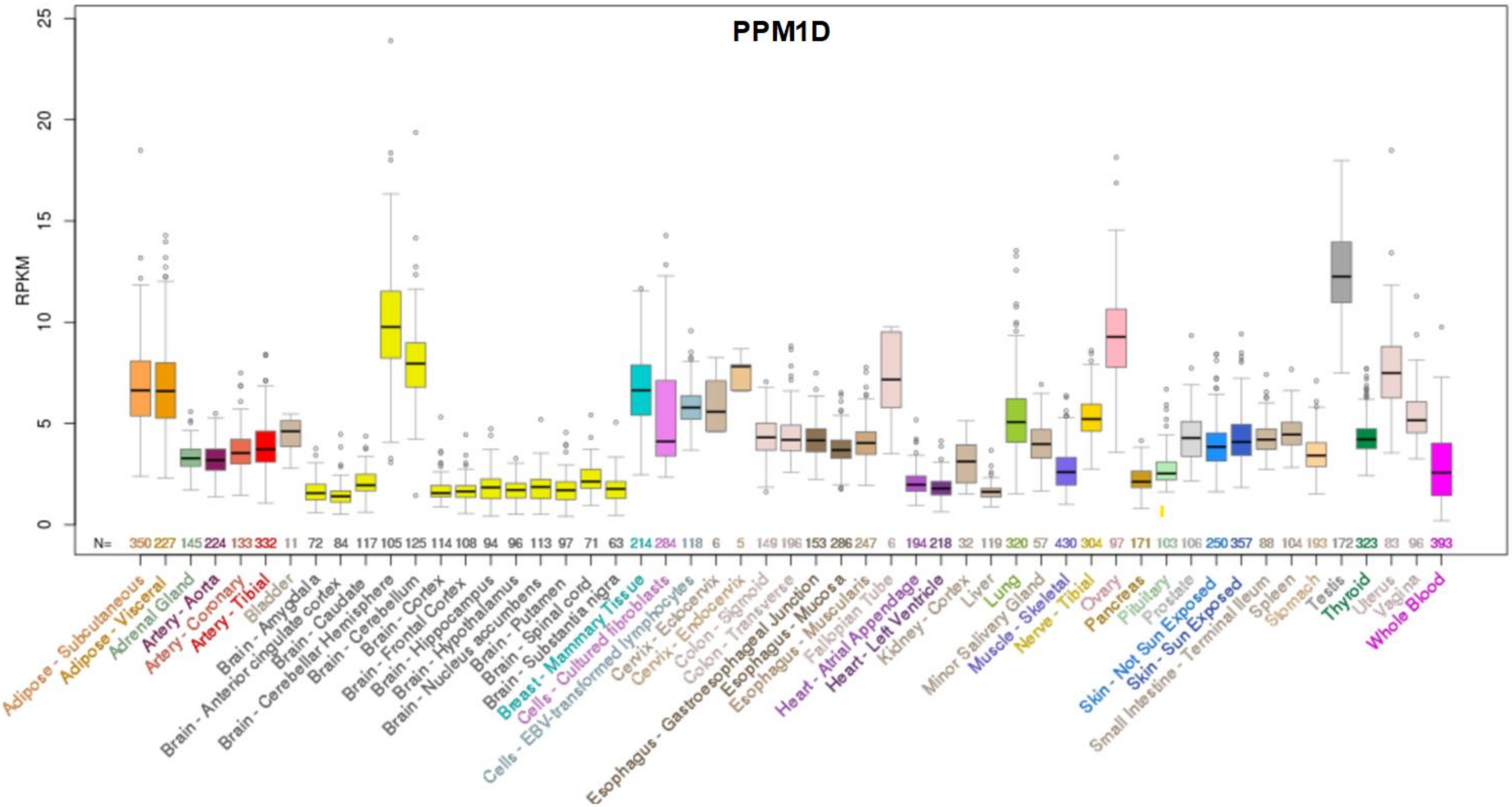
PPM1D
RPKM
0
5
10
15
20
25
N=
350 227 145 224 133 332 11 72 84 117 105 125 114 108 94 96 113 97 71 63 214 284 118 6 5 149 196 153 286 247 6 194 218 32 119 320 57 430 304 97 171 103 106 250 357 88 104 193 172 323 83 96 393
Adipose - Subcutaneous
Adipose - Visceral
Adrenal Gland
Artery - Aorta
Artery - Coronary
Artery - Tibial
Bladder
Brain - Amygdala
Brain - Anterior cingulate cortex
Brain - Caudate
Brain - Cerebellar Hemisphere
Brain - Cerebellum
Brain - Cortex
Brain - Frontal Cortex
Brain - Hippocampus
Brain - Hypothalamus
Brain - Nucleus accumbens
Brain - Putamen
Brain - Spinal cord
Brain - Substantia nigra
Breast - Mammary Tissue
Cells - Cultured fibroblasts
Cells - EBV-transformed lymphocytes
Cervix - Ectocervix
Cervix - Endocervix
Colon - Sigmoid
Colon - Transverse
Esophagus - Gastroesophageal Junction
Esophagus - Mucosa
Esophagus - Muscularis
Fallopian Tube
Heart - Atrial Appendage
Heart - Left Ventricle
Kidney - Cortex
Liver
Lung
Minor Salivary Gland
Muscle - Skeletal
Nerve - Tibial
Ovary
Pancreas
Pituitary
Prostate
Skin - Not Sun Exposed
Skin - Sun Exposed
Small Intestine - Terminal Ileum
Spleen
Stomach
Testis
Thyroid
Uterus
Vagina
Whole Blood

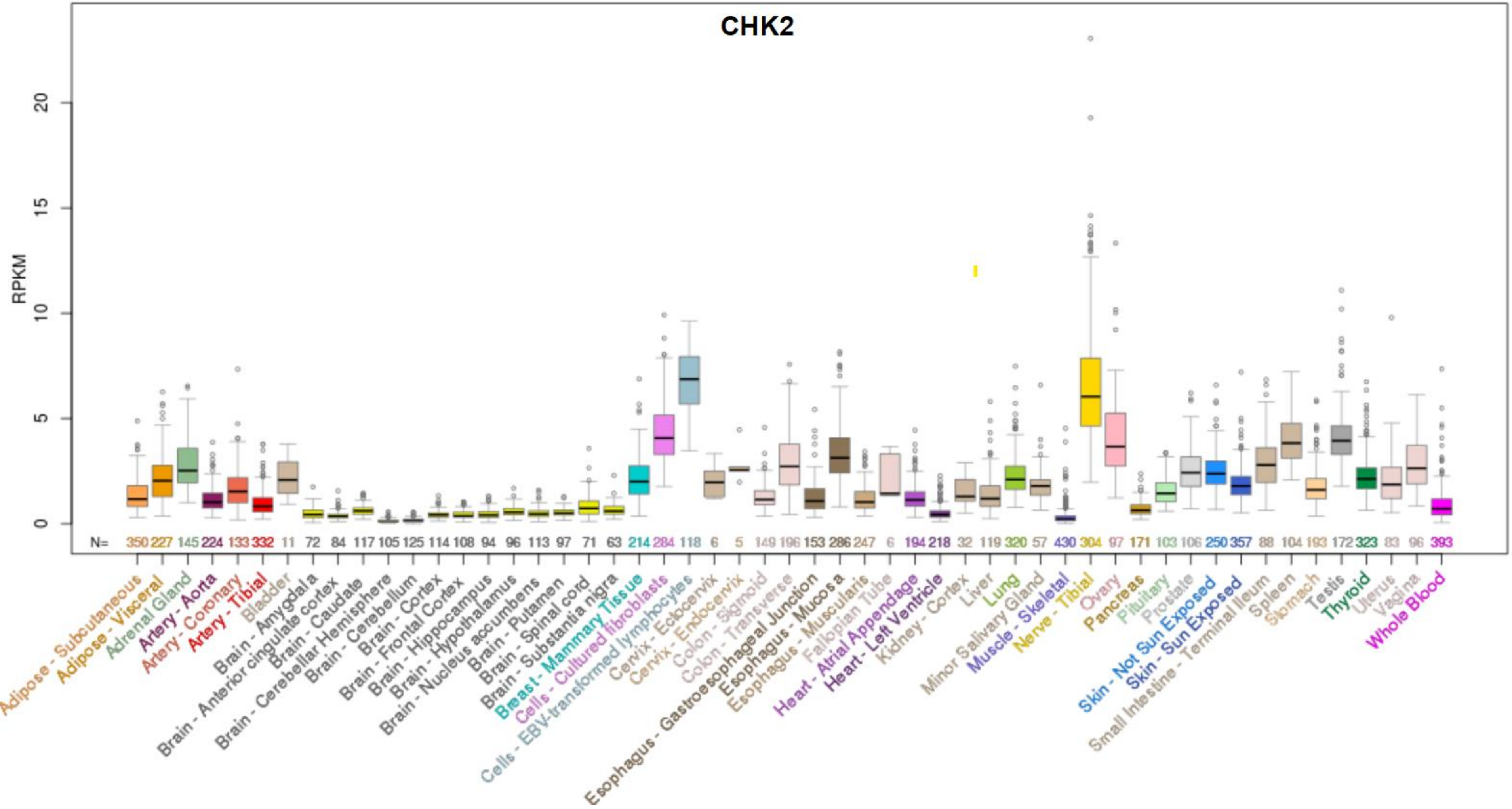
CHK2
RPKM
0
5
10
15
20
N=
350 227 145 224 133 332 11 72 84 117 105 125 114 108 94 96 113 97 71 63 214 284 118 6 5 149 196 153 286 247 6 194 218 32 119 320 57 430 304 97 171 103 106 250 357 88 104 193 172 323 83 96 393
Adipose - Subcutaneous
Adipose - Visceral
Adrenal Gland
Artery - Aorta
Artery - Coronary
Artery - Tibial
Bladder
Brain - Amygdala
Brain - Anterior cingulate cortex
Brain - Caudate
Brain - Cerebellar Hemisphere
Brain - Cerebellum
Brain - Cortex
Brain - Frontal Cortex
Brain - Hippocampus
Brain - Hypothalamus
Brain - Nucleus accumbens
Brain - Putamen
Brain - Spinal cord
Brain - Substantia nigra
Breast - Mammary Tissue
Cells - Cultured fibroblasts
Cells - EBV-transformed lymphocytes
Cervix - Ectocervix
Cervix - Endocervix
Colon - Sigmoid
Colon - Transverse
Esophagus - Gastroesophageal Junction
Esophagus - Mucosa
Esophagus - Muscularis
Fallopian Tube
Heart - Atrial Appendage
Heart - Left Ventricle
Kidney - Cortex
Liver
Lung
Minor Salivary Gland
Muscle - Skeletal
Nerve - Tibial
Ovary
Pancreas
Pituitary
Prostate
Skin - Not Sun Exposed
Skin - Sun Exposed
Small Intestine - Terminal Ileum
Spleen
Stomach
Testis
Thyroid
Uterus
Vagina
Whole Blood

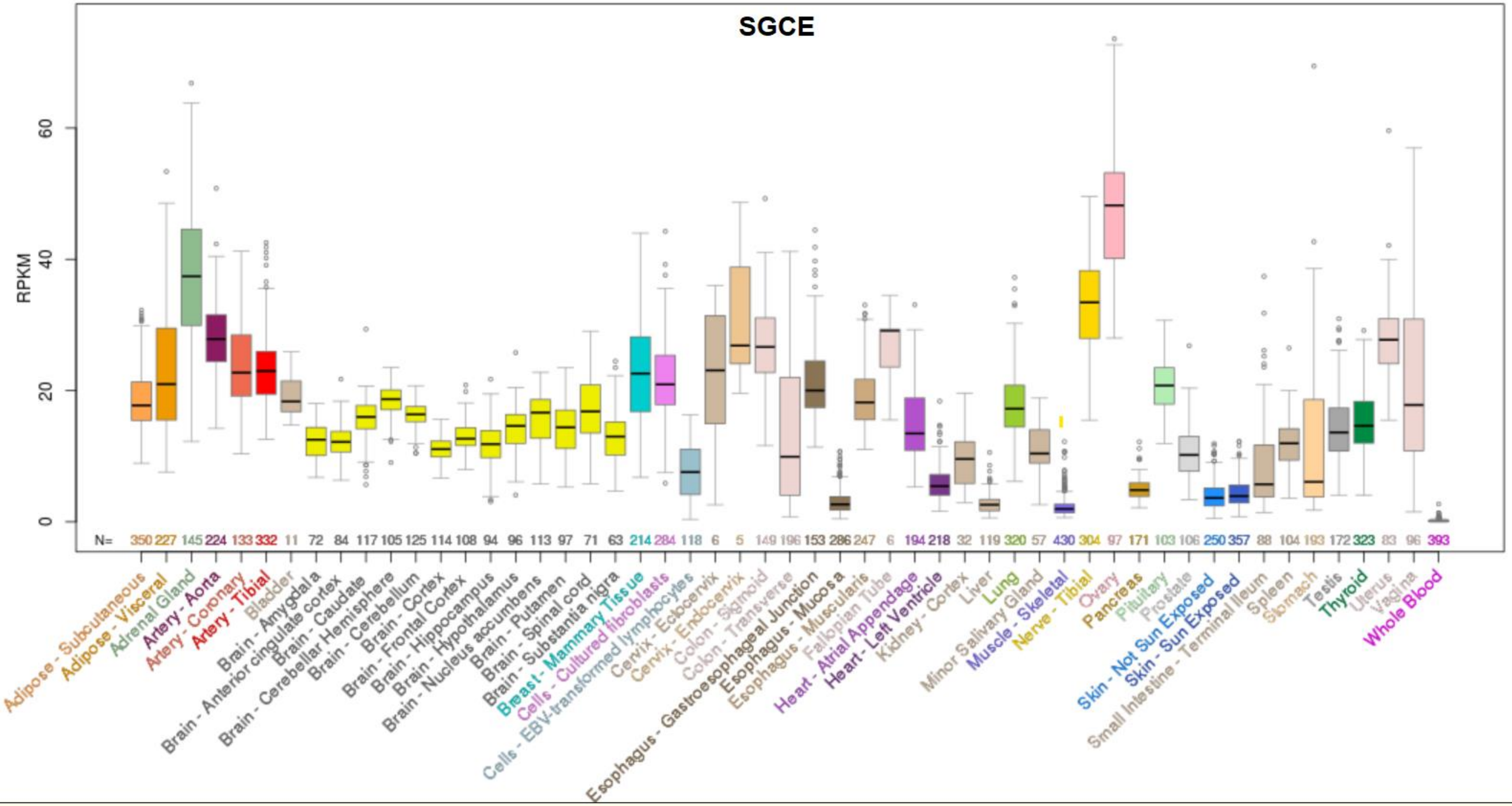
SGCE
RPKM
0
20
40
60
N=
350 227 145 224 133 332 11 72 84 117 105 125 114 108 94 96 113 97 71 63 214 284 118 6 5 149 196 153 286 247 6 194 218 32 119 320 57 430 304 97 171 103 106 250 357 88 104 193 172 323 83 96 393
Adipose - Subcutaneous
Adipose - Visceral
Adrenal Gland
Artery - Aorta
Artery - Coronary
Artery - Tibial
Bladder
Brain - Amygdala
Brain - Anterior cingulate cortex
Brain - Caudate
Brain - Cerebellar Hemisphere
Brain - Cerebellum
Brain - Cortex
Brain - Frontal Cortex
Brain - Hippocampus
Brain - Hypothalamus
Brain - Nucleus accumbens
Brain - Putamen
Brain - Spinal cord
Brain - Substantia nigra
Breast - Mammary Tissue
Cells - Cultured fibroblasts
Cells - EBV-transformed lymphocytes
Cervix - Ectocervix
Cervix - Endocervix
Colon - Sigmoid
Colon - Transverse
Esophagus - Gastroesophageal Junction
Esophagus - Mucosa
Esophagus - Muscularis
Fallopian Tube
Heart - Atrial Appendage
Heart - Left Ventricle
Kidney - Cortex
Liver
Lung
Minor Salivary Gland
Muscle - Skeletal
Nerve - Tibial
Ovary
Pancreas
Pituitary
Prostate
Skin - Not Sun Exposed
Skin - Sun Exposed
Small Intestine - Terminal Ileum
Spleen
Stomach
Testis
Thyroid
Uterus
Vagina
Whole Blood

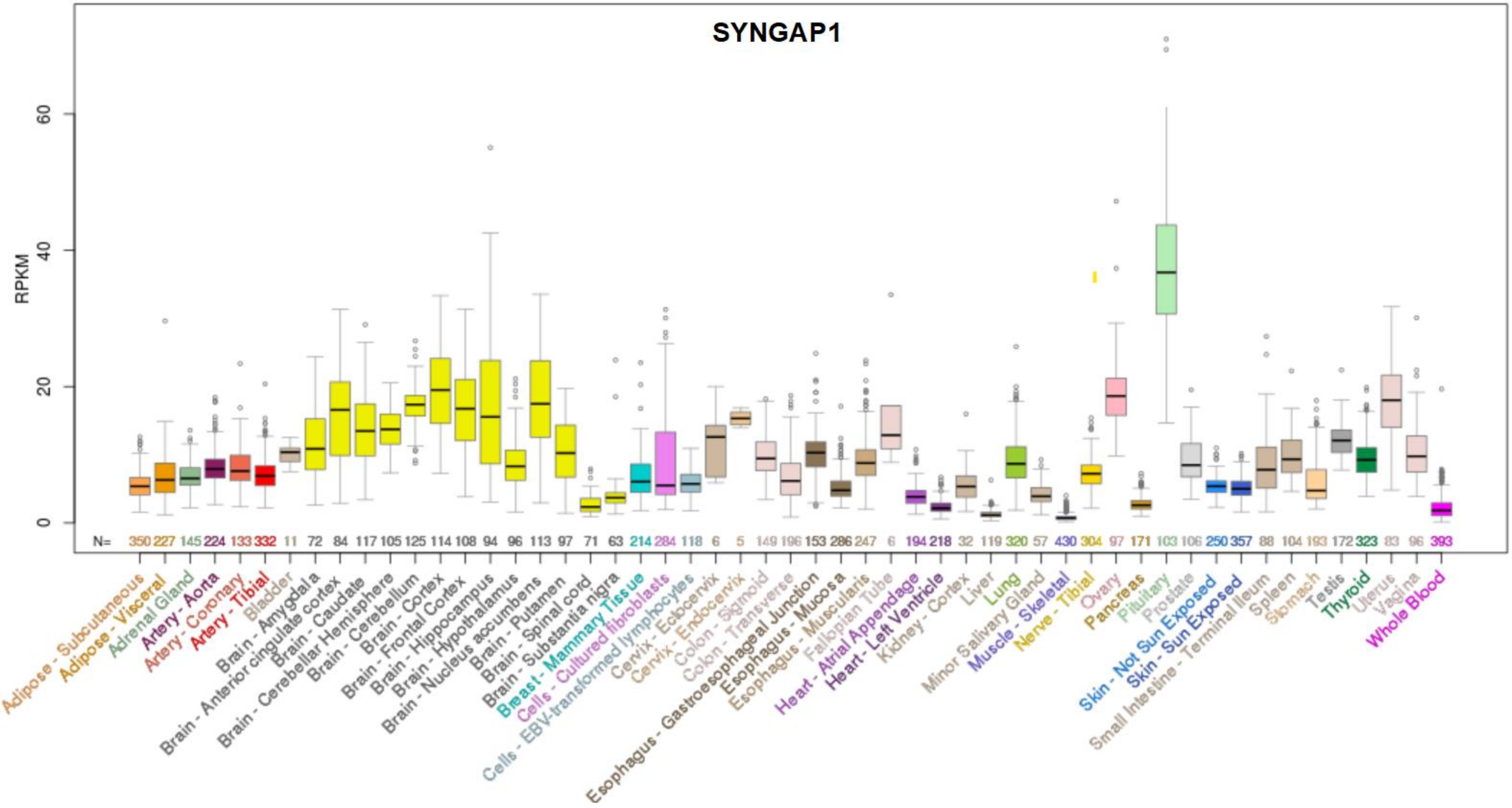
SYNGAP1
RPKM
60
40
20
0
N=
350 227 145 224 133 332 11 72 84 117 105 125 114 108 94 96 113 97 71 63 214 284 118 6 5 149 196 153 286 247 6 194 218 32 119 320 57 430 304 97 171 103 106 250 357 88 104 193 172 323 83 96 393
Adipose - Subcutaneous
Adipose - Visceral
Adrenal Gland
Artery - Aorta
Artery - Coronary
Artery - Tibial
Bladder
Brain - Amygdala
Brain - Anterior cingulate cortex
Brain - Caudate
Brain - Cerebellar Hemisphere
Brain - Cerebellum
Brain - Cortex
Brain - Frontal Cortex
Brain - Hippocampus
Brain - Hypothalamus
Brain - Nucleus accumbens
Brain - Putamen
Brain - Spinal cord
Brain - Substantia nigra
Breast - Mammary Tissue
Cells - Cultured fibroblasts
Cells - EBV-transformed lymphocytes
Cervix - Ectocervix
Cervix - Endocervix
Colon - Sigmoid
Colon - Transverse
Esophagus - Gastroesophageal Junction
Esophagus - Mucosa
Esophagus - Muscularis
Fallopian Tube
Heart - Atrial Appendage
Heart - Left Ventricle
Kidney - Cortex
Liver
Lung
Minor Salivary Gland
Muscle - Skeletal
Nerve - Tibial
Ovary
Pancreas
Pituitary
Prostate
Skin - Not Sun Exposed
Skin - Sun Exposed
Small Intestine - Terminal Ileum
Spleen
Stomach
Testis
Thyroid
Uterus
Vagina
Whole Blood

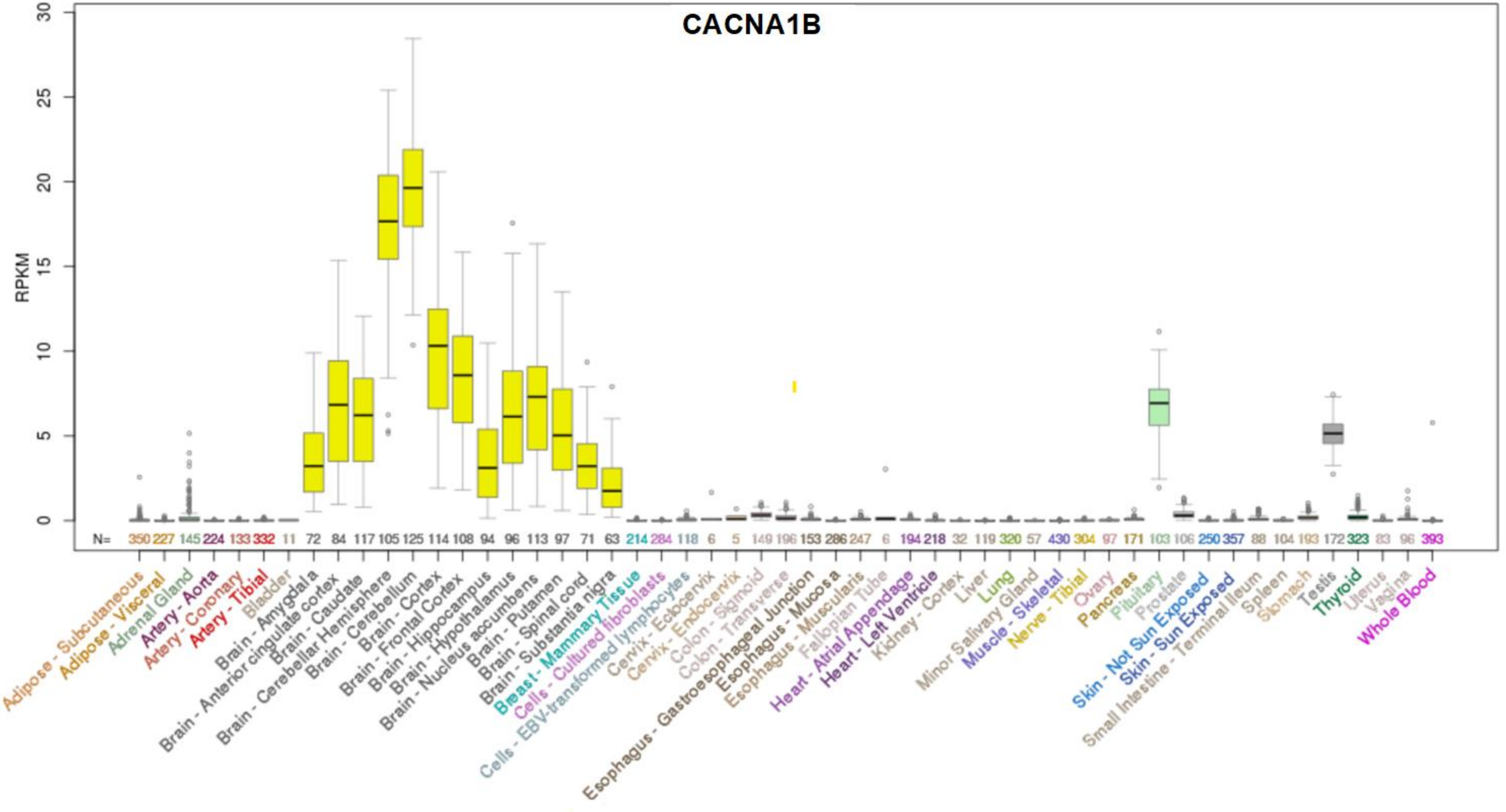

CACNA1B
RPKM
0
5
10
15
20
25
30
N=
350 227 145 224 133 332 11 72 84 117 105 125 114 108 94 96 113 97 71 63 214 284 118 6 5 149 196 153 286 247 6 194 218 32 119 320 57 430 304 97 171 103 106 250 357 88 104 193 172 323 83 96 393
Adipose - Subcutaneous
Adipose - Visceral
Adrenal Gland
Artery - Aorta
Artery - Coronary
Artery - Tibial
Bladder
Brain - Amygdala
Brain - Anterior cingulate cortex
Brain - Caudate
Brain - Cerebellar Hemisphere
Brain - Cerebellum
Brain - Cortex
Brain - Frontal Cortex
Brain - Hippocampus
Brain - Hypothalamus
Brain - Nucleus accumbens
Brain - Putamen
Brain - Spinal cord
Brain - Substantia nigra
Breast - Mammary Tissue
Cells - Cultured fibroblasts
Cells - EBV-transformed lymphocytes
Cervix - Ectocervix
Cervix - Endocervix
Colon - Sigmoid
Colon - Transverse
Esophagus - Gastroesophageal Junction
Esophagus - Mucosa
Esophagus - Muscularis
Fallopian Tube
Heart - Atrial Appendage
Heart - Left Ventricle
Kidney - Cortex
Liver
Lung
Minor Salivary Gland
Muscle - Skeletal
Nerve - Tibial
Ovary
Pancreas
Pituitary
Prostate
Skin - Not Sun Exposed
Skin - Sun Exposed
Small Intestine - Terminal Ileum
Spleen
Stomach
Testis
Thyroid
Uterus
Vagina
Whole Blood

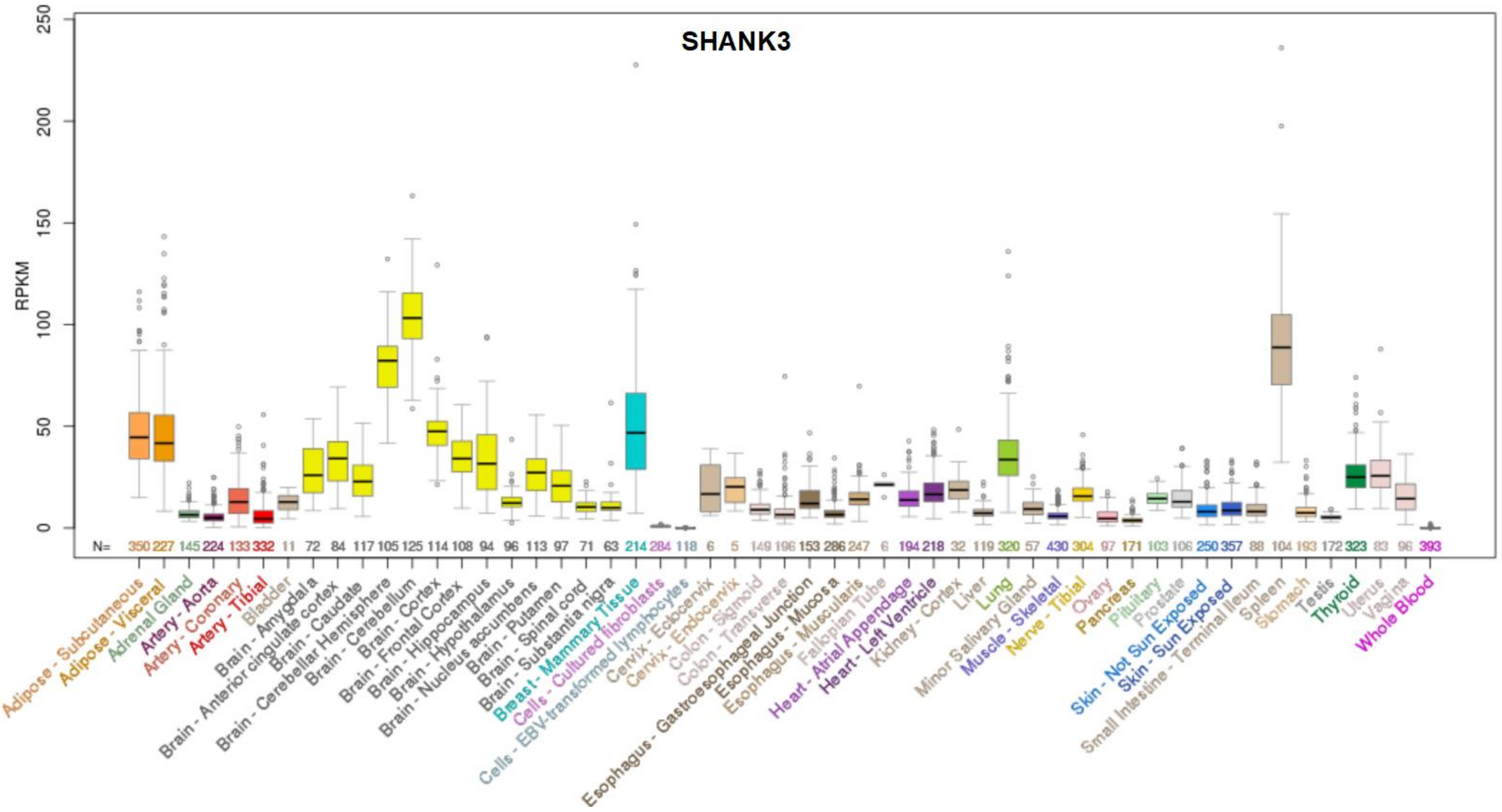
SHANK3
RPKM
250
200
150
100
50
0
N=
350 227 145 224 133 332 11 72 84 117 105 125 114 108 94 96 113 97 71 63 214 284 118 6 5 149 196 153 286 247 6 194 218 32 119 320 57 430 304 97 171 103 106 250 357 88 104 193 172 323 83 96 393
Adipose - Subcutaneous
Adipose - Visceral
Adrenal Gland
Artery - Aorta
Artery - Coronary
Artery - Tibial
Bladder
Brain - Amygdala
Brain - Anterior cingulate cortex
Brain - Caudate
Brain - Cerebellar Hemisphere
Brain - Cerebellum
Brain - Cortex
Brain - Frontal Cortex
Brain - Hippocampus
Brain - Hypothalamus
Brain - Nucleus accumbens
Brain - Putamen
Brain - Spinal cord
Brain - Substantia nigra
Breast - Mammary Tissue
Cells - Cultured fibroblasts
Cells - EBV-transformed lymphocytes
Cervix - Ectocervix
Cervix - Endocervix
Colon - Sigmoid
Colon - Transverse
Esophagus - Gastroesophageal Junction
Esophagus - Mucosa
Esophagus - Muscularis
Fallopian Tube
Heart - Atrial Appendage
Heart - Left Ventricle
Kidney - Cortex
Liver
Lung
Minor Salivary Gland
Muscle - Skeletal
Nerve - Tibial
Ovary
Pancreas
Pituitary
Prostate
Skin - Not Sun Exposed
Skin - Sun Exposed
Small Intestine - Terminal Ileum
Spleen
Stomach
Testis
Thyroid
Uterus
Vagina
Whole Blood

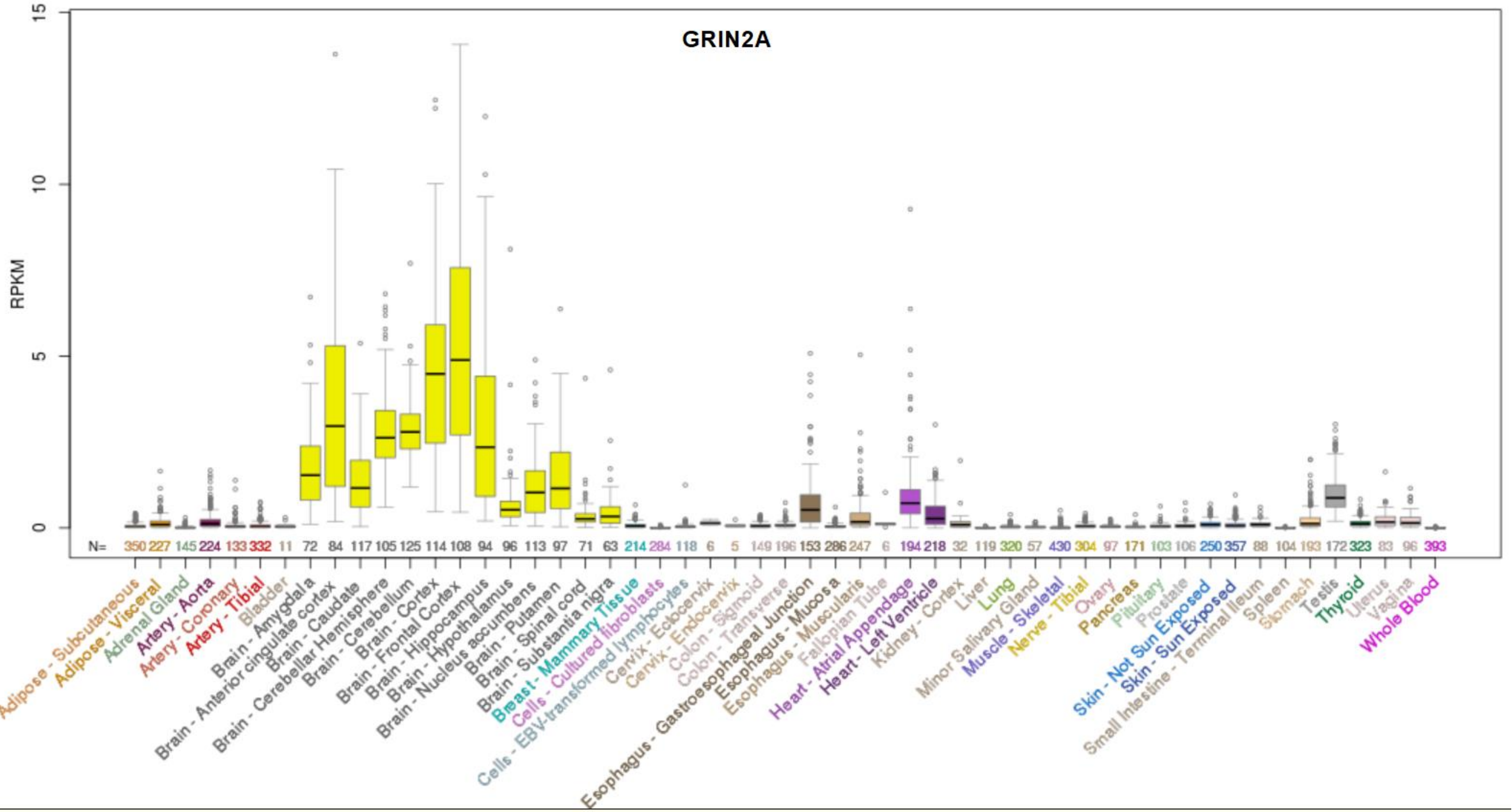
GRIN2A
RPKM
0
5
10
15
N=
350 227 145 224 133 332 11 72 84 117 105 125 114 108 94 96 113 97 71 63 214 284 118 6 5 149 196 153 286 247 6 194 218 32 119 320 57 430 304 97 171 103 106 250 357 88 104 193 172 323 83 96 393
Adipose - Subcutaneous
Adipose - Visceral
Adrenal Gland
Artery - Aorta
Artery - Coronary
Artery - Tibial
Bladder
Brain - Amygdala
Brain - Anterior cingulate cortex
Brain - Caudate
Brain - Cerebellar Hemisphere
Brain - Cerebellum
Brain - Cortex
Brain - Frontal Cortex
Brain - Hippocampus
Brain - Hypothalamus
Brain - Nucleus accumbens
Brain - Putamen
Brain - Spinal cord
Brain - Substantia nigra
Breast - Mammary Tissue
Cells - Cultured fibroblasts
Cells - EBV-transformed lymphocytes
Cervix - Ectocervix
Cervix - Endocervix
Colon - Sigmoid
Colon - Transverse
Esophagus - Gastroesophageal Junction
Esophagus - Mucosa
Esophagus - Muscularis
Fallopian Tube
Heart - Atrial Appendage
Heart - Left Ventricle
Kidney - Cortex
Liver
Lung
Minor Salivary Gland
Muscle - Skeletal
Nerve - Tibial
Ovary
Pancreas
Pituitary
Prostate
Skin - Not Sun Exposed
Skin - Sun Exposed
Small Intestine - Terminal Ileum
Spleen
Stomach
Testis
Thyroid
Uterus
Vagina
Whole Blood

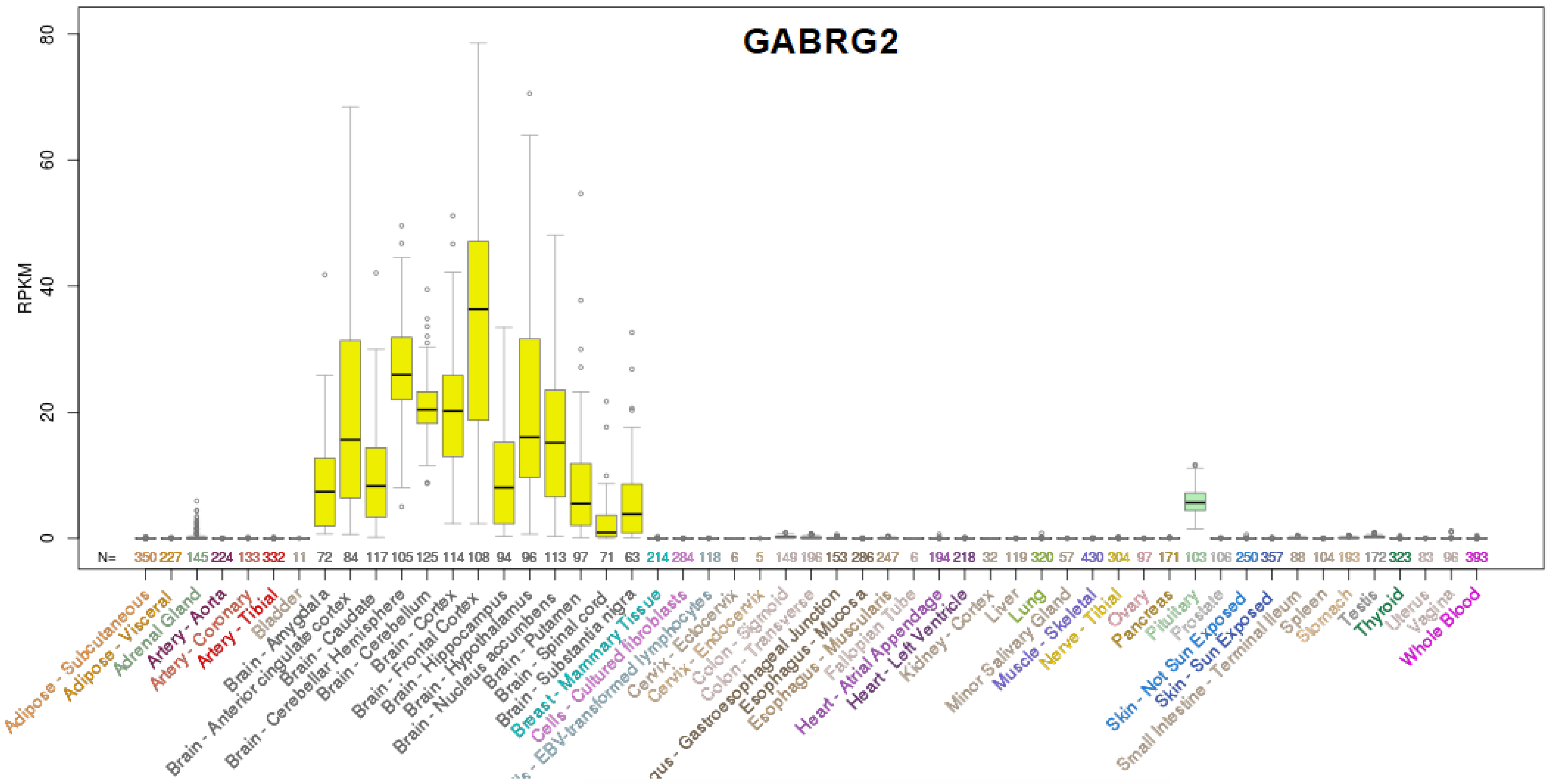
GABRG2
RPKM
0
20
40
60
80
N=
350 227 145 224 133 332 11 72 84 117 105 125 114 108 94 96 113 97 71 63 214 284 118 6 5 149 196 153 286 247 6 194 218 32 119 320 57 430 304 97 171 103 106 250 357 88 104 193 172 323 83 96 393
Adipose - Subcutaneous
Adipose - Visceral
Adrenal Gland
Artery - Aorta
Artery - Coronary
Artery - Tibial
Bladder
Brain - Amygdala
Brain - Anterior cingulate cortex
Brain - Caudate
Brain - Cerebellar Hemisphere
Brain - Cerebellum
Brain - Cortex
Brain - Frontal Cortex
Brain - Hippocampus
Brain - Hypothalamus
Brain - Nucleus accumbens
Brain - Putamen
Brain - Spinal cord
Brain - Substantia nigra
Breast - Mammary Tissue
Cells - Cultured fibroblasts
ls - EBV-transformed lymphocytes
Cervix - Ectocervix
Cervix - Endocervix
Colon - Sigmoid
Colon - Transverse
gus - Gastroesophageal Junction
Esophagus - Mucosa
Esophagus - Muscularis
Fallopian Tube
Heart - Atrial Appendage
Heart - Left Ventricle
Kidney - Cortex
Liver
Lung
Minor Salivary Gland
Muscle - Skeletal
Nerve - Tibial
Ovary
Pancreas
Pituitary
Prostate
Skin - Not Sun Exposed
Skin - Sun Exposed
Small Intestine - Terminal Ileum
Spleen
Stomach
Testis
Thyroid
Uterus
Vagina
Whole Blood

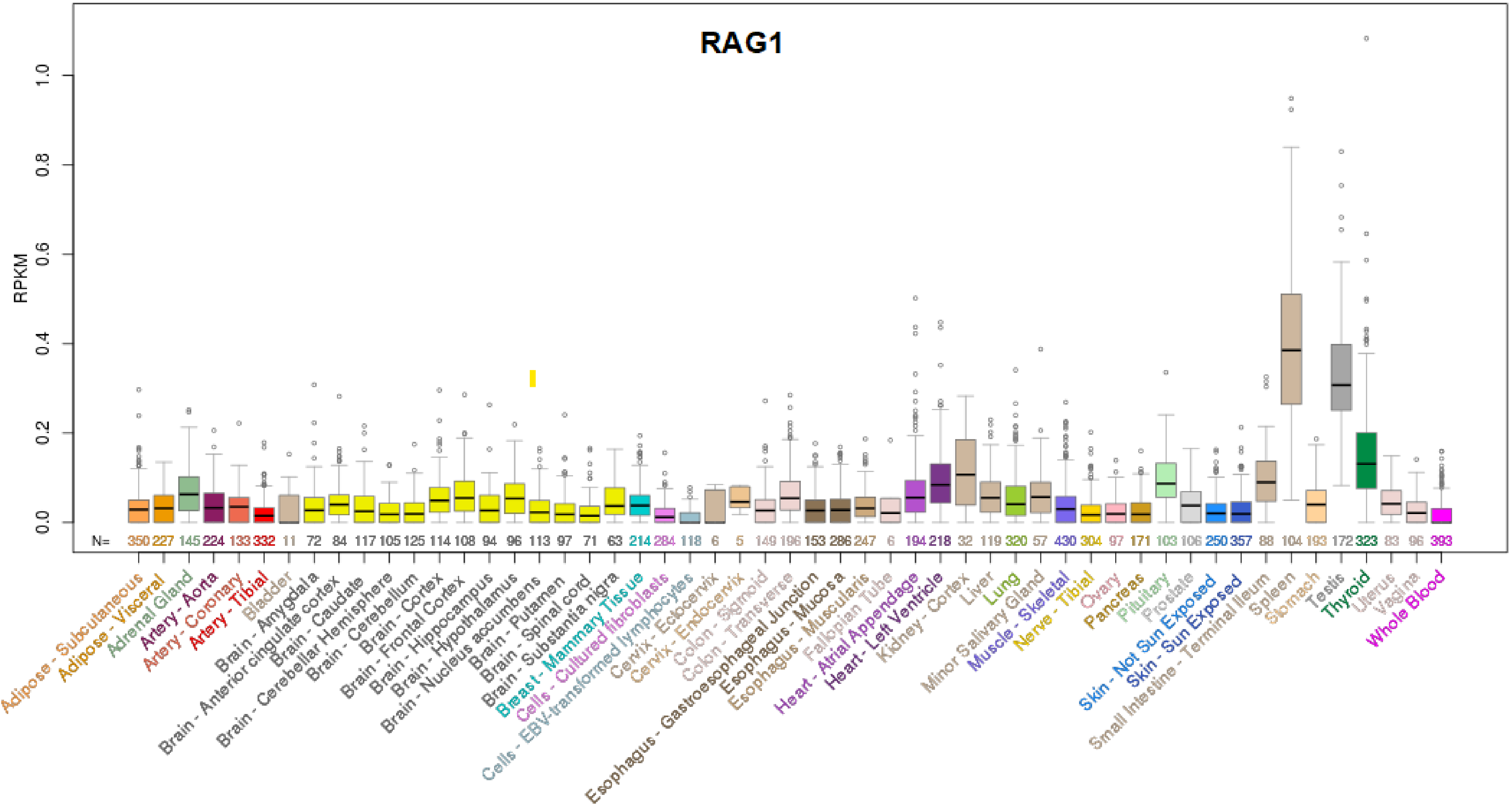
RAG1
RPKM
0.0
0.2
0.4
0.6
0.8
1.0
N=
350 227 145 224 133 332 11 72 84 117 105 125 114 108 94 96 113 97 71 63 214 284 118 6 5 149 196 153 286 247 6 194 218 32 119 320 57 430 304 97 171 103 106 250 357 88 104 193 172 323 83 96 393
Adipose - Subcutaneous
Adipose - Visceral
Adrenal Gland
Artery - Aorta
Artery - Coronary
Artery - Tibial
Bladder
Brain - Amygdala
Brain - Anterior cingulate cortex
Brain - Caudate
Brain - Cerebellar Hemisphere
Brain - Cerebellum
Brain - Cortex
Brain - Frontal Cortex
Brain - Hippocampus
Brain - Hypothalamus
Brain - Nucleus accumbens
Brain - Putamen
Brain - Spinal cord
Brain - Substantia nigra
Breast - Mammary Tissue
Cells - Cultured fibroblasts
Cells - EBV-transformed lymphocytes
Cervix - Ectocervix
Cervix - Endocervix
Colon - Sigmoid
Colon - Transverse
Esophagus - Gastroesophageal Junction
Esophagus - Mucosa
Esophagus - Muscularis
Fallopian Tube
Heart - Atrial Appendage
Heart - Left Ventricle
Kidney - Cortex
Liver
Lung
Minor Salivary Gland
Muscle - Skeletal
Nerve - Tibial
Ovary
Pancreas
Pituitary
Prostate
Skin - Not Sun Exposed
Skin - Sun Exposed
Small Intestine - Terminal Ileum
Spleen
Stomach
Testis
Thyroid
Uterus
Vagina
Whole Blood

Supplemental Figure S3

Legend. Wheel plots showing single cell expression data from the developing mouse brain (http://mousebrain.org). The expression pattern is displayed as a UMAP clusters of2 different cell types determined by the expression pattern of cell specific markers. Slides 2 and 3 show the different cell types making up the clusters, while slide 4 shows the brain regions covered by the clusters. Slide 5 shows an example of a gene that is expressed in a cell type-specific manner, which was used to create the gene clusters. Slides 6-16 show the expression pattern at a common developmental stage. The box on the left of each wheel pot is the relative expression level within the different cell types.

Cell types

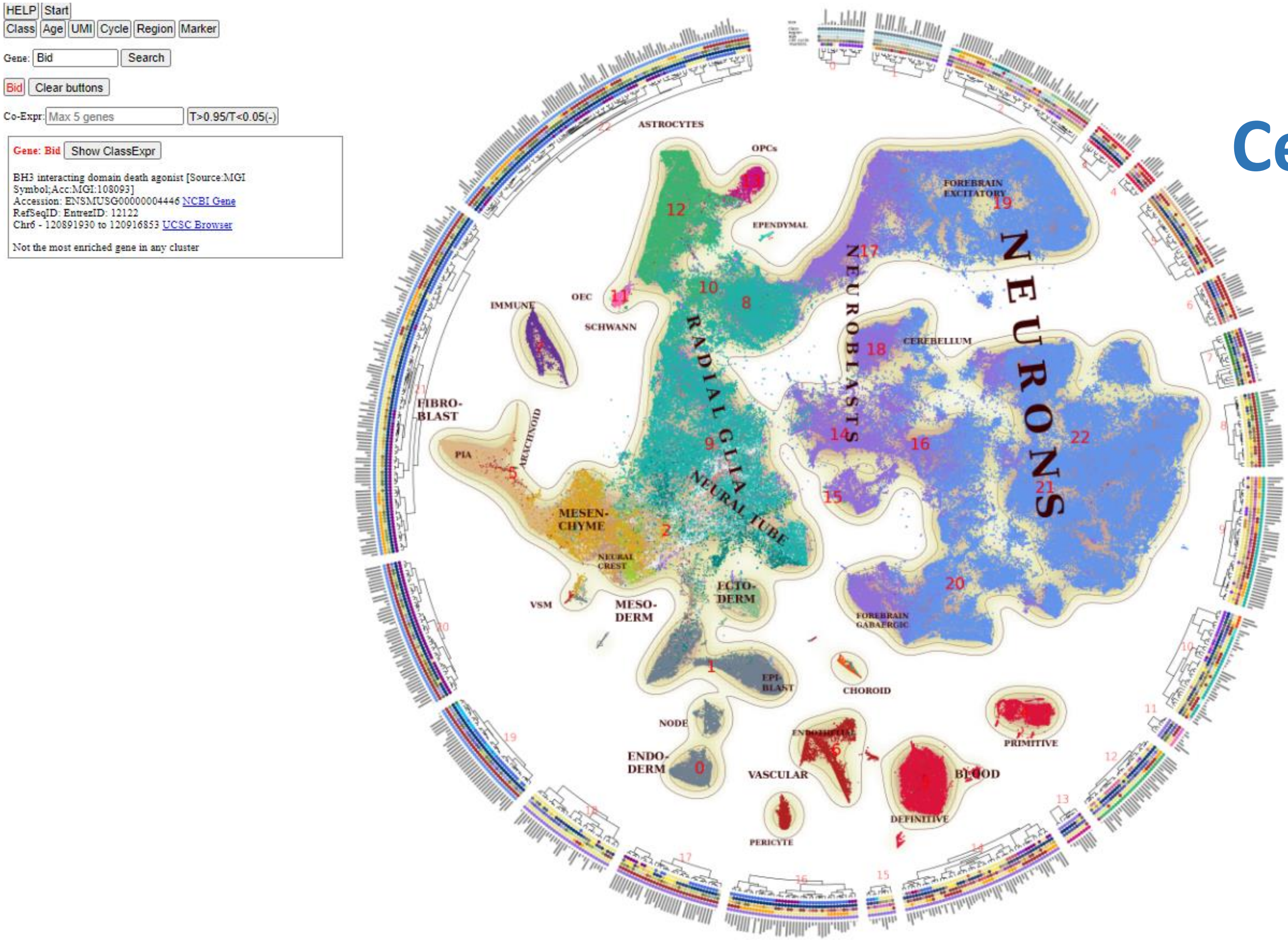
HELP
Start
Class
Age
UMI
Cycle
Region
Marker
Gene: Bid
Search
Bid
Clear buttons
Co-Expr: Max 5 genes
T>0.95/T<0.05(-)
Gene: Bid
Show ClassExpr
BH3 interacting domain death agonist [Source:MGI Symbol;Acc:MGI:108093]
Accession: ENSMUSG00000004446 NCBI Gene
RefSeqID: EntrezID: 12122
Chr6 - 120891930 to 120916853 UCSC Browser
Not the most enriched gene in any cluster
ASTROCYTES
OPCs
EPENDYMAL
FOREBRAIN EXCITATORY
NEURONS
NEUROBLASTS
RADIAL GLIA
NEURAL TUBE
OEC
SCHWANN
IMMUNE
CEREBELLUM
FIBRO-BLAST
PIA
ARACHNOID
MESEN-CHYME
NEURAL CREST
VSM
MESO-DERM
ECTO-DERM
FOREBRAIN GABAERGIC
EPI-BLAST
CHOROID
NODE
ENDO-DERM
VASCULAR
ENDOTHELIAL
PERICYTE
BLOOD
PRIMITIVE
DEFINITIVE

Class

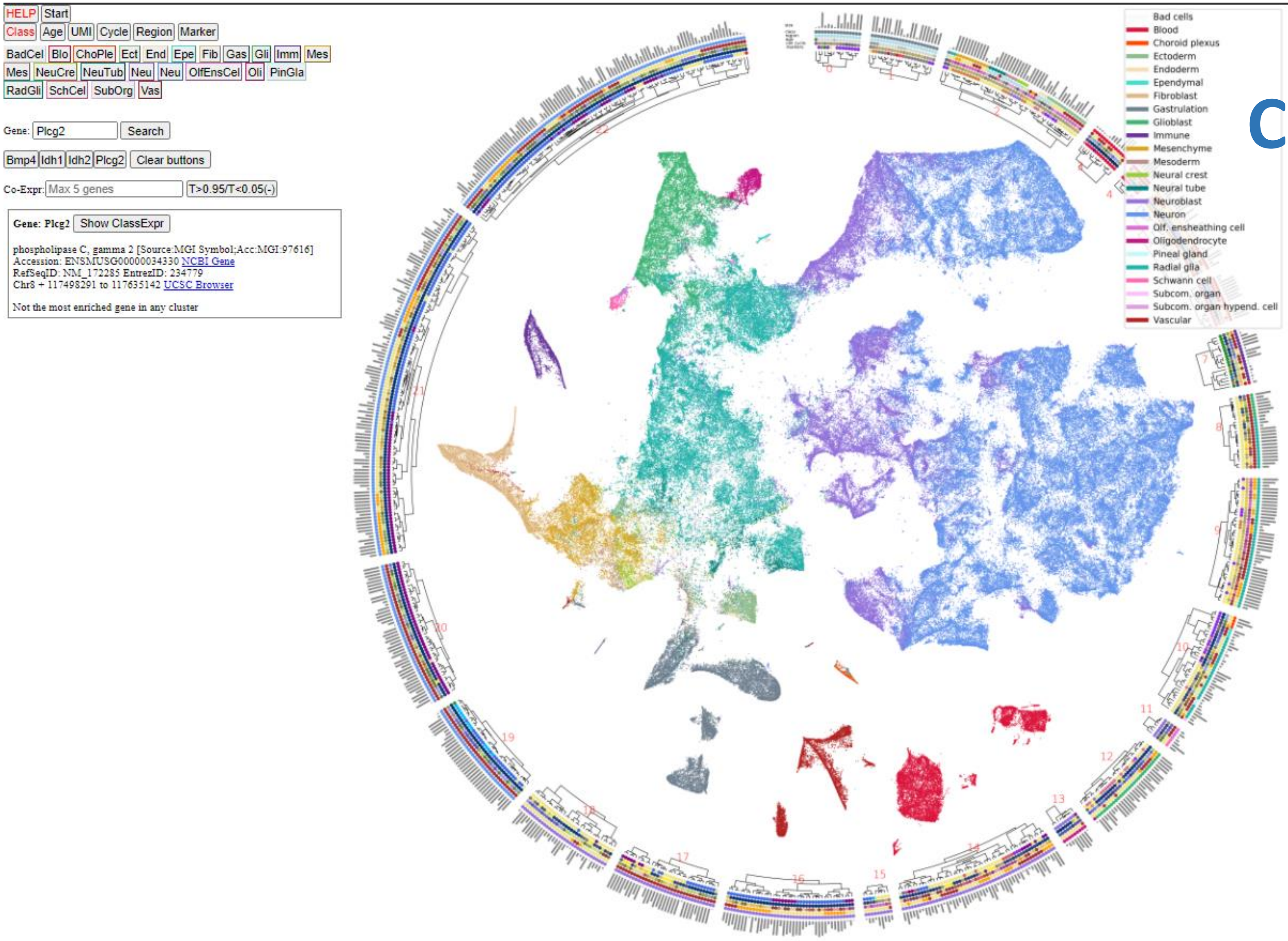
HELP Start
Class Age UMI Cycle Region Marker
BadCel Blo ChoPle Ect End Epe Fib Gas Gli Imm Mes
Mes NeuCre NeuTub Neu Neu OlfEnsCel Oli PinGla
RadGli SchCel SubOrg Vas
Gene: Plcg2 Search
Bmp4 Idh1 Idh2 Plcg2 Clear buttons
Co-Expr: Max 5 genes T>0.95/T<0.05(-)
Gene: Plcg2 Show ClassExpr
phospholipase C, gamma 2 [Source:MGI Symbol;Acc:MGI:97616]
Accession: ENSMUSG00000034330 NCBI Gene
RefSeqID: NM_172285 EntrezID: 234779
Chr8 + 117498291 to 117635142 UCSC Browser
Not the most enriched gene in any cluster
Bad cells
Blood
Choroid plexus
Ectoderm
Endoderm
Ependymal
Fibroblast
Gastrulation
Glioblast
Immune
Mesenchyme
Mesoderm
Neural crest
Neural tube
Neuroblast
Neuron
Olf. ensheathing cell
Oligodendrocyte
Pineal gland
Radial glia
Schwann cell
Subcom. organ
Subcom. organ hypend. cell
Vascular

Region

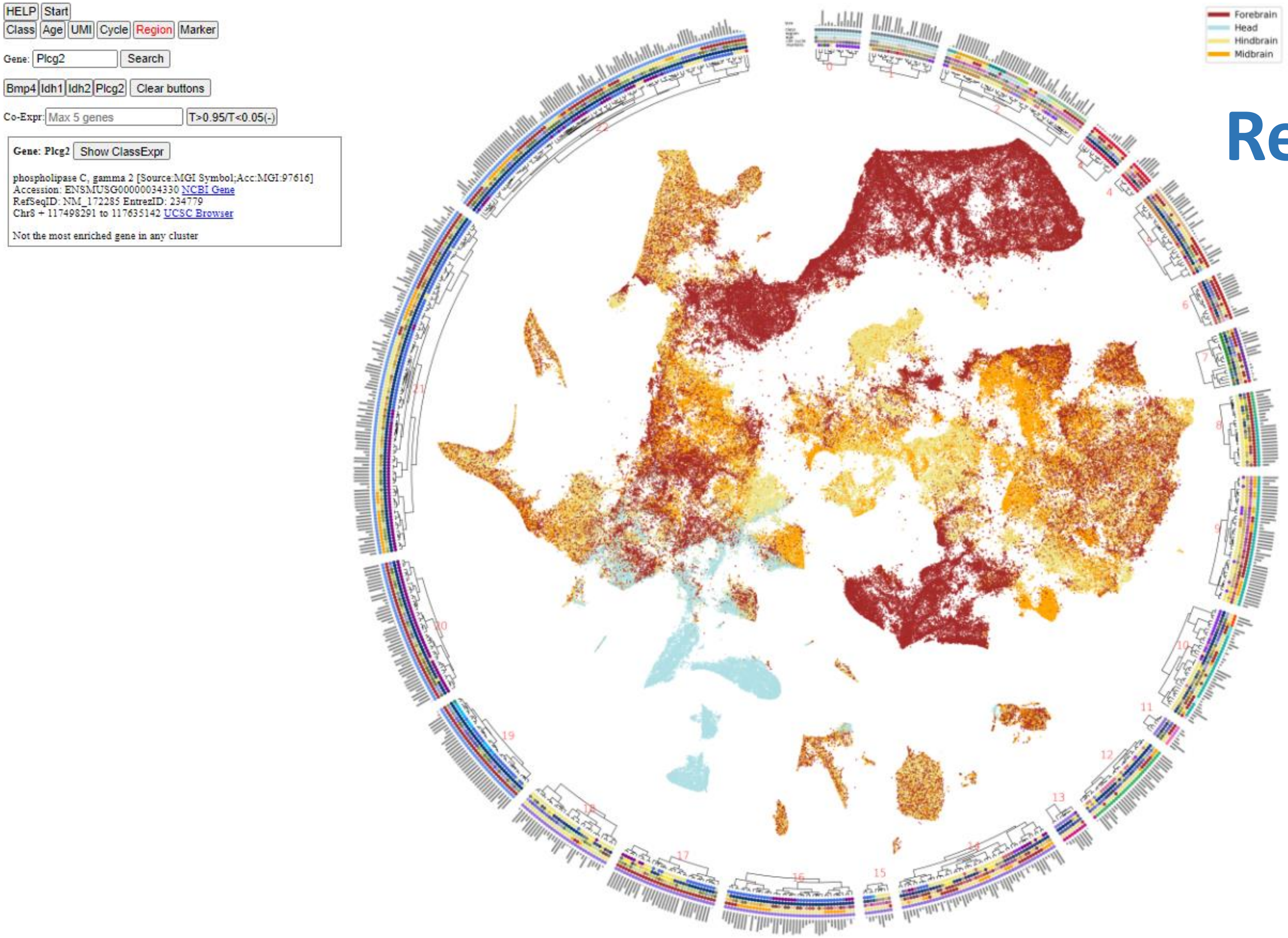
HELP Start
Class Age UMI Cycle Region Marker
Gene: Plcg2 Search
Bmp4 Idh1 Idh2 Plcg2 Clear buttons
Co-Expr: Max 5 genes T>0.95/T<0.05(-)
Gene: Plcg2 Show ClassExpr
phospholipase C, gamma 2 [Source:MGI Symbol;Acc:MGI:97616]
Accession: ENSMUSG00000034330 NCBI Gene
RefSeqID: NM_172285 EntrezID: 234779
Chr8 + 117498291 to 117635142 UCSC Browser
Not the most enriched gene in any cluster
Forebrain
Head
Hindbrain
Midbrain

Marker

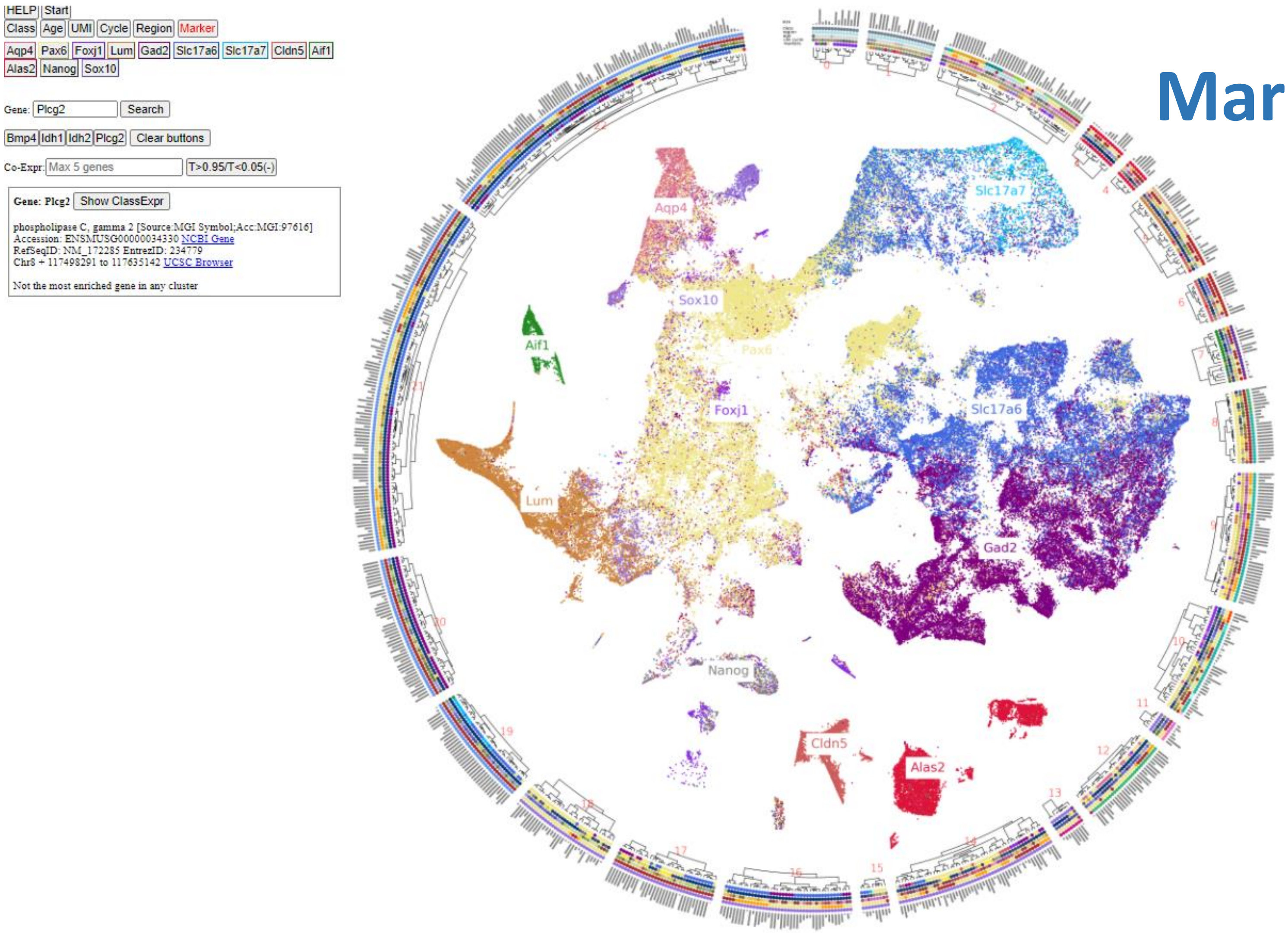

HELP
Start
Class
Age
UMI
Cycle
Region
Marker
Aqp4
Pax6
Foxj1
Lum
Gad2
Slc17a6
Slc17a7
Cldn5
Aif1
Alas2
Nanog
Sox10
Gene: Plcg2
Search
Bmp4
Idh1
Idh2
Plcg2
Clear buttons
Co-Expr: Max 5 genes
T>0.95/T<0.05(-)
Gene: Plcg2
Show ClassExpr
phospholipase C, gamma 2 [Source:MGI Symbol;Acc:MGI:97616]
Accession: ENSMUSG00000034330 NCBI Gene
RefSeqID: NM_172285 EntrezID: 234779
Chr8 + 117498291 to 117635142 UCSC Browser
Not the most enriched gene in any cluster
Aqp4
Slc17a7
Sox10
Pax6
Aif1
Foxj1
Slc17a6
Lum
Gad2
Nanog
Cldn5
Alas2

HELP Start

Class Age UMI Cycle Region Marker

Gene: Shank3 Search

Shank3 Clear buttons

Co-Expr: Max 5 genes T>0.95/T<0.05(-)

Gene: Shank3 Hide ClassExpr

Bad cells	0.00
Blood	0.01
Choroid plexus	0.21
Ectoderm	0.01
Endoderm	0.01
Ependymal	0.23
Fibroblast	0.04
Gastrulation	0.03
Glioblast	0.02
Immune	0.01
Mesenchyme	0.03
Mesoderm	0.03
Neural crest	0.02
Neural tube	0.03
Neuroblast	0.04
Neuron	0.10
Olfactory ensheathing cell	0.02
Oligodendrocyte	0.04
Pineal gland	0.04
Radial glia	0.03
Schwann cell	0.01
Subcommissural organ	0.13
Vascular	0.60

SH3 and multiple ankyrin repeat domains 3 [Source:MGI Symbol;Acc:MGI:1930016]
Accession: ENSMUSG00000022623 NCBI Gene
RefSeqID: EntrezID: 58234
Chr15 + 89499623 to 89560261 UCSC Browser

Not the most enriched gene in any cluster

< **Cluster: 302** > **AstroMix5. 451 cells.**

Class:Glioblast Subclass: Mixed region astrocytes
Mostly Hindbrain.

Show Distribution

AutoAnnotations:

@AEP @IEG AC-NFB Astro2 NbFor5 Rgl Rgl2 RglF2 RglH1 RglM2

Markers:

Agt Il33 Slc7a10 Cldn10 Slc6a9 Cyp2j9

Enriched TFs:

Dbx2 Olig1 Etv4 Rxrg Klf9 Klf15 Hes5 Bhlhe40

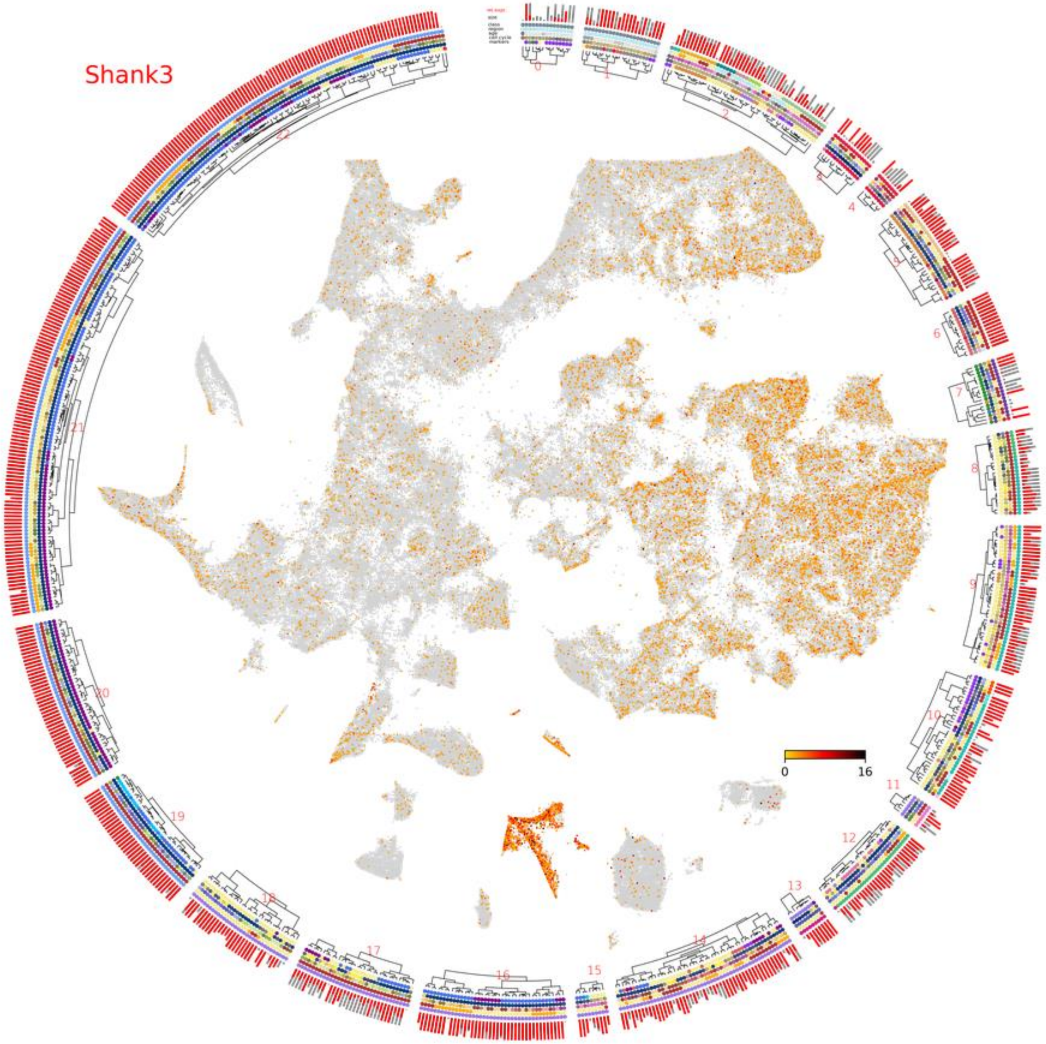

HELP | Start

Class | Age | UMI | Cycle | Region | Marker

Gene: Cacna1b Search

Ppm1d | Sgce | Cacna1b | Clear buttons

Co-Expr: CACNA1B T>0.95/T<0.05(-)

Gene: Cacna1b Hide ClassExpr

Bad cells	0.00
Blood	0.01
Choroid plexus	0.03
Ectoderm	0.24
Endoderm	0.10
Ependymal	0.08
Fibroblast	0.02
Gastrulation	0.04
Glioblast	0.01
Immune	0.03
Mesenchyme	0.01
Mesoderm	0.01
Neural crest	0.03
Neural tube	0.07
Neuroblast	0.13
Neuron	0.37
Olfactory ensheathing cell	0.06
Oligodendrocyte	0.08
Pineal gland	0.01
Radial glia	0.02
Schwann cell	0.01
Subcommissural organ	0.02
Vascular	0.03

calcium channel, voltage-dependent, N type, alpha 1B subunit
[Source:MGI Symbol;Acc:MGI:88296]
Accession: ENSMUSG00000004113 NCBI Gene
RefSeqID: EntrezID: 12287
Chr2 - 24603887 to 24763152 UCSC Browser

Not the most enriched gene in any cluster

< **Cluster: 566** > **Neur566. 338 cells.**

Class:Neuron Subclass: Forebrain GABAergic
Mostly ForebrainVentral.
Show Distribution

AutoAnnotations:
@GABA | Dlx | IntHc1 | MGEInt | NbFor5 | NbInh1

Markers:
Sst | Fam135b | Fam222a | Sox6 | Gm45881 | Dlgap1

Enriched TFs:
Lhx6 | Arx | Dlx2 | Dlx5 | Dlx1 | Sox6 | Mafb | Sp9

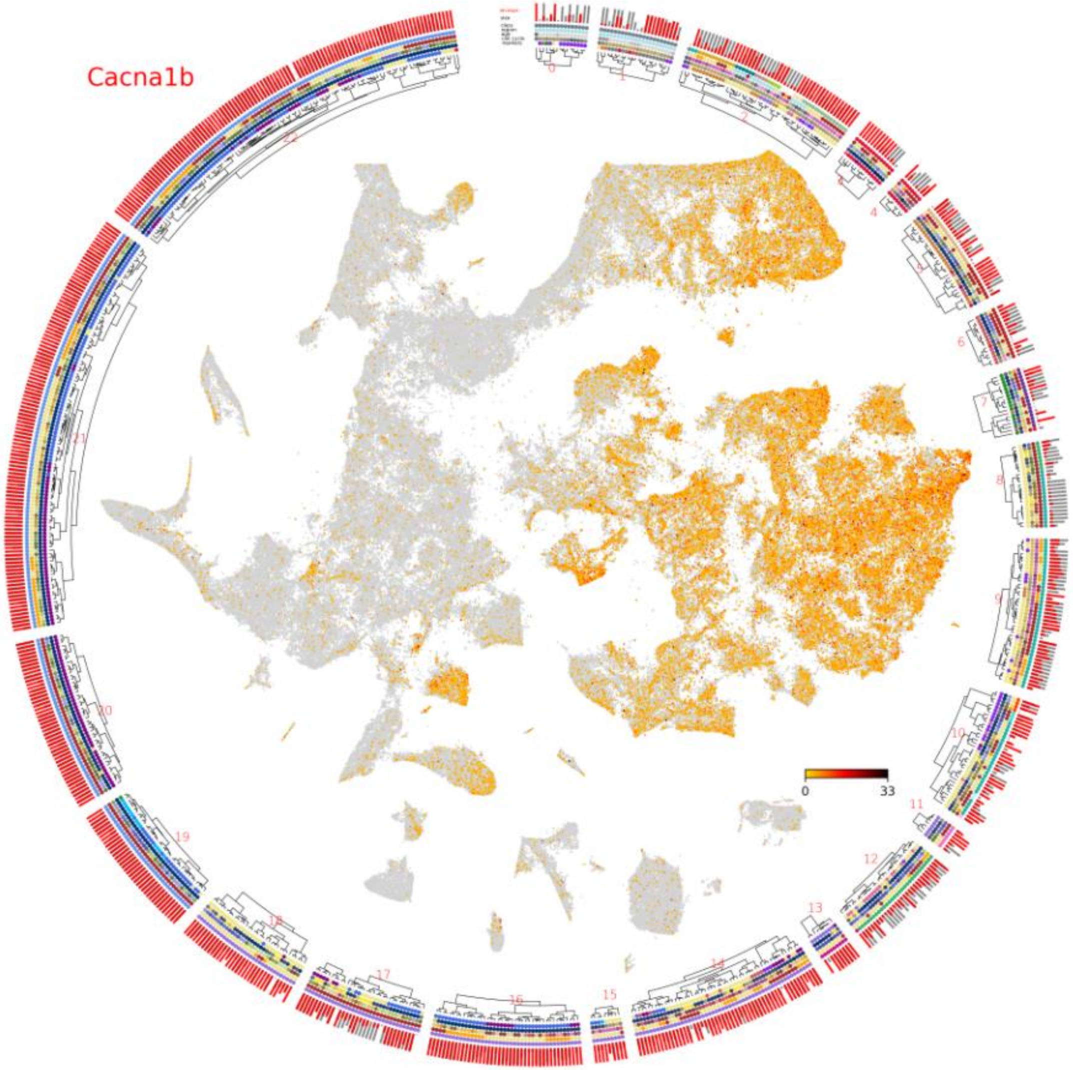

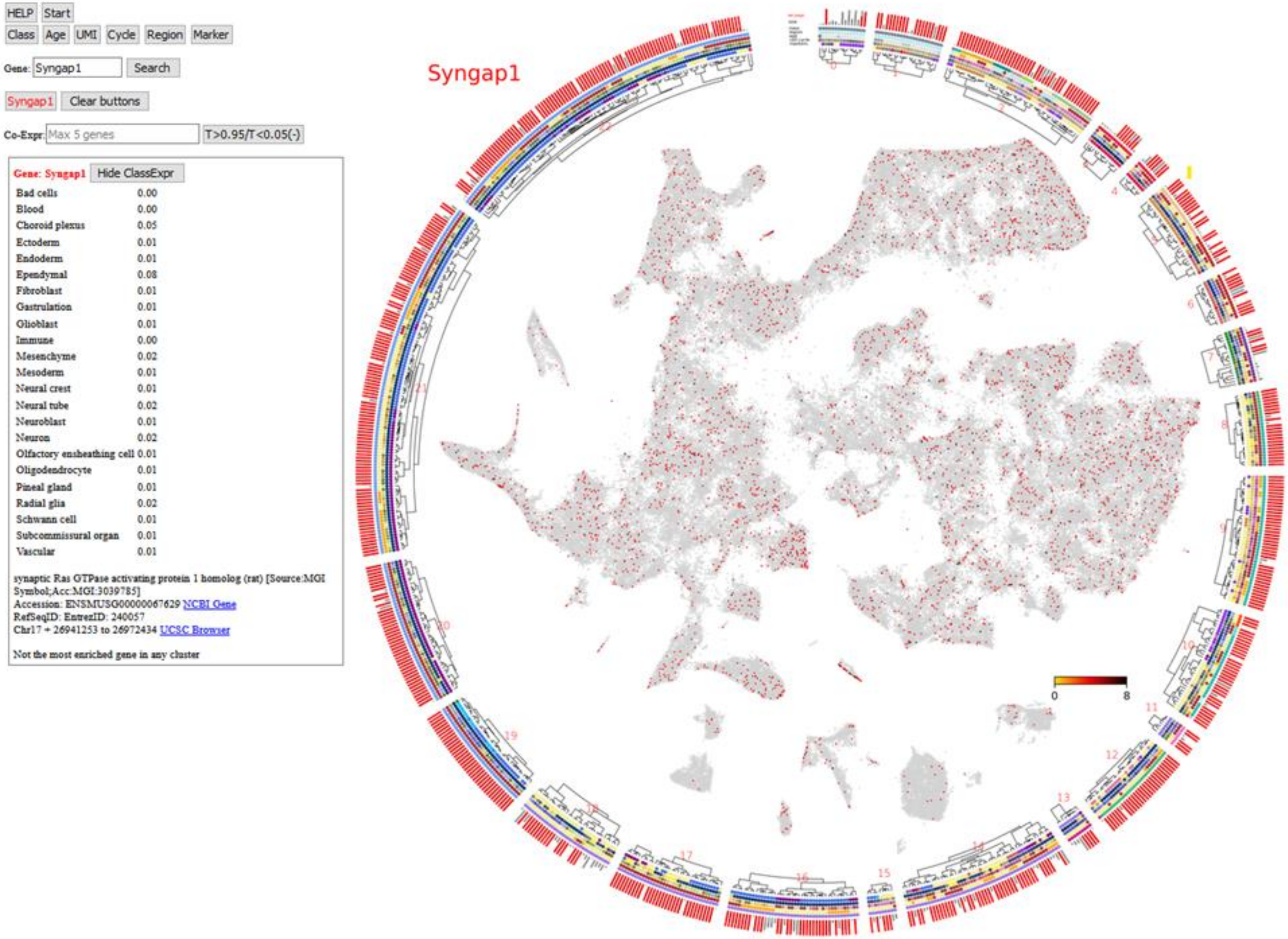

HELP Start
Class Age UMI Cycle Region Marker
Gene: Syngap1 Search
Syngap1 Clear buttons
Co-Expr: Max 5 genes T>0.95/T<0.05(-)
Gene: Syngap1 Hide ClassExpr
Bad cells 0.00
Blood 0.00
Choroid plexus 0.05
Ectoderm 0.01
Endoderm 0.01
Ependymal 0.08
Fibroblast 0.01
Gastrulation 0.01
Glioblast 0.01
Immune 0.00
Mesenchyme 0.02
Mesoderm 0.01
Neural crest 0.01
Neural tube 0.02
Neuroblast 0.01
Neuron 0.02
Olfactory ensheathing cell 0.01
Oligodendrocyte 0.01
Pineal gland 0.01
Radial glia 0.02
Schwann cell 0.01
Subcommissural organ 0.01
Vascular 0.01
synaptic Ras GTPase activating protein 1 homolog (rat) [Source:MGI Symbol;Acc:MGI:3039785]
Accession: ENSMUSG00000067629 NCBI Gene
RefSeqID: EntrezID: 240057
Chr17 + 26941253 to 26972434 UCSC Browser
Not the most enriched gene in any cluster
Syngap1
0
8

HELP | Start

Class | Age | UMI | Cycle | Region | Marker

Gene: Sgce Search

Ppm1d | Sgce | Clear buttons

Co-Expr: Max 5 genes T>0.95/T<0.05(-)

Gene: Sgce Hide ClassExpr

Bad cells	0.14
Blood	0.03
Choroid plexus	0.58
Ectoderm	0.38
Endoderm	0.56
Ependymal	0.87
Fibroblast	0.89
Gastrulation	0.30
Glioblast	0.57
Immune	0.32
Mesenchyme	0.55
Mesoderm	0.53
Neural crest	0.34
Neural tube	0.50
Neuroblast	0.37
Neuron	0.32
Olfactory ensheathing cell	0.46
Oligodendrocyte	0.44
Pineal gland	0.41
Radial glia	0.60
Schwann cell	0.46
Subcommissural organ	0.45
Vascular	0.78

sarcoglycan, epsilon [Source:MGI Symbol;Acc:MGI:1329042]
Accession: ENSMUSG00000004631 NCBI Gene
RefSeqID: EntrezID: -
Chr6 - 4674350 to 4747207 UCSC Browser

Not the most enriched gene in any cluster

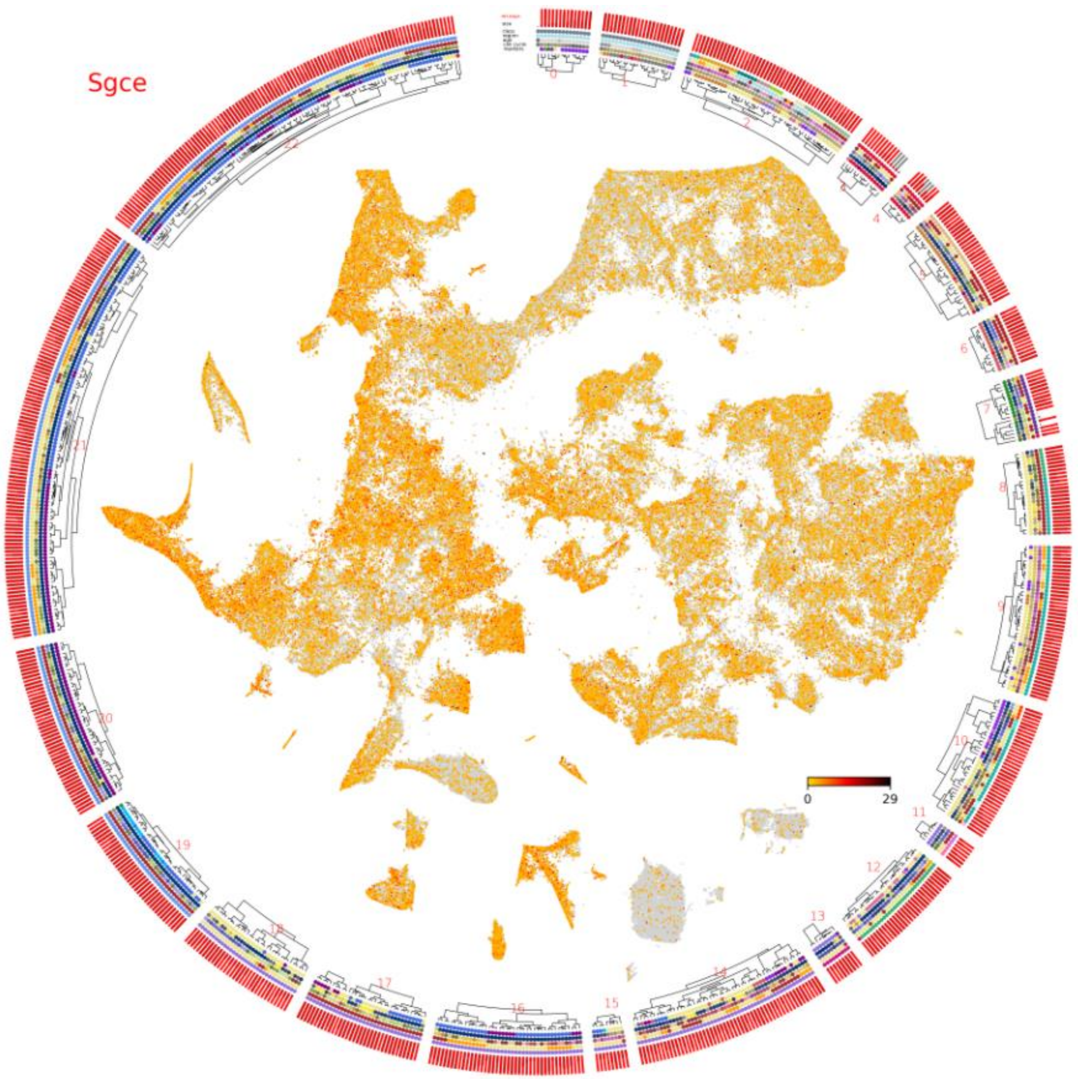

HELP Start

Class Age UMI Cycle Region Marker

Gene: Grin2a Search

Syngap1 Grin2a Clear buttons

Co-Expr: Max 5 genes T>0.95/T<0.05(-)

Gene: Grin2a Hide ClassExpr

Bad cells	0.00
Blood	0.01
Choroid plexus	0.01
Ectoderm	0.01
Endoderm	0.00
Ependymal	0.28
Fibroblast	0.01
Gastrulation	0.01
Glioblast	0.05
Immune	0.01
Mesenchyme	0.03
Mesoderm	0.01
Neural crest	0.01
Neural tube	0.05
Neuroblast	0.04
Neuron	0.12
Olfactory ensheathing cell	0.01
Oligodendrocyte	0.02
Pineal gland	0.00
Radial glia	0.05
Schwann cell	0.02
Subcommissural organ	0.01
Vascular	0.09

glutamate receptor, ionotropic, NMDA2A (epsilon 1) [Source:MGI Symbol;Acc:MGI:95820]
Accession: ENSMUSG00000059003 NCBI Gene
RefSeqID: EntrezID: -
Chr16 - 9567898 to 9995560 UCSC Browser

Not the most enriched gene in any cluster

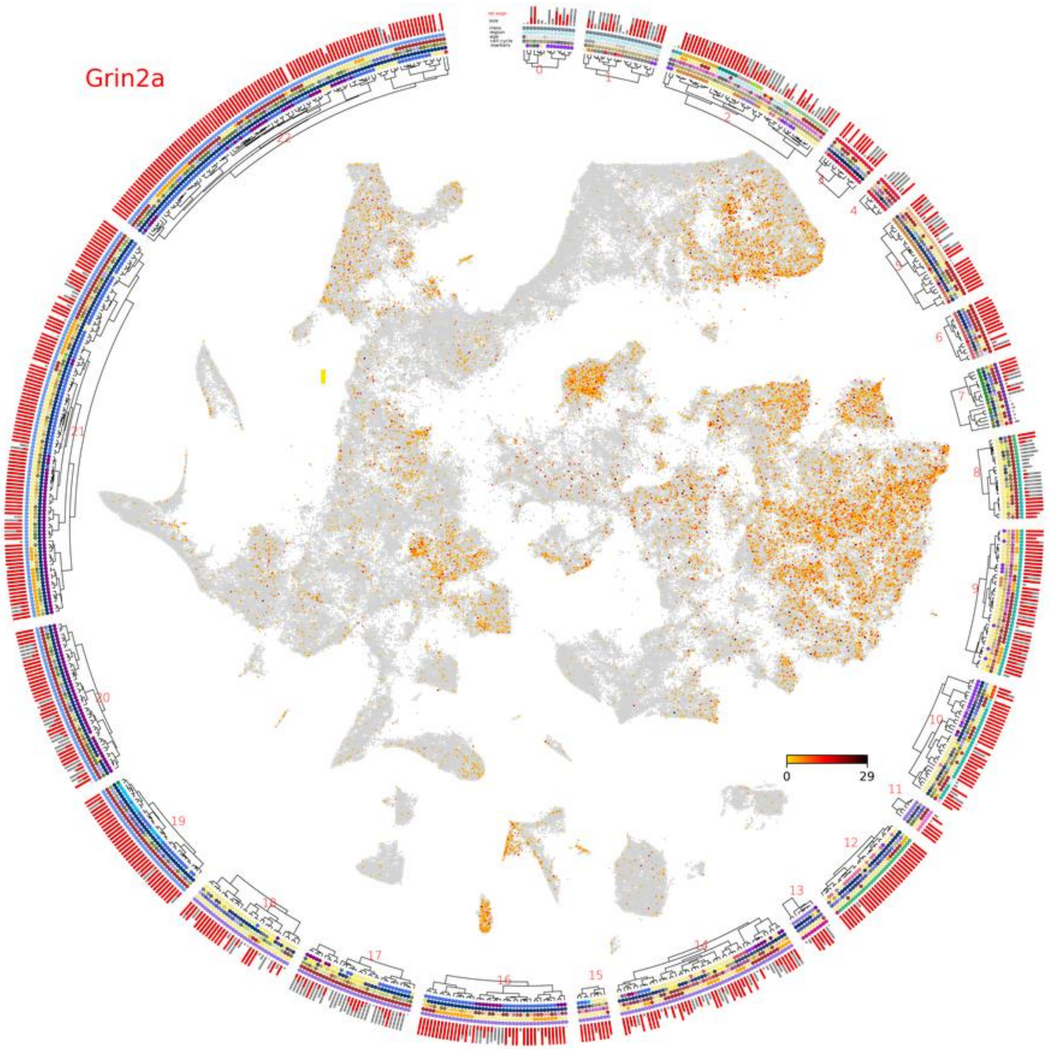

HELP Start

Class Age UMI Cycle Region Marker

Gene: Nlrc4 Search

Grin2a Nlrc4 Clear buttons

Co-Expr: Max 5 genes T>0.95/T<0.05(-)

Gene: Nlrc4 Hide ClassExpr

Bad cells	0.01
Blood	0.01
Choroid plexus	0.03
Ectoderm	0.03
Endoderm	0.02
Ependymal	0.06
Fibroblast	0.02
Gastrulation	0.03
Glioblast	0.01
Immune	0.11
Mesenchyme	0.03
Mesoderm	0.02
Neural crest	0.02
Neural tube	0.02
Neuroblast	0.01
Neuron	0.01
Olfactory ensheathing cell	0.02
Oligodendrocyte	0.01
Pineal gland	0.01
Radial glia	0.02
Schwann cell	0.01
Subcommissural organ	0.00
Vascular	0.02

NLR family, CARD domain containing 4 [Source:MGI Symbol;Acc:MGI:3036243]
Accession: ENSMUSG00000039193 NCBI Gene
RefSeqID: EntrezID: 268973
Chr17 - 74426295 to 74459108 UCSC Browser

Not the most enriched gene in any cluster

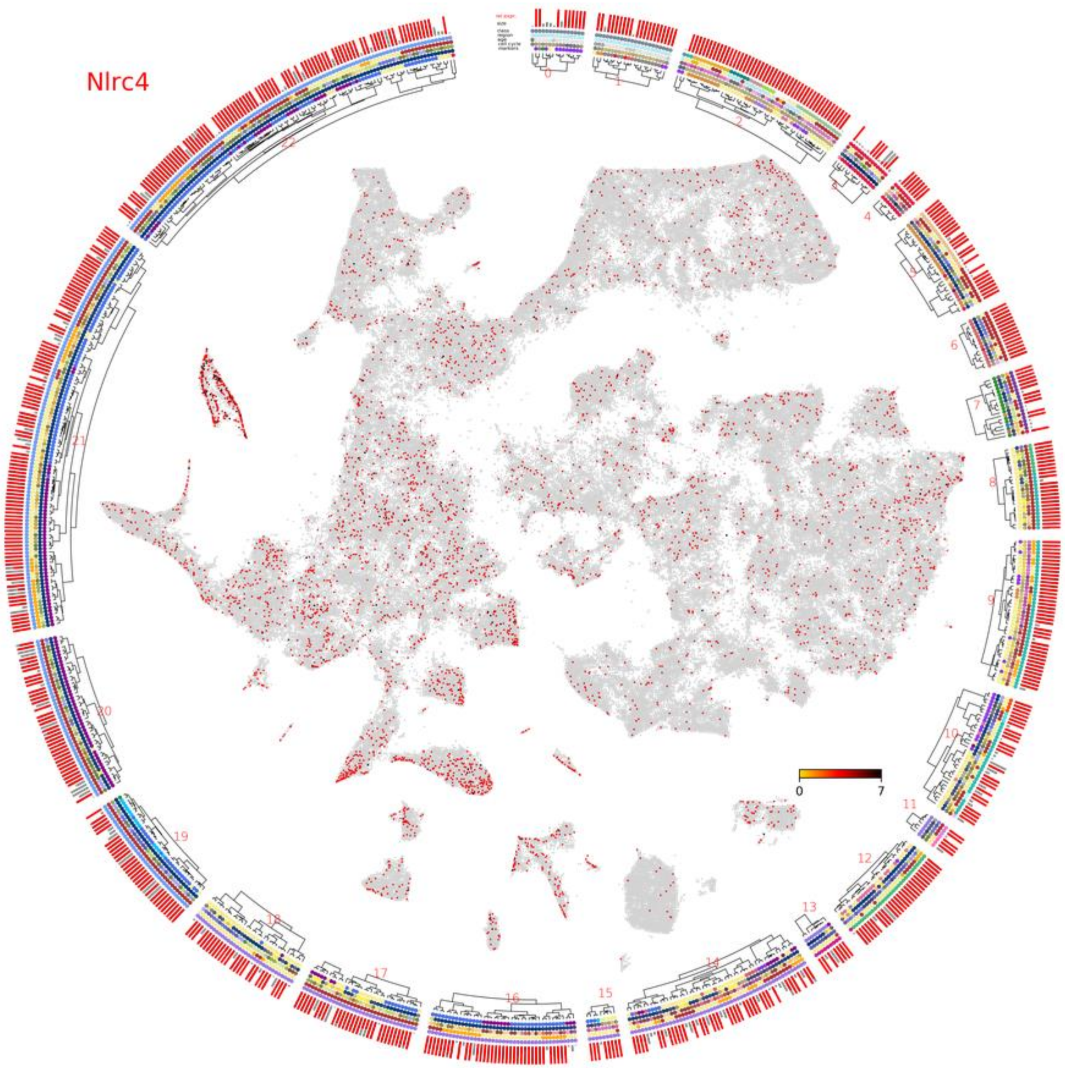

HELP Start

Class Age UMI Cycle Region Marker

Gene: Ppm1d Search

Ppm1d Clear buttons

Co-Expr: Max 5 genes T>0.95/T<0.05(-)

Gene: Ppm1d Hide ClassExpr

Bad cells	0.16
Blood	0.16
Choroid plexus	0.23
Ectoderm	0.28
Endoderm	0.29
Ependymal	0.23
Fibroblast	0.15
Gastrulation	0.31
Glioblast	0.20
Immune	0.11
Mesenchyme	0.33
Mesoderm	0.31
Neural crest	0.40
Neural tube	0.40
Neuroblast	0.28
Neuron	0.18
Olfactory ensheathing cell	0.23
Oligodendrocyte	0.19
Pineal gland	0.22
Radial glia	0.37
Schwann cell	0.19
Subcommissural organ	0.16
Vascular	0.23

protein phosphatase 1D magnesium-dependent, delta isoform
[Source:MGI Symbol;Acc:MGI:1858214]
Accession: ENSMUSG00000020525 NCBI Gene
RefSeqID: EntrezID: 53892
Chr11 + 85311244 to 85347066 UCSC Browser

Not the most enriched gene in any cluster

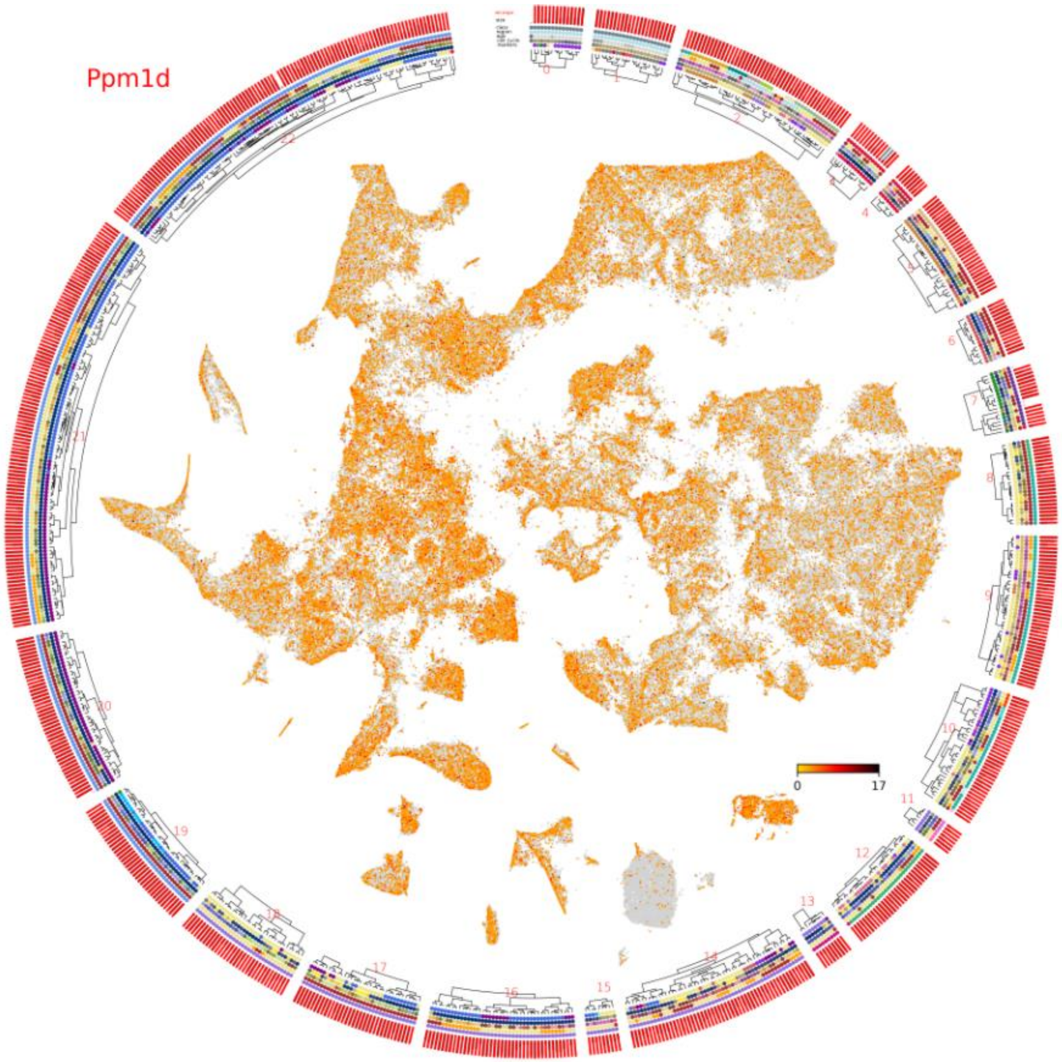

HELP | Start

Class | Age | UMI | Cycle | Region | Marker

Gene: Plcg2 | Search

Grin2a | Nlrc4 | Plcg2 | Clear buttons

Co-Expr: Max 5 genes | T>0.95/T<0.05(-)

Gene: Plcg2 | Hide ClassExpr

Bad cells	0.02
Blood	0.01
Choroid plexus	0.02
Ectoderm	0.07
Endoderm	0.05
Ependymal	0.03
Fibroblast	0.01
Gastrulation	0.09
Glioblast	0.00
Immune	0.43
Mesenchyme	0.12
Mesoderm	0.03
Neural crest	0.72
Neural tube	0.05
Neuroblast	0.00
Neuron	0.00
Olfactory ensheathing cell	0.00
Oligodendrocyte	0.04
Pineal gland	0.00
Radial glia	0.01
Schwann cell	0.00
Subcommissural organ	0.00
Vascular	0.05

phospholipase C, gamma 2 [Source:MGI Symbol;Acc:MGI:97616]
Accession: ENSMUSG00000034330 NCBI Gene
RefSeqID: NM_172285 EntrezID: 234779
Chr8 + 117498291 to 117635142 UCSC Browser

Not the most enriched gene in any cluster

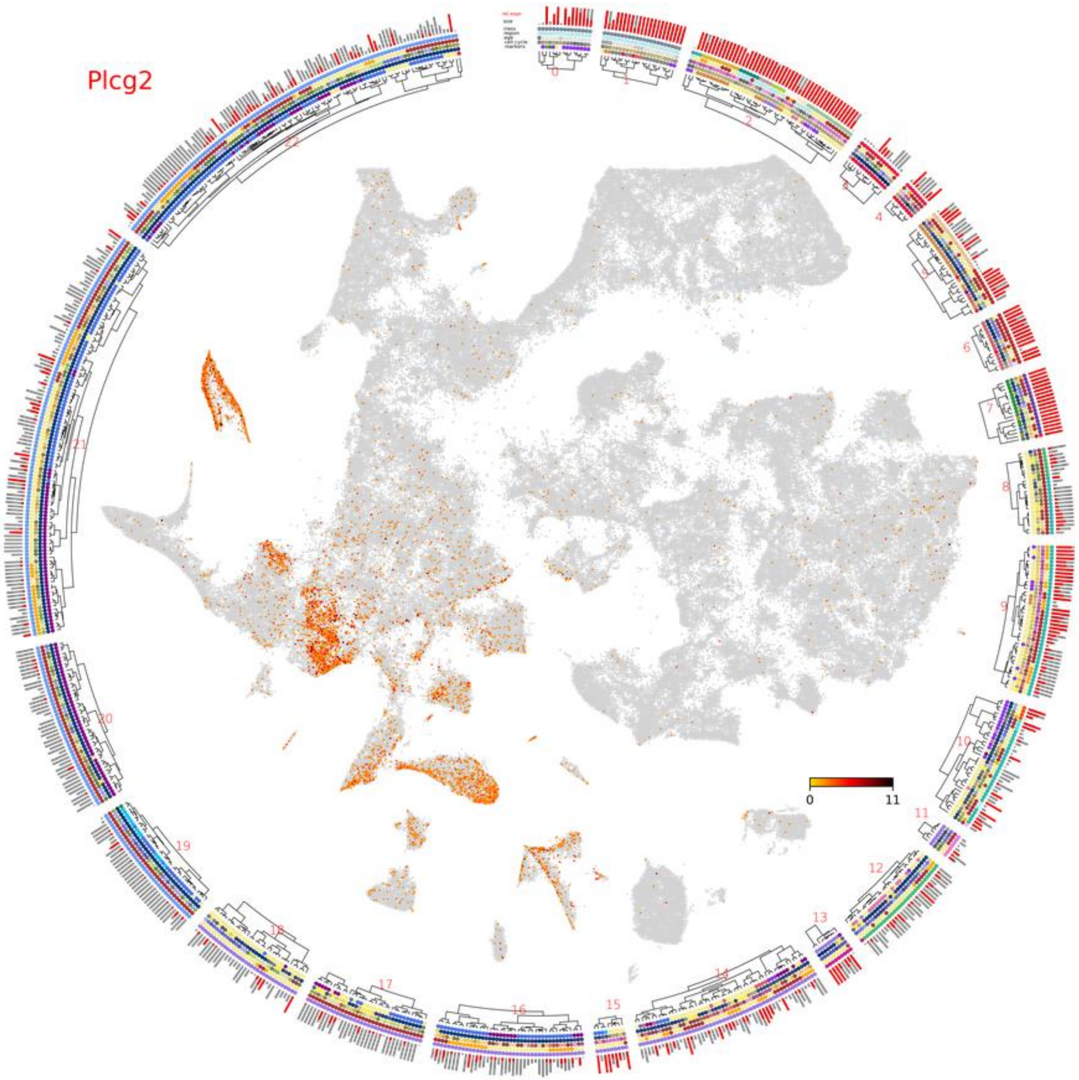

HELP Start

Class Age UMI Cycle Region Marker

Gene: Chek2 Search

Grin2a Nlrc4 Plcg2 Chek2 Clear buttons

Co-Expr: Max 5 genes T>0.95/T<0.05(-)

Gene: Chek2 Hide ClassExpr

Bad cells	0.09
Blood	0.06
Choroid plexus	0.04
Ectoderm	0.21
Endoderm	0.22
Ependymal	1.09
Fibroblast	0.04
Gastrulation	0.26
Glioblast	0.09
Immune	0.10
Mesenchyme	0.23
Mesoderm	0.23
Neural crest	0.31
Neural tube	0.36
Neuroblast	0.07
Neuron	0.00
Olfactory ensheathing cell	0.04
Oligodendrocyte	0.06
Pineal gland	0.11
Radial glia	0.24
Schwann cell	0.05
Subcommissural organ	0.49
Vascular	0.13

checkpoint kinase 2 [Source:MGI Symbol;Acc:MGI:1355321]
Accession: ENSMUSG00000029521 NCBI Gene
RefSeqID: EntrezID: 50883
Chr5 + 110839979 to 110874145 UCSC Browser

Not the most enriched gene in any cluster

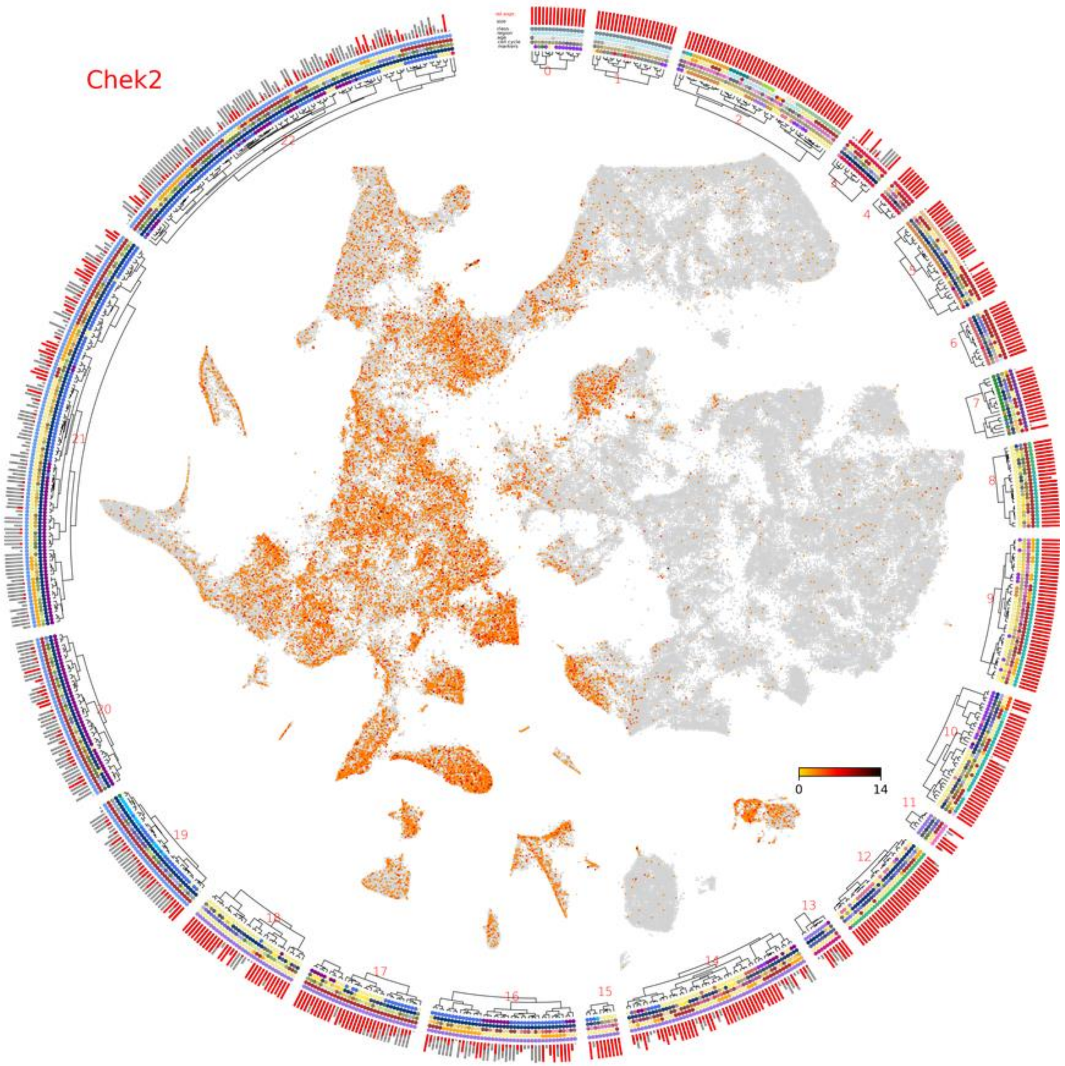

HELP Start

Class Age UMI Cycle Region Marker

Gene: Rag1 Search

Rag1 Clear buttons

Co-Expr: Max 5 genes T>0.95/T<0.05(-)

Gene: Rag1 Hide ClassExpr

Bad cells	0.00
Blood	0.00
Choroid plexus	0.00
Ectoderm	0.00
Endoderm	0.00
Ependymal	0.00
Fibroblast	0.00
Gastrulation	0.00
Glioblast	0.00
Immune	0.00
Mesenchyme	0.00
Mesoderm	0.00
Neural crest	0.00
Neural tube	0.00
Neuroblast	0.00
Neuron	0.00
Olfactory ensheathing cell	0.00
Oligodendrocyte	0.00
Pineal gland	0.00
Radial glia	0.00
Schwann cell	0.00
Subcommissural organ	0.00
Vascular	0.00

recombination activating 1 [Source:MGI Symbol;Acc:MGI:97848]
Accession: ENSMUSG00000061311 NCBI Gene
RefSeqID: NM_009019 EntrezID: 19373
Chr2 - 101638282 to 101649501 UCSC Browser

Not the most enriched gene in any cluster

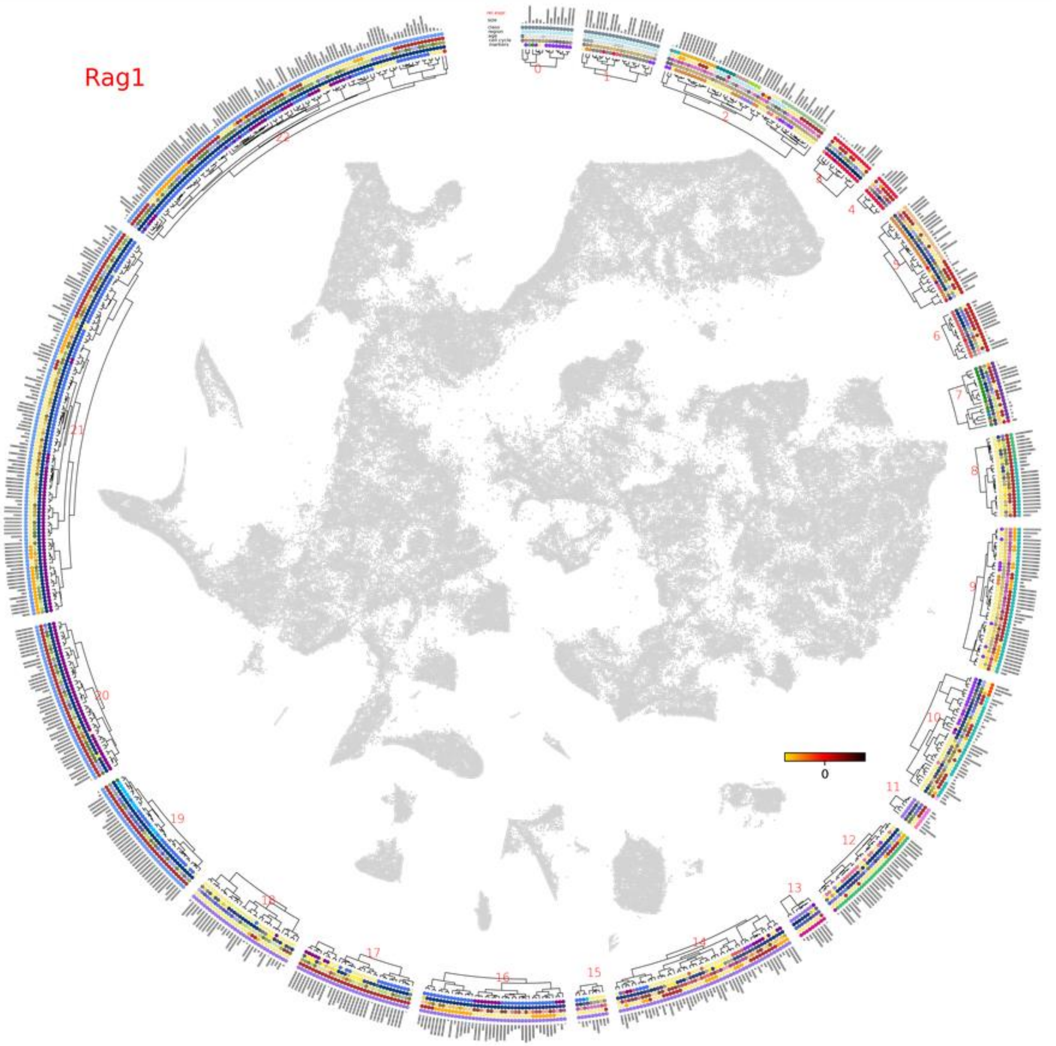

Table S1: abbreviation key to Figure 4 (mouse adolescent brain single cell RNA-seq

1	TEGLU1 <http://mousebrain.org/celltypes/TEGLU1>	Excitatory neurons, cerebral cortex
2	TEGLU3 <http://mousebrain.org/celltypes/TEGLU3>	Excitatory neurons, cerebral cortex
3	TEGLU2 <http://mousebrain.org/celltypes/TEGLU2>	Excitatory neurons, cerebral cortex
4	TEGLU20 <http://mousebrain.org/celltypes/TEGLU20>	Excitatory neurons, cerebral cortex
5	TEGLU11 <http://mousebrain.org/celltypes/TEGLU11>	Excitatory neurons, cerebral cortex
6	TEGLU12 http://mousebrain.org/celltypes/TEGLU12	Excitatory neurons, cerebral cortex
7	TEGLU10 <http://mousebrain.org/celltypes/TEGLU10>	Excitatory neurons, cerebral cortex
8	TEGLU9 <http://mousebrain.org/celltypes/TEGLU9>	Excitatory neurons, cerebral cortex
9	TEGLU8 <http://mousebrain.org/celltypes/TEGLU8>	Excitatory neurons, cerebral cortex
10	TEGLU7 <http://mousebrain.org/celltypes/TEGLU7>	Excitatory neurons, cerebral cortex
11	TEGLU6 <http://mousebrain.org/celltypes/TEGLU6>	Excitatory neurons, cerebral cortex
12	TEGLU13 <http://mousebrain.org/celltypes/TEGLU13>	Excitatory neurons, cerebral cortex
13	TEGLU14 <http://mousebrain.org/celltypes/TEGLU14>	Excitatory neurons, cerebral cortex
14	TEGLU5 <http://mousebrain.org/celltypes/TEGLU5>	Excitatory neurons, cerebral cortex
15	TEGLU16 <http://mousebrain.org/celltypes/TEGLU16>	Excitatory neurons, cerebral cortex
16	TEGLU15 <http://mousebrain.org/celltypes/TEGLU15>	Excitatory neurons, cerebral cortex
17	TEGLU17 <http://mousebrain.org/celltypes/TEGLU17>	Excitatory neurons, cerebral cortex
18	TEGLU18 <http://mousebrain.org/celltypes/TEGLU18>	Excitatory neurons, cerebral cortex
19	TEGLU19 <http://mousebrain.org/celltypes/TEGLU19>	Excitatory neurons, cerebral cortex
20	TEGLU22 <http://mousebrain.org/celltypes/TEGLU22>	Excitatory neurons, amygdala
21	TEGLU21 <http://mousebrain.org/celltypes/TEGLU21>	Excitatory neurons, hippocampus CA1
22	TEGLU4 <http://mousebrain.org/celltypes/TEGLU4>	Excitatory neurons, cerebral cortex
23	TEGLU24 <http://mousebrain.org/celltypes/TEGLU24>	Excitatory neurons, hippocampus CA1
24	TEGLU23 <http://mousebrain.org/celltypes/TEGLU23>	Excitatory neurons, hippocampus CA3
25	DGGRC1 <http://mousebrain.org/celltypes/DGGRC1>	Granule neuroblasts, dentate gyrus
26	DGGRC2 <http://mousebrain.org/celltypes/DGGRC2>	Granule neurons, dentate gyrus
27	MSN1 <http://mousebrain.org/celltypes/MSN1>	D1 medium spiny neurons, striatum
28	MSN2 <http://mousebrain.org/celltypes/MSN2>	D2 medium spiny neurons, striatum
29	MSN3 <http://mousebrain.org/celltypes/MSN3>	D2 medium spiny neurons, striatum
30	MSN4 <http://mousebrain.org/celltypes/MSN4>	D1 medium spiny neurons, striatum

31	MSN5 <http://mousebrain.org/celltypes/MSN5>	Patch D1/D2 neurons, striatum
32	MSN6 <http://mousebrain.org/celltypes/MSN6>	Matrix D1 neurons, striatum
33	DETPH <http://mousebrain.org/celltypes/DETPH>	Neuroblast-like, habenula
34	DGNBL2 <http://mousebrain.org/celltypes/DGNBL2>	Granule neuroblasts, dentate gyrus
35	DGNBL1 <http://mousebrain.org/celltypes/DGNBL1>	Granule neuroblasts, dentate gyrus
36	SZNBL <http://mousebrain.org/celltypes/SZNBL>	Neuronal intermidate progenitor cells
37	OBNBL3 <http://mousebrain.org/celltypes/OBNBL3>	Neuroblasts, olfactory bulb
38	OBINH1 <http://mousebrain.org/celltypes/OBINH1>	Inhibitory neurons, olfactory bulb
39	OBINH5 http://mousebrain.org/celltypes/OBINH5	Inhibitory neurons, olfactory nulb
40	OBINH2 <http://mousebrain.org/celltypes/OBINH2>	Inner horizontal cell, olfactory bulb
41	OBINH3 <http://mousebrain.org/celltypes/OBINH3>	Inhibitory neurons, olfactory bulb
42	OBINH4 http://mousebrain.org/celltypes/OBINH4	Inhibitory neurons, olfactory bulb
43	OBNBL4 <http://mousebrain.org/celltypes/OBNBL4>	Inhibitory neurons, olfactory bulb
44	OBNBL5 <http://mousebrain.org/celltypes/OBNBL5>	Inhibitory neurons, olfactory bulb
45	OBDOP <http://mousebrain.org/celltypes/OBDOP>	Dopaminergic periglomerular interneuron, olfactory bulb
46	OBINH6 <http://mousebrain.org/celltypes/OBINH6>	External plexiform layer interneuron, olfactory bulb
47	DEINH1 http://mousebrain.org/celltypes/DEINH1	Inhibitory neurons, thalamus
48	DEINH2 <http://mousebrain.org/celltypes/DEINH2>	Inhibitory neurons, thalamus
49	TEINH17 <http://mousebrain.org/celltypes/TEINH17>	Axo-axonic, cortex/hippocampus
50	TEINH18 <http://mousebrain.org/celltypes/TEINH18>	Basket and bistratified cells, cortex/hippocampus
51	TEINH19 http://mousebrain.org/celltypes/TEINH19	Hippocamposeptal projection, cortex/hippocampus
52	TEINH21 http://mousebrain.org/celltypes/TEINH21	Sleep-active, long-range projection interneurons, cortex/hippocampus
53	TEINH16 <http://mousebrain.org/celltypes/TEINH16>	Ivy and MGE-derived neurogliaform cells, cortex/hippocampus
54	TEINH15 <http://mousebrain.org/celltypes/TEINH15>	CGE-derived neurogliaform cells, cortex/hippocampus
55	TEINH14 <http://mousebrain.org/celltypes/TEINH14>	CGE-derived neurogliaform cells Cxcl14+, cortex/hippocampus
56	TEINH20 <http://mousebrain.org/celltypes/TEINH20>	Inhibitory interneurons, hippocampus
57	TEINH13 <http://mousebrain.org/celltypes/TEINH13>	Trilaminar cells, hippocampus
58	TEINH12 <http://mousebrain.org/celltypes/TEINH12>	Non-border Cck interneurons, cortex/hippocampus
59	TEINH9 <http://mousebrain.org/celltypes/TEINH9>	Non-border Cck interneurons, hippocampus
60	TEINH10 <http://mousebrain.org/celltypes/TEINH10>	R-LM border Cck interneurons, cortex/hippocampus

61	TEINH11 <http://mousebrain.org/celltypes/TEINH11>	R-LM border Cck interneurons, cortex/hippocampus
62	TEINH4 <http://mousebrain.org/celltypes/TEINH4>	Interneuron-selective interneurons, cortex/hippocampus
63	TEINH5 <http://mousebrain.org/celltypes/TEINH5>	Interneuron-selective interneurons, cortex/hippocampus
64	TEINH8 <http://mousebrain.org/celltypes/TEINH8>	Interneuron-selective interneurons, hippocampus
65	TEINH7 <http://mousebrain.org/celltypes/TEINH7>	Interneuron-selective interneurons, hippocampus
66	TEINH6 <http://mousebrain.org/celltypes/TEINH6>	Interneuron-selective interneurons, cortex/hippocampus
67	TECHO <http://mousebrain.org/celltypes/TECHO>	Cholinergic interneurons, telencephalon
68	DECHO1 <http://mousebrain.org/celltypes/DECHO1>	Cholinergic neurons, septal nucleus, Meissnert and diagonal band
69	HBCHO4 <http://mousebrain.org/celltypes/HBCHO4>	Afferent nuclei of cranial nerves III-V
70	HBCHO3 <http://mousebrain.org/celltypes/HBCHO3>	Afferent nuclei of cranial nerves VI-XII
71	HBADR <http://mousebrain.org/celltypes/HBADR>	Adrenergic cell groups of the medulla
72	HBNOR <http://mousebrain.org/celltypes/HBNOR>	Noradrenergic neurons of the medulla
73	HYPEP7 <http://mousebrain.org/celltypes/HYPEP7>	Pmch neurons, hypothalamus
74	HYPEP6 <http://mousebrain.org/celltypes/HYPEP6>	Orexin-producing neurons, hypothalamus
75	MEGLU14 <http://mousebrain.org/celltypes/MEGLU14>	Glutamatergic projection neurons of the raphe nucleus
76	MBDOP1 http://mousebrain.org/celltypes/MBDOP1	Dopaminergic neurons, periaqueductal grey
77	MBDOP2 <http://mousebrain.org/celltypes/MBDOP2>	Dopaminergic neurons, ventral midbrain (SNc, VTA)
78	HBSER1 <http://mousebrain.org/celltypes/HBSER1>	Serotonergic neurons, hindbrain
79	HBSER2 <http://mousebrain.org/celltypes/HBSER2>	Serotonergic neurons, hindbrain
80	HBSER3 <http://mousebrain.org/celltypes/HBSER3>	Serotonergic neurons, hindbrain
81	HBSER5 http://mousebrain.org/celltypes/HBSER5	Serotonergic neurons, hindbrain
82	HBSER4 http://mousebrain.org/celltypes/HBSER4	Serotonergic neurons, hindbrain
83	TEINH3 <http://mousebrain.org/celltypes/TEINH3>	Inhibitory neurons, telencephalon
84	TEINH2 <http://mousebrain.org/celltypes/TEINH2>	Inhibitory neurons, septal nucleus
85	DEINH4 <http://mousebrain.org/celltypes/DEINH4>	Inhibitory neurons, thalamus
86	DEINH5 http://mousebrain.org/celltypes/DEINH5	Peptidergic neurons, hypothalamus
87	HYPEP3 http://mousebrain.org/celltypes/HYPEP3	Peptidergic neurons, hypothalamus
88	HYPEP1 http://mousebrain.org/celltypes/HYPEP1	Peptidergic neurons, hypothalamus
89	HYPEP2 http://mousebrain.org/celltypes/HYPEP2	Peptidergic neurons, hypothalamus
90	MEINH14 <http://mousebrain.org/celltypes/MEINH14>	Inhibitory neurons, midbrain

91	DEINH6 <http://mousebrain.org/celltypes/DEINH6>	Peptidergic neurons, hypothalamus
92	DEINH8 <http://mousebrain.org/celltypes/DEINH8>	Interneurons, hypothalamus
93	DEINH7 <http://mousebrain.org/celltypes/DEINH7>	Inhibitory neurons, hypothalamus
94	HYPEP5 <http://mousebrain.org/celltypes/HYPEP5>	Vasopressin-producing cells, hypothalamus
95	HYPEP4 <http://mousebrain.org/celltypes/HYPEP4>	Oxytocin-producing cells, hypothalamus
96	HYPEP8 <http://mousebrain.org/celltypes/HYPEP8>	Peptidergic neurons, hypothalamus
97	SCINH11 <http://mousebrain.org/celltypes/SCINH11>	Central canal neurons, spinal cord
98	SCINH10 http://mousebrain.org/celltypes/SCINH10	Inhibitory neurons, spinal cord
99	SCINH9 http://mousebrain.org/celltypes/SCINH9	Inhibitory neurons, spinal cord
100	SCINH8 <http://mousebrain.org/celltypes/SCINH8>	Inhibitory neurons, spinal cord
101	SCINH7 http://mousebrain.org/celltypes/SCINH7	Inhibitory neurons, spinal cord
102	SCINH6 http://mousebrain.org/celltypes/SCINH6	Inhibitory neurons, spinal cord
103	SCINH5 <http://mousebrain.org/celltypes/SCINH5>	Inhibitory neurons, spinal cord
104	SCINH4 http://mousebrain.org/celltypes/SCINH4	Inhibitory neurons, spinal cord
105	SCINH3 http://mousebrain.org/celltypes/SCINH3	Inhibitory neurons, spinal cord
106	HBINH9 <http://mousebrain.org/celltypes/HBINH9>	Inhibitory neurons, hindbrain
107	SCINH2 <http://mousebrain.org/celltypes/SCINH2>	Inhibitory neurons, spinal cord
108	SCGLU1 <http://mousebrain.org/celltypes/SCGLU1>	Excitatory neurons, spinal cord
109	SCGLU2 <http://mousebrain.org/celltypes/SCGLU2>	Excitatory neurons, spinal cord
110	SCGLU3 <http://mousebrain.org/celltypes/SCGLU3>	Excitatory neurons, spinal cord
111	SCGLU4 <http://mousebrain.org/celltypes/SCGLU4>	Excitatory neurons, spinal cord
112	SCGLU5 <http://mousebrain.org/celltypes/SCGLU5>	Excitatory neurons, spinal cord
113	SCGLU6 <http://mousebrain.org/celltypes/SCGLU6>	Excitatory neurons, spinal cord
114	SCGLU7 http://mousebrain.org/celltypes/SCGLU7	Excitatory neurons, spinal cord
115	SCGLU8 <http://mousebrain.org/celltypes/SCGLU8>	Excitatory neurons, spinal cord
116	SCGLU9 <http://mousebrain.org/celltypes/SCGLU9>	Excitatory neurons, spinal cord
117	SCGLU10 http://mousebrain.org/celltypes/SCGLU10	Excitatory neurons, spinal cord
118	HBGLU10 <http://mousebrain.org/celltypes/HBGLU10>	Excitatory neurons, hindbrain
119	HBGLU3 <http://mousebrain.org/celltypes/HBGLU3>	Excitatory neurons, hindbrain
120	HBGLU2 <http://mousebrain.org/celltypes/HBGLU2>	Excitatory neurons, hindbrain
121	MEGLU2 <http://mousebrain.org/celltypes/MEGLU2>	Excitatory neurons, midbrain
122	MEGLU3 <http://mousebrain.org/celltypes/MEGLU3>	Excitatory neurons, midbrain
123	DEGLU5 <http://mousebrain.org/celltypes/DEGLU5>	Excitatory neurons, midbrain
124	MEGLU1 <http://mousebrain.org/celltypes/MEGLU1>	Excitatory neurons, midbrain
125	MEGLU7 <http://mousebrain.org/celltypes/MEGLU7>	Excitatory neurons, midbrain
126	MEGLU8 <http://mousebrain.org/celltypes/MEGLU8>	Excitatory neurons, midbrain
127	MEGLU9 <http://mousebrain.org/celltypes/MEGLU9>	Excitatory neurons, midbrain
128	MEGLU10 <http://mousebrain.org/celltypes/MEGLU10>	Excitatory neurons, midbrain
129	MEGLU11 <http://mousebrain.org/celltypes/MEGLU11>	Excitatory neurons, midbrain
130	MBCHO1 <http://mousebrain.org/celltypes/MBCHO1>	Cholinergic neurons, midbrain red nucleus

131	MEGLU6 <http://mousebrain.org/celltypes/MEGLU6>	Excitatory neurons, midbrain
132	MEGLU5 http://mousebrain.org/celltypes/MEGLU5	Excitatory neurons, midbrain
133	MEGLU4 <http://mousebrain.org/celltypes/MEGLU4>	Excitatory neurons, midbrain
134	CR http://mousebrain.org/celltypes/CR	Cajal-Retzius cells, hippocampus
135	DECHO2 <http://mousebrain.org/celltypes/DECHO2>	Cholinergic neurons, habenula
136	HBGLU1 http://mousebrain.org/celltypes/HBGLU1	Excitatory neurons, hindbrain
137	DEGLU1 http://mousebrain.org/celltypes/DEGLU1	Excitatory neurons, thalamus
138	DEGLU2 <http://mousebrain.org/celltypes/DEGLU2>	Excitatory neurons, hypothalamus
139	DEGLU3 <http://mousebrain.org/celltypes/DEGLU3>	Excitatory neurons, thalamus
140	DEGLU4 <http://mousebrain.org/celltypes/DEGLU4>	Excitatory neurons, thalamus
141	MEINH12 <http://mousebrain.org/celltypes/MEINH12>	Inhibitory neurons, midbrain
142	MEINH11 <http://mousebrain.org/celltypes/MEINH11>	Inhibitory neurons, midbrain
143	MEINH10 <http://mousebrain.org/celltypes/MEINH10>	Inhibitory neurons, midbrain
144	MEINH9 <http://mousebrain.org/celltypes/MEINH9>	Inhibitory neurons, midbrain
145	MEINH5 <http://mousebrain.org/celltypes/MEINH5>	Inhibitory neurons, midbrain
146	MEINH6 http://mousebrain.org/celltypes/MEINH6	Inhibitory neurons, midbrain
147	MEINH7 <http://mousebrain.org/celltypes/MEINH7	Inhibitory neurons, midbrain
148	MEINH4 <http://mousebrain.org/celltypes/MEINH4>	Inhibitory neurons, midbrain
149	MEINH3 <http://mousebrain.org/celltypes/MEINH3>	Inhibitory neurons, midbrain
150	HBINH5 <http://mousebrain.org/celltypes/HBINH5>	Inhibitory neurons, hindbrain
151	MEINH2 <http://mousebrain.org/celltypes/MEINH2>	Inhibitory neurons, midbrain
152	DEINH3 http://mousebrain.org/celltypes/DEINH3	Inhibitory neurons, hypothalamus
153	TEINH1 <http://mousebrain.org/celltypes/TEINH1>	Inhibitory neurons, pallidum
154	MEINH13 <http://mousebrain.org/celltypes/MEINH13>	Inhibitory neurons, midbrain
155	MEINH8 <http://mousebrain.org/celltypes/MEINH8>	Inhibitory neurons, midbrain
156	HBINH1 <http://mousebrain.org/celltypes/HBINH1>	Inhibitory neurons, hindbrain
157	HBINH3 <http://mousebrain.org/celltypes/HBINH3>	Inhibitory neurons, hindbrain
158	HBINH4 <http://mousebrain.org/celltypes/HBINH4>	Inhibitory neurons, hindbrain
159	HBINH6 <http://mousebrain.org/celltypes/HBINH6>	Inhibitory neurons, hindbrain
160	HBINH2 http://mousebrain.org/celltypes/HBINH2	Inhibitory neurons, hindbrain
161	HBCHO1 <http://mousebrain.org/celltypes/HBCHO1>	Cholinergic neurons, hindbrain
162	HBCHO2 <http://mousebrain.org/celltypes/HBCHO2>	Cholinergic neurons, hindbrain
163	HBGLU4 <http://mousebrain.org/celltypes/HBGLU4>	Excitatory neurons, hindbrain
164	HBGLU5 <http://mousebrain.org/celltypes/HBGLU5>	Excitatory neurons, hindbrain
165	HBGLU6 <http://mousebrain.org/celltypes/HBGLU6>	Excitatory neurons, hindbrain

166 HBGLU7 <http://mousebrain.org/celltypes/HBGLU7> Excitatory neurons, hindbrain
167 HBGLU8 http://mousebrain.org/celltypes/HBGLU8 Excitatory neurons, hindbrain
168 HBGLU9 http://mousebrain.org/celltypes/HBGLU9 Excitatory neurons, hindbrain
169 HBINH7 <http://mousebrain.org/celltypes/HBINH7> Inhibitory neurons, hindbrain
170 HBINH8 <http://mousebrain.org/celltypes/HBINH8> Inhibitory neurons, hindbrain
171 SCINH1 <http://mousebrain.org/celltypes/SCINH1> Inhibitory neurons, spinal cord
172 CBINH2 <http://mousebrain.org/celltypes/CBINH2> Granular layer interneurons, cerebellum
173 MEINH1 <http://mousebrain.org/celltypes/MEINH1> Inhibitory neurons, midbrain
174 CBINH1 <http://mousebrain.org/celltypes/CBINH1> Molecular layer interneurons, cerebellum
175 CBPC <http://mousebrain.org/celltypes/CBPC> Purkinje cells
176 CBGRC <http://mousebrain.org/celltypes/CBGRC> Granule neurons, cerebellum
177 CBNBL2 <http://mousebrain.org/celltypes/CBNBL2> Neuroblasts, cerebellum
178 CBNBL1 <http://mousebrain.org/celltypes/CBNBL1> Neuroblasts, cerebellum
179 SEPNBL <http://mousebrain.org/celltypes/SEPNBL> Neuroblasts, septum
180 OBNBL1 <http://mousebrain.org/celltypes/OBNBL1> Neuroblasts, olfactory
181 OBNBL2 <http://mousebrain.org/celltypes/OBNBL2> Neuroblasts, olfactory bulb
182 ENT1 <http://mousebrain.org/celltypes/ENT1> Nitrergic enteric neurons
183 ENT2 http://mousebrain.org/celltypes/ENT2 Nitrergic enteric neurons
184 ENT3 http://mousebrain.org/celltypes/ENT3 Nitrergic enteric neurons
185 ENT4 http://mousebrain.org/celltypes/ENT4 Cholinergic enteric neurons
186 ENT5 <http://mousebrain.org/celltypes/ENT5> Cholinergic enteric neurons
187 ENT6 <http://mousebrain.org/celltypes/ENT6> Cholinergic enteric neurons
188 ENT7 <http://mousebrain.org/celltypes/ENT7> Cholinergic enteric neurons, VGLUT2
189 ENT8 <http://mousebrain.org/celltypes/ENT8> Cholinergic enteric neurons, VGLUT2
190 ENT9 <http://mousebrain.org/celltypes/ENT9> Cholinergic enteric neurons
191 SYNOR1 <http://mousebrain.org/celltypes/SYNOR1> Noradrenergic erector muscle neurons
192 SYNOR2 <http://mousebrain.org/celltypes/SYNOR2> Noradrenergic neurons, sympathetic
193 SYNOR3 <http://mousebrain.org/celltypes/SYNOR3> Noradrenergic neurons, sympathetic
194 SYNOR4 <http://mousebrain.org/celltypes/SYNOR4> Noradrenergic erector muscle neurons 195
SYNOR5 <http://mousebrain.org/celltypes/SYNOR5> Noradrenergic erector muscle neurons
196 SYCHO2 <http://mousebrain.org/celltypes/SYCHO2> Cholinergic neurons, sympathetic
197 SYCHO1 <http://mousebrain.org/celltypes/SYCHO1> Cholinergic neurons, sympathetic
198 PSPEP8 <http://mousebrain.org/celltypes/PSPEP8> Peptidergic (TrpM8), DRG
199 PSPEP7 <http://mousebrain.org/celltypes/PSPEP7> Peptidergic (TrpM8), DRG
200 PSPEP6 <http://mousebrain.org/celltypes/PSPEP6> Peptidergic (TrpM8), DRG

201	PSPEP5 <http://mousebrain.org/celltypes/PSPEP5>	Peptidergic (PEP1.2), DRG
202	PSPEP2 <http://mousebrain.org/celltypes/PSPEP2>	Peptidergic (PEP1.3), DRG
203	PSPEP4 <http://mousebrain.org/celltypes/PSPEP4>	Peptidergic (PEP1.1), DRG
204	PSPEP3 <http://mousebrain.org/celltypes/PSPEP3>	Peptidergic (PEP1..4), DRG
205	PSPEP1 <http://mousebrain.org/celltypes/PSPEP1>	Peptidergicv (PEP2), DRG
206	PSNF3 <http://mousebrain.org/celltypes/PSNF3>	Neurofilament (NF2/3), DRG
207	PSNF2 <http://mousebrain.org/celltypes/PSNF2>	Neurofilament (NF4/5), DRG
208	PSNF1 http://mousebrain.org/celltypes/PSNF1	Neurofilament (NF1), DRG
209	PSNP1 <http://mousebrain.org/celltypes/PSNP1>	Non-peptidergic (TH), DRG
210	PSNP2 <http://mousebrain.org/celltypes/PSNP2>	Non-peptidergic (NP1.1), DRG
211	PSNP3 <http://mousebrain.org/celltypes/PSNP3>	Non-peptidergic (NP1.2), DRG
212	PSNP4 <http://mousebrain.org/celltypes/PSNP4>	Non-peptidergic (NP2.1), DRG
213	PSNP5 <http://mousebrain.org/celltypes/PSNP5>	Non-peptidergic (NP2.2), DRG
214	PSNP6 <http://mousebrain.org/celltypes/PSNP6>	Non-peptidergic (NP3), DRG
215	COP1 <http://mousebrain.org/celltypes/COP1>	Committed oligodendrocytes cells (COP)
216	COP2 <http://mousebrain.org/celltypes/COP2>	Committed oligodendrocytes cells (COP), pons/medulla specific
217	NFOL2 <http://mousebrain.org/celltypes/NFOL2>	Newly formed oligodendrocytes (NFOL), pons/medulla specific
218	NFOL1 <http://mousebrain.org/celltypes/NFOL1>	Newly formed oligodendrocytes (NFOL)
219	MFOL2 <http://mousebrain.org/celltypes/MFOL2>	Myelin forming oligodendrocytes (MFOL)
220	MFOL1 <http://mousebrain.org/celltypes/MFOL1>	Myelin forming oligodendrocytes (MFOL)
221	MOL1 <http://mousebrain.org/celltypes/MOL1>	Mature oligodendrocytes
222	MOL2 <http://mousebrain.org/celltypes/MOL2>	Mature oligodendrocytes, hindbrain
223	MOL3 <http://mousebrain.org/celltypes/MOL3>	Mature oligodendrocytes, spinal cord enriched (high Klk6)
224	CHOR <http://mousebrain.org/celltypes/CHOR>	Chorid plexus epithelial cells
225	HYPEN <http://mousebrain.org/celltypes/HYPEN>	Hypendymal cell, subcommissural organ
226	EPSC <http://mousebrain.org/celltypes/EPSC>	Ependymal cells, spinal cord
227	EPEN <http://mousebrain.org/celltypes/EPEN>	Ependymal cells
228	EPMB <http://mousebrain.org/celltypes/EPMB>	Ependymal cells, midbrain
229	RGDG <http://mousebrain.org/celltypes/RGDG>	Dentate gyrus radial glia-like cells
230	RGSZ <http://mousebrain.org/celltypes/RGSZ>	Subventricular zone radial glia-like cells
231	ACTE1 <http://mousebrain.org/celltypes/ACTE1>	Telencephalon astrocytes, fibrous
232	ACTE2 <http://mousebrain.org/celltypes/ACTE2>	Telencephalon astrocytes, protoplasmic
233	ACOB <http://mousebrain.org/celltypes/ACOB>	Olfactory astrocytes
234	ACNT1 <http://mousebrain.org/celltypes/ACNT1>	Non-telencephalon astrocytes, protoplasmic 235
234	ACNT2 <http://mousebrain.org/celltypes/ACNT2>	Non-telencephalon astrocytes, fibrous

36	ACMB <http://mousebrain.org/celltypes/ACMB>	Dorsal midbrain Myoc-expressing astrocyte-like
37	ACBG <http://mousebrain.org/celltypes/ACBG>	Bergmann glia
38	OEC <http://mousebrain.org/celltypes/OEC>	Olfactory ensheathing cells
39	OPC <http://mousebrain.org/celltypes/OPC>	Oligodendrocytes precursor cells
40	SCHW <http://mousebrain.org/celltypes/SCHW>	Schwann cells
41	SATG2 <http://mousebrain.org/celltypes/SATG2>	Satellite glia
42	SATG1 <http://mousebrain.org/celltypes/SATG1>	Satellite glia, proliferating
43	ENTG1 <http://mousebrain.org/celltypes/ENTG1>	Enteric glia, proliferating
44	ENTG2 <http://mousebrain.org/celltypes/ENTG2>	Enteric glia
45	ENTG3 <http://mousebrain.org/celltypes/ENTG3>	Enteric glia
46	ENTG4 <http://mousebrain.org/celltypes/ENTG4>	Enteric glia
47	ENTG5 <http://mousebrain.org/celltypes/ENTG5>	Enteric glia
48	ENTG6 <http://mousebrain.org/celltypes/ENTG6>	Enteric glia
49	ENTG7 <http://mousebrain.org/celltypes/ENTG7>	Enteric glia
50	ENMFB http://mousebrain.org/celltypes/ENMFB	Enteric mesothelial fibroblasts
51	ABC <http://mousebrain.org/celltypes/ABC>	Vascular leptomeningeal cells
52	VLMC2 <http://mousebrain.org/celltypes/VLMC2>	Vascular leptomeningeal cells
53	VLMC1 <http://mousebrain.org/celltypes/VLMC1>	Vascular leptomeningeal cells
54	VECA <http://mousebrain.org/celltypes/VECA>	Vascular endothelial cells, arterial
55	PER3 <http://mousebrain.org/celltypes/PER3>	Pericytes
56	VSMCA <http://mousebrain.org/celltypes/VSMCA>	Vascular smooth muscle cells, arterial
57	PER1 <http://mousebrain.org/celltypes/PER1>	Pericytes
58	PER2 <http://mousebrain.org/celltypes/PER2>	Pericytes, possibly mixed with VENC
59	VECC <http://mousebrain.org/celltypes/VECC>	Vascular endothelial cells, capillary
60	VECV <http://mousebrain.org/celltypes/VECV>	Vascular endothelial cells, venous
61	PVM1 <http://mousebrain.org/celltypes/PVM1>	Perivascular macrophages
62	PVM2 <http://mousebrain.org/celltypes/PVM2>	Perivascular macrophages, activated
63	MGL3 <http://mousebrain.org/celltypes/MGL3>	Microglia, activated
64	MGL2 <http://mousebrain.org/celltypes/MGL2>	Microglia, activated
65	MGL1 <http://mousebrain.org/celltypes/MGL1>	Microglia
